# Trends in Disease Burden of Chronic Lymphocytic Leukemia at the Global, Regional, and National Levels From 1990 to 2019, and Projections Until 2030: A Population-Based Epidemiologic Study

**DOI:** 10.3389/fonc.2022.840616

**Published:** 2022-03-10

**Authors:** Yang Ou, Yichen Long, Lili Ji, Yanxia Zhan, Tiankui Qiao, Xiangdong Wang, Hao Chen, Yunfeng Cheng

**Affiliations:** ^1^ Center for Tumor Diagnosis & Therapy, Jinshan Hospital, Fudan University, Shanghai, China; ^2^ School of Life Science, University of Chinese Academy of Sciences, Beijing, China; ^3^ Department of Hematology, Zhongshan Hospital, Fudan University, Shanghai, China; ^4^ Institute of Clinical Science, Zhongshan Hospital, Fudan University, Shanghai, China; ^5^ Department of Thoracic Surgery, Zhongshan Hospital Xuhui Branch, Fudan University, Shanghai, China; ^6^ Department of Hematology, Zhongshan Hospital Qingpu Branch, Fudan University, Shanghai, China

**Keywords:** chronic lymphocytic leukemia, epidemiology, incidence, death, disability-adjusted life years

## Abstract

**Background:**

The prognosis of chronic lymphocytic leukemia (CLL) has been improved dramatically, but there are limited studies focusing on CLL disease burden on a global scale. We aimed to evaluate the accurate assessment of the disease burden of CLL that may provide more detailed epidemiological information for rational policies.

**Methods:**

The main source of the data was the Global Burden of Disease (GBD) study 2019. Incident cases, death cases, disability-adjusted life years (DALYs), and their corresponding age-standardized rates (ASRs) from 1990 to 2019 were used to describe the burden of CLL. Data about attributable risk factors were also extracted and analyzed. Bayesian age-period-cohort (BAPC) models were used to assess and project the incidence and mortality rates till 2030.

**Results:**

Globally, the incidence of CLL had been increasing. Deaths and DALYs decreased slightly. The burden of death and DALY is affected by socio-demographic index (SDI). The incidence rate, death rate, and DALY rate of CLL increased significantly with age. Male-to-female ratios of incidence rates varied in different SDI quintiles. Smoking, high body mass index, and occupational exposure to benzene or formaldehyde were the potential risk factors related to CLL. Global ASIRs might tend to increase until 2030, while ASDR would decrease until 2030.

**Conclusion:**

The disease burden of CLL decreased in higher SDI countries but increased in lower ones. Strategies for early detection of asymptomatic CLL, development of novel drugs, and measures against attributable factors should be implemented to combat CLL burden.

## Introduction

Chronic lymphocytic leukemia (CLL) is a mature B-cell neoplasm characterized by the progressive accumulation of monoclonal B lymphocytes. It is the most prevalent type of leukemia in the Western hemisphere, accounting for approximately 25% to 35% of all leukemias in the United States (1). An epidemiologic profile of CLL has been established, such that the disorder is more common in men with a male-to-female ratio of approximately 1.2:1 to 1.7:1 (1, 2). It is considered to be a disease of older adults, with a median age of approximately 70 years at diagnosis (3). The incidence of CLL varies by race and geographic location, which is higher among Caucasians (2, 4) and extremely low in Asia (5, 6).

The development of novel targeted drugs for CLL, including Bruton’s tyrosine kinase inhibitors (BTKi; ibrutinib and acalabrutinib), BCL2 inhibitors (venetoclax), and phosphatidylinositol 3-kinase inhibitors (PI3Ki; idelalisib and duvelisib), has fundamentally changed the CLL therapy landscape (7) and dramatically improves the prognosis of CLL patients in developed countries. Five-year overall survival has been more than 60% in the era of BTKi (8–10), and is about 86% after the advent of venetoclax (11). However, access to these novel agents is still limited to certain developed countries, so every effort should be made to ensure patients of developing countries could also benefit from these outstanding medicines (12). While rational policymaking in the era of novel agents is pivotal, there remains paucity of literature to assess the disease burden of CLL in global, regional, and national scope, as well as to review the changes over the past 30 years.

The Global Burden of Disease (GBD) study launched by the Global Burden of Disease Collaborative Network assesses 286 causes of death, 369 diseases and injuries, and 87 risk factors in 204 countries and territories (13). Several studies have used GBD data to estimate the disease burden of leukemia (14–16), but none of them focused on CLL. In addition, studies predicting the disease burden of CLL based on GBD study results are scarce. In this study, we collected CLL data between 1990 and 2019 from the GBD 2019 study, including incidence, disease-related mortality, disability-adjusted life years (DALYs), their corresponding age-standardized rates (ASRs), and their attributable risk factors across gender, age, socio-demographic index (SDI), region, and country. Furthermore, we used change percentages from 1990 to 2019 and estimated annual percentage changes (EAPCs) to quantify the trends of ASRs. We aimed to assess the accurate assessment of the distribution, burden, and trends of CLL in different regions and countries, then project CLL’s disease burden until 2030, which would provide more detailed epidemiological information and formulate more rational policies. As such, the aim of the present study is to examine the accurate assessment of the distribution, burden, and trends of CLL in different regions and nations, and then project CLL disease burden until 2030, which could provide important epidemiological information for healthcare policymaking.

## Materials and Methods

### Data Sources

Data on CLL was collected from the latest version of the GBD study by using the Global Health Data Results tool (http://ghdx.healthdata.org/gbd-results-tool) (13). The protocol used for GBD 2019 was posted on the website of the Institute for Health Metrics and Evaluation (http://www.healthdata.org/sites/default/files/files/Projects/GBD/March2020_GBD%20Protocol_v4.pdf). This study was compliant with the Guidelines for Accurate and Transparent Health Estimates Reporting (GATHER) (17). According to the instruction, the number and rate of incidence, death, and DALY of CLL were extracted between 1990 and 2019 based on age, sex, SDI, region, and country, without any inclusion/exclusion criteria. According to the geographical and socioeconomical features, the world was divided into 21 regions, including North America, East Asia, South Asia, and Eastern Europe. The SDI is a composite indicator of development status strongly correlated with health outcomes. It is the geometric mean of zero-to-one indices of total fertility rate under the age of 25 (TFU25), mean education for those ages 15 and older (EDU15+), and lag distributed income (LDI) per capita (18). According to SDI, areas were categorized into 5 levels, including low, low-middle, middle, high-middle, and high. Data were available for 204 countries/territories, including China, India, and France. The methodologies of the overall GBD 2019 and estimations of disease burden were as described in the previous studies (15, 16, 19).

### Statistical Analysis

Calculations of ASR were based on the age structure of the standard populations, so the ASR was a necessary and representative index considering the differences in the age structure of multiple populations. The ASR (per 100,000 populations) was calculated using the following formula:


$$ASR=\frac{\Sigma_{i=1}^{A}\;a_{i}w_{i}}{\Sigma_{i=1}^{A}\;w_{i}}\times 100,000$$

In the formula, *a*_i_ denotes the age-specific rates in the *i*th age group, *w*_i_ denotes the number of persons (or the weight) in the corresponding *i*th age subgroup of the selected reference standard population, and *A* denotes the number of age groups.

The EAPCs were used to evaluate the trends of ASRs, which were calculated using a regression model: *y* = *α* + *βx* + *ϵ* (*y* = ln (ASR), *x* = calendar year, and *ϵ* = error term). EAPC = 100 × (exp (*β*) − 1) and its 95% UI were obtained from the regression model (16, 20, 21). If the EAPC and lower limit of UI were negative values, the incidence rate was considered to have a descending trend; in contrast, if the EAPC and upper limit of UI were positive, the incidence rate was considered to have an ascending trend. Pearson correlation analysis and loess local weighted regression (LOESS) were used to examine the correlation. All calculations and analyses were performed using the R software (version 3.6.3). All tests were two-tailed, and a *p*-value of <0.05 was considered statistically significant.

### Model Selection and Application

Several models were used to predict the incidence or mortality rate of cancers based on population data, including Joinpoint (22), age-period-cohort (APC) model (23), Nordpred model (24), and Bayesian APC model (BAPC) (25). Zhebin Du et al. applied 5 models above the GBD data to predict incidence and mortality rates and assessed their performances, finally finding BAPC with a relatively lower error rate (26). Knoll and colleagues compared predictive performances of five models, including BAPC, generalized linear models (GLMs), and generalized additive models (GALs) with age and period as covariates and found that BAPC models had the highest coverage (calculated as the fraction of projections with 95% uncertainty interval [UI]) (27).

APC models analyze registry data according to the age group of the individual, the date of the event that was considered (period), and the birth cohort of the individual (28). Bayesian APC models are particularly useful to project future cancer burden as they involve no parametric assumptions. Bray provided a comparison of projections derived from linear power models, as well as the classical and Bayesian version of the APC model, and concluded that the Bayesian APC model could achieve more sensible projections (29). An R package “BAPC” developed by Andrea Riebler and Leonard Held (30), which were based on integrated nested Laplace approximations, had been widely used to project GBD data (26, 31, 32). We utilized “BAPC” in our study to project the incidence and mortality rates until 2030, based on the incidence and death cases of CLL classified by age group from 1990 to 2019 in GBD dataset, and estimates or standard projection variants of the population classified by age group from World Population Prospects Project 2019 (https://population.un.org/wpp/Download/Standard/Population/). Data of the world and 5 individual countries including USA, Ukraine, China, India, and Afghanistan (which represent 5 different SDI levels respectively) were included.

## Results

### The Incidence of CLL and Its Trend

**Figure 1:**
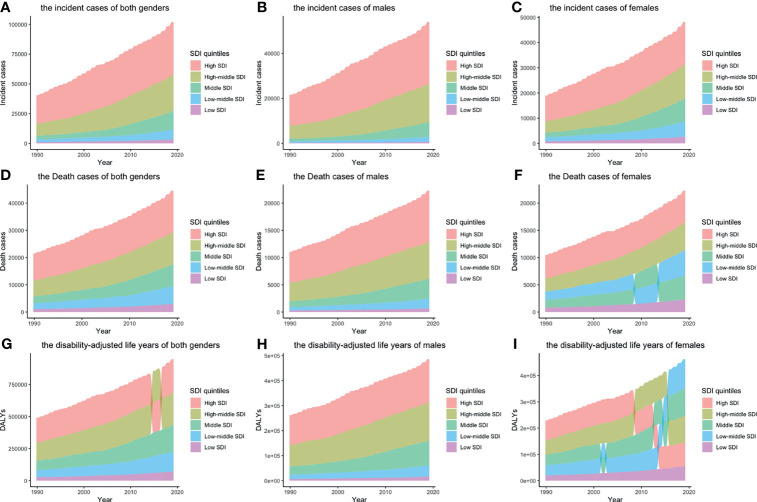
Incident cases, death cases, and DALYs of different SDI quintiles from 1990 to 2019 by gender: **(A)** incident cases of both genders; **(B)** incident cases of males; **(C)** incident cases of females; **(D)** death cases of both genders; **(E)** death cases of males; **(F)** death cases of females; **(G)** DALYs of both genders; H: DALYs of males; I: DALYs of females. Alluvia represent numbers of different SDI quintiles, from the highest (the SDI quintile with largest number) to the lowest (the SDI quintile with smallest number). (DALY, disability-adjusted life year; SDI, socio-demographic index).

**Figure 2:**
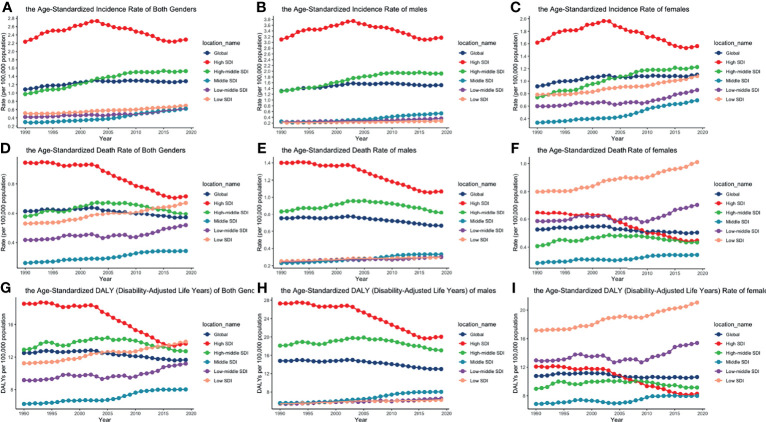
ASIRs, ASDRs, and age-standardized DALY rates of different SDI quintiles from 1990 to 2019 by gender: **(A)** ASIRs of both genders; **(B)** ASIRs of males; **(C)** ASIRS of females; **(D)** ASDRs of both genders; **(E)** ASDRs of males; **(F)** ASDRs of females; **(G)** age-standardized DALY rates of both genders; **(H)** age-standardized DALY rates of males; **(I)** age-standardized DALY rates of females. (ASIR, age-standardized incidence rate; ASDR, age-standardized death rate; DALY, disability-adjusted life year; SDI, socio-demographic index).

**Figure 3:**
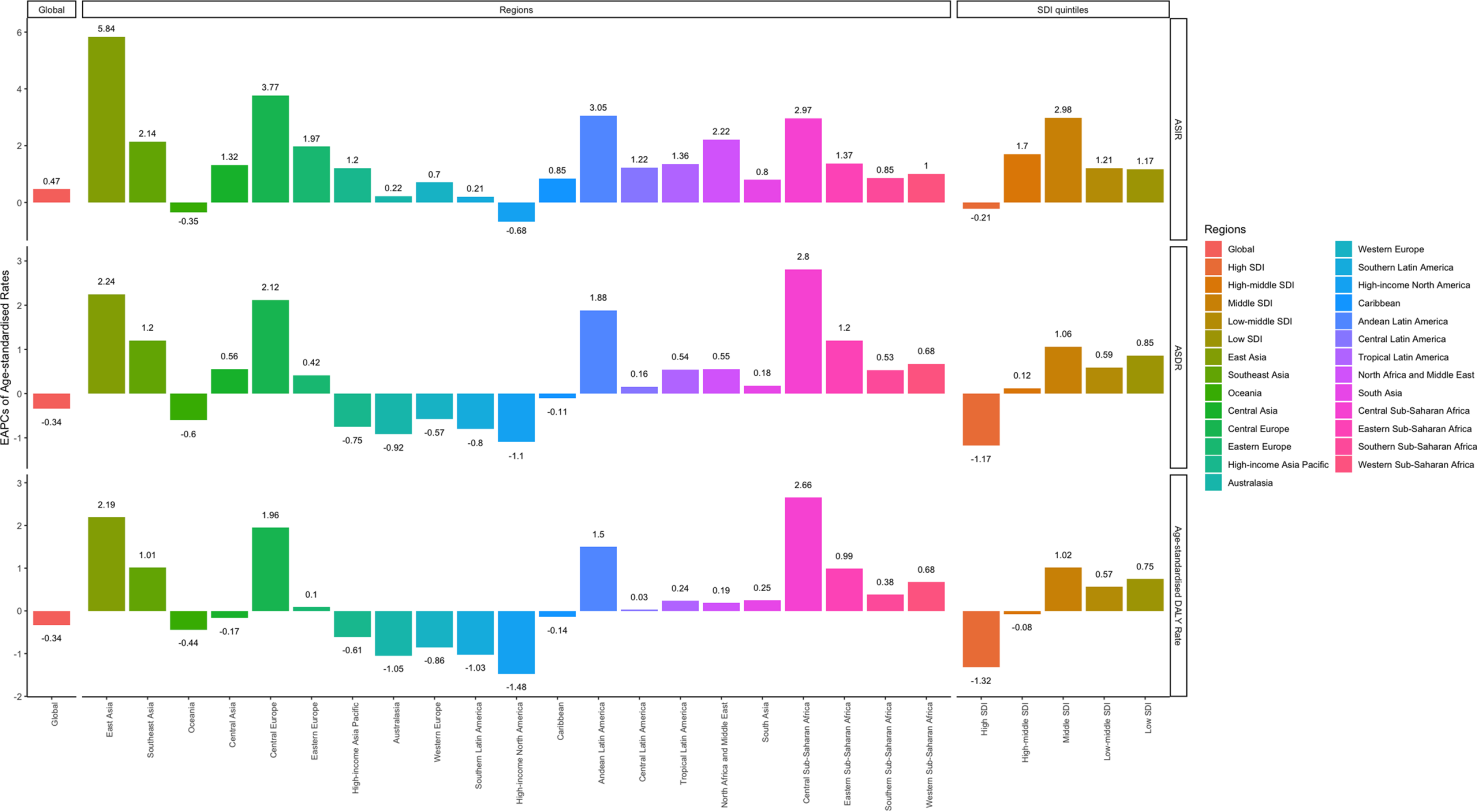
EAPCs of ASIR (upper), ASDRs (middle), and age-standardized DALY rates (lower), in the global (left), regional (middle) and SDI (right) levels. (ASIR, age-standardized incidence rate; ASDR, age-standardized death rate; DALY, disability-adjusted life year; SDI, socio-demographic index).

**Figure 4:**
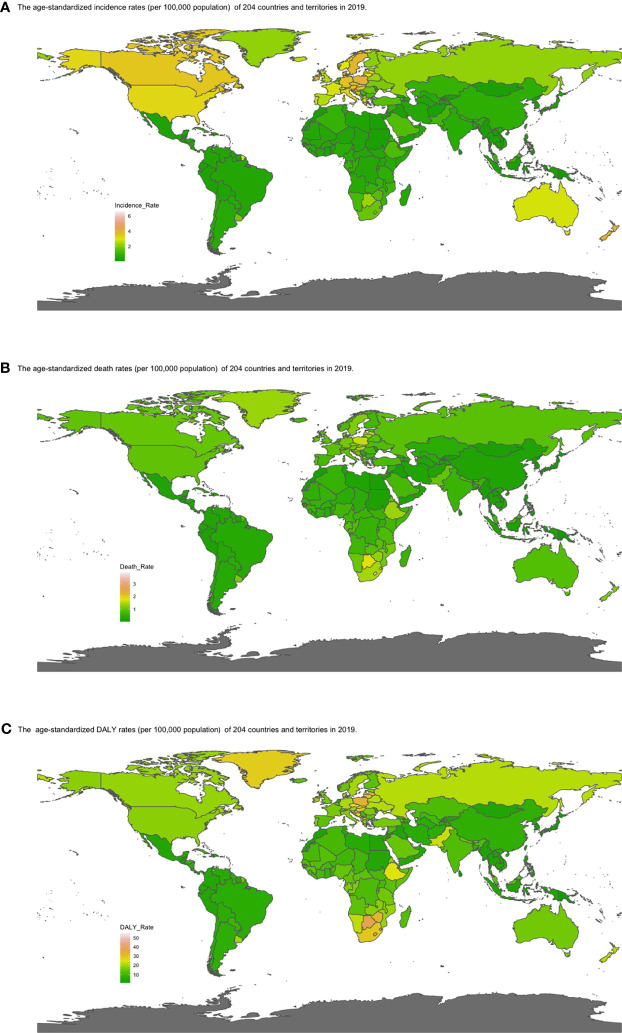
Map of **(A)** ASIR, **(B)** ASDRs, and **(C)** age-standardized DALY rates by country in 2019. Heat gradient represents age-standardized rates from pink (highest) to dark green (lowest). (ASIR, age-standardized incidence rate; ASDR, age-standardized death rate; DALY, disability-adjusted life year; EAPC, estimated annual percentage change).

**Figure 5:**
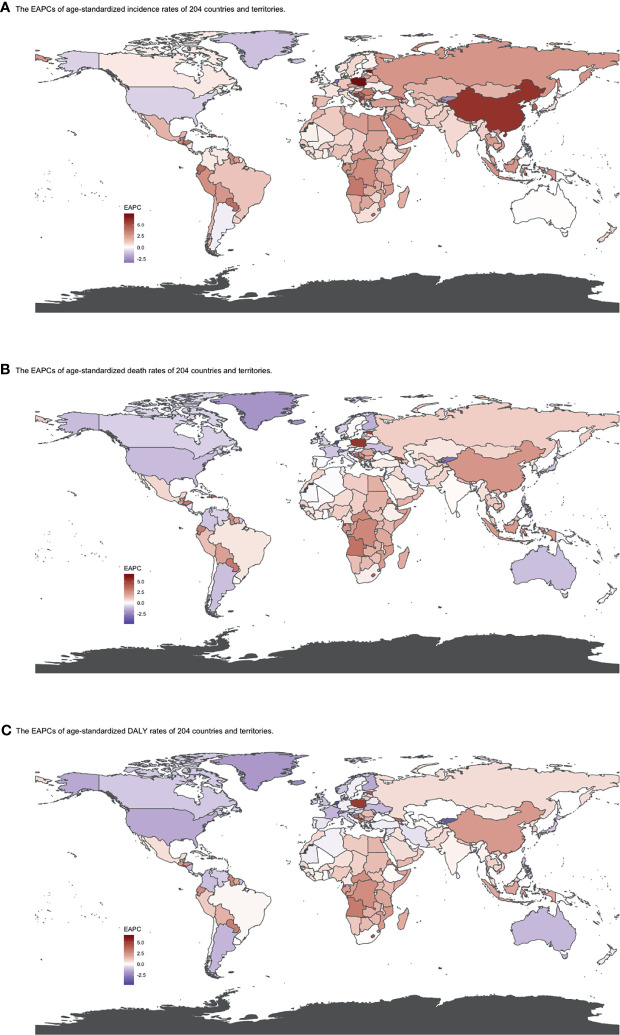
EAPCs of **(A)** ASIR, **(B)** ASDRs, and **(C)** age-standardized DALY rates by country from 1990 to 2019. Heat gradient represents the change trends of EAPCs from red (highest) to blue (lowest). Blue indicates a downward trend and Red indicates an upward trend. (ASIR, age-standardized incidence rate; ASDR, age-standardized death rate; DALY, disability-adjusted life year; EAPC, estimated annual percentage change).

The global incident number of chronic lymphocytic leukemia was 1,034.67 ×10^2^ [95% uncertainty interval (UI) = (934.64, 1,189.42)] in 2019, with a total increase of 155.24% from 1990 (**Figures 1A–C**, **Table 1** and **Supplementary Table S1**). The global age-standardized incidence rate (ASIR) was 1.28 (1.16, 1.48) per 100,000, and it showed an increasing trend with an annual average of 0.47% [EAPC = 0.47; 95% CI = (0.33, 0.61)] from 1990 to 2019 (**Figures 2A–C**, **Figure 3** upper, **Table 1** and **Supplementary Table S4**). In 1990, the high SDI quintile had an ASIR of 2.24 (2.05, 2.39)/100,000 persons, which then increased and peaked at 2.74 (2.51, 3.05)/100,000 persons in 2003. After that, the ASIR of the high SDI quintile dropped to its lowest level of 2.24 (2.02, 2.74)/100,000 persons in 2017. In recent years, the ASIR of the high SDI quintile rose slightly again (**Table 1**, **Figures 2A–C** and **Supplementary Table S4**). Except for the high SDI quintile, other SDI quintiles all had increasing trends of ASIRs. According to their EAPCs of ASIRs, the middle SDI quintile had the most obvious increase [EAPC = 2.98 (2.76, 3.19)] (**Table 1**, **Figures 2A–C** and **Figure 3** upper). In terms of geographical regions, High-income North America with an ASIR of 3.19 (2.69, 3.94)/100,000 persons, and Central Europe with an ASIR of 3 (2.53, 3.68)/100,000 persons in 2019 remained the top two highest incidence rates in the world. From 1990 to 2019, the EAPCs of ASIRs showed an increasing trend in most geographical regions except in High-income North America [EAPC = −0.68 (−0.96, −0.41)] and Oceania [EAPC = −0.35 (−0.46, −0.24)]. Among them, East Asia [EAPC = 5.84 (5.41, 6.26)] and Central Europe [EAPC = 3.77 (3.29, 4.24)] had the highest increasing speed (**Table 1** and **Figure 3** upper). Regarding observation of countries and territories, in 2019, the top three countries with the most incident cases of CLL were the United States of America (183.19 (152.66, 228.91) ×10^2^), China (159.1 (130.45, 194.43) ×10^2^), and India (76.73 (63.3, 91.63) ×10^2^) (**Supplementary Table S4**). Qatar (6.57 (4.64, 9.31)/100,000 persons), Israel (4.53 (3.34, 6.14)/100,000 persons), and Croatia (4.47 (3.34, 6.05)/100,000 persons) had the highest ASIRs in 2019 (**Figure 4A**, **Supplementary Table S7**). Poland (EAPC = 7.48 (6.25, 8.74)) and Netherlands (EAPC = −3.35 (−4.69, −2)) had the most increase and decrease in ASIRs, respectively (**Figure 5A**, **Supplementary Table S10**).

**Table 1:** The incident cases and ASIRs of CLL in 1990 and 2019, and the trends from 1990 to 2019.

		Incident cases (×10^2^, 95% UI)			ASIRs per 100,000 (95% UI)		
		1990	2019	Change Percentages of Cases (%)	1990	2019	EAPCs of ASIRs (95% CI)
Global		405.37 (371.18, 427.52)	1,034.67 (934.64, 1,189.42)	155.24	1.31 (1.15, 1.4)	1.28 (1.16, 1.48)	0.47 (0.33, 0.61)
Genders	Male	215.53 (187.96, 229.36)	552.83 (488.69, 665.21)	156.5	0.92 (0.85, 0.98)	1.52 (1.34, 1.83)	0.43 (0.27, 0.59)
	Female	189.85 (175.20, 204.20)	481.84 (427.87, 552.37)	153.8	1.09 (1, 1.14)	1.1 (0.98, 1.27)	0.48 (0.36, 0.6)
SDI quintiles	High SDI	236.29 (216.67, 252.14)	443.87 (384.09, 545.65)	87.85	2.24 (2.05, 2.39)	2.29 (1.99, 2.82)	-0.21 (-0.48, 0.05)
	High-middle SDI	102.93 (92.68, 112.12)	306.56 (276.18, 343.40)	197.83	0.97 (0.88, 1.06)	1.53 (1.38, 1.71)	1.7 (1.5, 1.89)
	Middle SDI	31.21 (26.08, 36.15)	154.59 (135.14, 179.99)	395.31	0.3 (0.25, 0.34)	0.62 (0.54, 0.72)	2.98 (2.76, 3.19)
	Low-middle SDI	23.75 (19.78, 28.18)	82.58 (71.86, 95.17)	247.79	0.42 (0.35, 0.5)	0.62 (0.54, 0.71)	1.21 (1.04, 1.38)
	Low SDI	11.02 (8.74, 13.48)	32.86 (27.61, 38.60)	198.32	0.51 (0.4, 0.63)	0.69 (0.58, 0.82)	1.17 (1.1, 1.25)
GBD Regions	East Asia	20.22 (15.23, 26.47)	162.14 (133.32, 199.11)	701.77	0.2 (0.15, 0.26)	0.81 (0.67, 0.99)	5.84 (5.41, 6.26)
	Southeast Asia	4.31 (3.57, 5.25)	19.17 (15.35, 24.23)	345.27	0.18 (0.15, 0.23)	0.34 (0.27, 0.43)	2.14 (2.09, 2.19)
	Oceania	0.02 (0.02, 0.03)	0.05 (0.04, 0.07)	136.23	0.06 (0.05, 0.08)	0.06 (0.05, 0.08)	-0.35 (-0.46, -0.24)
	Central Asia	2.32 (1.94, 2.61)	4.94 (4.12, 6.00)	113.23	0.46 (0.39, 0.52)	0.65 (0.54, 0.79)	1.32 (1.21, 1.43)
	Central Europe	17.30 (15.70, 20.20)	64.36 (54.75, 78.94)	272.09	1.17 (1.06, 1.37)	3 (2.53, 3.68)	3.77 (3.29, 4.24)
	Eastern Europe	37.58 (30.60, 44.07)	74.24 (65.51, 84.31)	97.88	1.32 (1.07, 1.55)	2.16 (1.9, 2.45)	1.97 (1.76, 2.17)
	High-income Asia Pacific	4.58 (4.25, 5.54)	12.67 (10.38, 15.68)	176.53	0.23 (0.21, 0.28)	0.31 (0.26, 0.38)	1.2 (0.98, 1.43)
	Australasia	6.06 (5.50, 7.02)	15.18 (11.84, 19.98)	150.5	2.55 (2.32, 2.95)	2.99 (2.33, 3.93)	0.22 (-0.05, 0.48)
	Western Europe	134.28 (123.30, 142.03)	275.60 (235.01, 338.18)	105.24	2.3 (2.12, 2.43)	2.96 (2.52, 3.64)	0.7 (0.34, 1.07)
	Southern Latin America	2.73 (2.37, 3.07)	6.10 (4.77, 7.89)	123.15	0.6 (0.52, 0.67)	0.72 (0.56, 0.94)	0.21 (-0.11, 0.53)
	High-income North America	122.10 (110.16, 129.00)	207.23 (174.82, 255.27)	69.73	3.41 (3.06, 3.6)	3.19 (2.69, 3.94)	-0.68 (-0.96, -0.41)
	Caribbean	1.48 (1.32, 1.63)	3.73 (3.09, 4.52)	152.97	0.57 (0.51, 0.63)	0.72 (0.6, 0.87)	0.85 (0.77, 0.92)
	Andean Latin America	0.35 (0.29, 0.44)	1.99 (1.54, 2.49)	468.49	0.16 (0.14, 0.2)	0.35 (0.27, 0.44)	3.05 (2.88, 3.22)
	Central Latin America	2.27 (2.03, 2.41)	9.59 (7.94, 11.77)	322.32	0.28 (0.25, 0.3)	0.41 (0.34, 0.51)	1.22 (1.08, 1.37)
	Tropical Latin America	3.19 (2.93, 3.41)	12.64 (11.13, 14.69)	296.75	0.37 (0.34, 0.4)	0.53 (0.47, 0.62)	1.36 (1.24, 1.47)
	North Africa and Middle East	6.94 (5.50, 8.51)	31.52 (26.99, 37.75)	354.25	0.4 (0.32, 0.49)	0.74 (0.63, 0.9)	2.22 (2.12, 2.31)
	South Asia	26.94 (22.35, 32.58)	97.47 (82.93, 113. 40)	261.79	0.54 (0.45, 0.66)	0.73 (0.62, 0.85)	0.8 (0.63, 0.96)
	Central Sub-Saharan Africa	0.60 (0.41, 0.91)	2.94.32 (1.97, 4.26)	386.88	0.29 (0.2, 0.42)	0.62 (0.41, 0.9)	2.97 (2.67, 3.27)
	Eastern Sub-Saharan Africa	4.15 (3.18, 5.22)	12.70 (10.31, 15.83)	205.91	0.63 (0.49, 0.8)	0.91 (0.73, 1.14)	1.37 (1.29, 1.44)
	Southern Sub-Saharan Africa	3.78 (3.20, 4.34)	9.19 (7.91, 10.41)	143.29	1.4 (1.17, 1.62)	1.7 (1.44, 1.91)	0.85 (0.64, 1.06)
	Western Sub-Saharan Africa	4.25 (3.47, 4.98)	11.23 (9.00, 13.34)	164.32	0.51 (0.41, 0.59)	0.65 (0.52, 0.76)	1 (0.93, 1.07)

ASIR, age-standardized incidence rate; EAPCs, estimated annual percentage changes; SDI, socio-demographic index; CI, confidence interval; UI, uncertainty interval.

### The Mortality of CLL and Its Trend

At the global level, death number of CLL was 446.13 ×10^2^ [95% uncertainty interval (UI) = (403.93, 500.74)] in 2019, which increased 107.03% from 1990 (**Table 2**, **Figures 1D–F** and **Supplementary Table S2**). The age-standardized death rate (ASDR) was 1.28 (1.16, 1.48) per 100,000, decreasing with an annual average of 0.34% [EAPC = −0.34; 95% CI = (−0.43, −0.25)] from 1990 to 2019 (**Table 2**, **Figures 2D–F** and **Figure 3** middle). Among different SDI quintiles, the high SDI quintile always had the highest ASDR from 1990 (0.94/100,000 persons) to 2019 (0.72/100,000 persons), although it kept declining constantly [EAPC = −1.17 (−1.32, −1.02)]. The ASDR trend of the high-middle SDI quintile was a parabola, peaking at 0.67 (0.62, 0.73)/100,000 persons in 2005. Three lower SDI quintiles had increasing trends of ASDRs (**Table 2**, **Figures 2D–F** and **Supplementary Table S5**). Southern Sub-Saharan Africa (1.43 (1.17, 1.6) per 100,000), Central Europe (1.18 (1, 1.42) per 100,000), and High-income North America (0.98 (0.87, 1.19) per 100,000) ranked the top 3 on the list of ASDRs of 21 geographical regions in 2019. The trends of ASDRs from 1990 to 2019 of different geographical regions were quite varied: Central Sub-Saharan Africa had the highest increasing speed [EAPC = 2.8 (2.49, 3.12)], while High-income North America had the most significant decreasing trend [−1.1 (−1.26, −0.94)] (**Table 2** and **Figure 3** middle). On the scale of countries and territories, India (61.96 (50.77, 74.94) ×10^2^), the United States of America (59.42 (52.53, 72.18) ×10^2^), and China (47.12 (38.86, 58.22) ×10^2^) had the largest numbers of death cases in 2019 (**Supplementary Table S5**). The top 3 countries and territories on the ranking of the highest ASDRs of CLL in 2019 were Qatar (3.87 (2.44, 5.84) per 100,000), Seychelles (2.53 (2.12, 3.03) per 100,000), and Lesotho (1.79 (1.17, 2.5) per 100,000) (**Figure 4B** and **Supplementary Table S8**). From 1990 to 2019, Jamaica [EAPC = 6.68 (5.76, 7.6)] had the most significant increasing trend of ASDRs while Netherlands [EAPC = −4.85 (−6.11, −3.57)] dropped the fastest (**Figure 5B** and **Supplementary Table S11**).

**Table 2:** The death cases and ASDRs of CLL in 1990 and 2019, and the trends from 1990 to 2019.

		Death cases (×10^2^, 95% UI)			ASDRs per 100,000 (95% UI)		
		1990	2019	Change Percentages of cases (%)	1990	2019	EAPCs of ASDRs (95% CI)
Global		215.48 (198.11, 230.27)	446.13 (403.93, 500.74)	107.03	1.09 (1, 1.14)	1.28 (1.16, 1.48)	-0.34 (-0.43, -0.25)
Genders	Male	110.64 (96.41, 119.16)	223.06 (199.88, 263.45)	101.61	1.31 (1.15, 1.4)	1.52 (1.34, 1.83)	-0.46 (-0.56, -0.35)
	Female	104.84 (94.93, 115.07)	223.06 (198.58, 250.84)	112.76	0.92 (0.85, 0.98)	1.1 (0.98, 1.27)	-0.23 (-0.31, -0.16)
SDI quintiles	High SDI	99.73 (90.31, 106.73)	15,312 (134.33, 185.62)	53.54	0.94 (0.85, 1.01)	0.72 (0.63, 0.87)	-1.17 (-1.32, -1.02)
	High-middle SDI	57.98 (52.16, 63.41)	118.76 (107.42, 131.33)	104.84	0.58 (0.52, 0.63)	0.6 (0.54, 0.66)	0.12 (-0.08, 0.33)
	Middle SDI	24.93 (20.97, 28.39)	79.10 (69.96, 91.07)	217.25	0.26 (0.22, 0.29)	0.34 (0.3, 0.4)	1.06 (0.98, 1.13)
	Low-middle SDI	22.01 (18.28, 26.25)	65.06 (56.04, 76.12)	195.61	0.42 (0.35, 0.5)	0.52 (0.44, 0.6)	0.59 (0.46, 0.73)
	Low SDI	10.73 (8.47, 13.10)	29.87 (24.81, 35.20)	178.31	0.53 (0.41, 0.64)	0.67 (0.55, 0.8)	0.85 (0.81, 0.9)
GBD Regions	East Asia	14.26 (10.86, 18.72)	48.41 (40.03, 59.74)	239.60	0.15 (0.12, 0.2)	0.25 (0.21, 0.3)	2.24 (2.02, 2.46)
	Southeast Asia	3.80 (3.17, 4.61)	12.98 (10.59, 16.34)	241.26	0.18 (0.15, 0.22)	0.25 (0.2, 0.32)	1.2 (1.13, 1.27)
	Oceania	0.02 (0.01, 0.02)	0.04 (0.03, 0.05)	118.18	0.05 (0.04, 0.07)	0.05 (0.04, 0.07)	-0.6 (-0.7, -0.5)
	Central Asia	1.55 (1.31, 1.75)	2.57 (2.14, 3.12)	65.59	0.32 (0.27, 0.37)	0.37 (0.31, 0.44)	0.56 (0.47, 0.64)
	Central Europe	10.42 (9.46, 12.72)	26.43 (22.34, 31.60)	153.58	0.72 (0.66, 0.88)	1.18 (1, 1.42)	2.12 (1.69, 2.54)
	Eastern Europe	22.15 (18.18, 25.83)	31.47 (27.70, 35.34)	42.08	0.79 (0.65, 0.92)	0.9 (0.79, 1.01)	0.42 (0.22, 0.61)
	High-income Asia Pacific	1.96 (1.81, 2.32)	4.19 (3.44, 5.37)	113.85	0.1 (0.09, 0.12)	0.08 (0.07, 0.1)	-0.75 (-0.84, -0.66)
	Australasia	2.53 (2.27, 2.90)	4.97 (4.16, 6.19)	96.80	1.09 (0.98, 1.25)	0.91 (0.77, 1.13)	-0.92 (-1.07, -0.76)
	Western Europe	63.00 (57.08, 66.86)	100.43 (86.91, 119.83)	59.40	1.06 (0.96, 1.12)	0.93 (0.82, 1.12)	-0.57 (-0.74, -0.4)
	Southern Latin America	2.19 (1.91, 2.46)	3.78 (3.31, 4.51)	72.49	0.5 (0.43, 0.56)	0.44 (0.39, 0.52)	-0.8 (-1.09, -0.52)
	High-income North America	44.69 (40.00, 47.37)	66.96 (59.17, 81.04)	49.84	1.22 (1.09, 1.29)	0.98 (0.87, 1.19)	-1.1 (-1.26, -0.94)
	Caribbean	1.05 (0.93, 1.16)	2.09 (1.75, 2.46)	98.14	0.42 (0.37, 0.47)	0.4 (0.34, 0.48)	-0.11 (-0.25, 0.04)
	Andean Latin America	0.31 (0.26, 0.39)	1.31 (1.02, 1.62)	321.66	0.15 (0.13, 0.19)	0.24 (0.18, 0.29)	1.88 (1.72, 2.04)
	Central Latin America	1.86 (1.66, 1.99)	5. 93 (4.91, 7.21)	218.92	0.24 (0.21, 0.26)	0.26 (0.22, 0.32)	0.16 (0.05, 0.26)
	Tropical Latin America	2.68 (2.46, 2.87)	8.62 (7.51, 10.10)	221.65	0.33 (0.3, 0.36)	0.37 (0.32, 0.44)	0.54 (0.46, 0.62)
	North Africa and Middle East	5.58 (4.41, 6.85)	15.70 (13.32, 18.90)	181.03	0.35 (0.28, 0.43)	0.41 (0.34, 0.5)	0.55 (0.48, 0.63)
	South Asia	25.35 (20.86, 30.63)	78.76 (66.31, 93.69)	210.62	0.55 (0.45, 0.68)	0.63 (0.53, 0.75)	0.18 (0.03, 0.33)
	Central Sub-Saharan Africa	0.58 (0.40, 0.85)	2.66.21 (1.79, 3.88)	359.22	0.3 (0.21, 0.43)	0.61 (0.41, 0.88)	2.8 (2.49, 3.12)
	Eastern Sub-Saharan Africa	4.13 (3.17, 5.21)	11.89 (9.58, 14.73)	187.80	0.67 (0.52, 0.85)	0.92 (0.73, 1.14)	1.2 (1.14, 1.26)
	Southern Sub-Saharan Africa	3.25 (2.77, 3.77)	7.15 (5.97, 8.06)	119.73	1.28 (1.04, 1.51)	1.43 (1.17, 1.6)	0.53 (0.26, 0.81)
	Western Sub-Saharan Africa	4.12 (3.41, 4.81)	9.82 (7.89, 11.58)	138.52	0.5 (0.42, 0.59)	0.59 (0.48, 0.69)	0.68 (0.63, 0.72)

ASDR, age-standardized death rate; EAPCs, estimated annual percentage changes; SDI, socio-demographic index; CI, confidence interval; UI, uncertainty interval.

### The DALY Burden of CLL and Its Trend

The DALYs doubled from 4,920.74 (4,452.50, 5,322.84) years in 1990 to 9,484.64 (8,741.97, 10,652.54) years in 2019 (**Table 3**, **Figures 1G–I** and **Supplementary Table S3**). From 1990 to 2019, the age-standardized DALY rates of CLL decreased significantly [EAPC = −0.34 (−0.4, −0.27)] (**Table 3** and **Figure 3** lower). At the SDI quintiles level, the trends of age-standardized DALY rates were in accordance with the changes of death rates: decreasing in the high SDI; increasing from 1990, peaking at 2005, then declining until 2019 in high-middle SDI quintiles; and increasing in the other three SDI quintiles from 1990 to 2019 (**Figures 2G–I** and **Supplementary Table S6**). Interestingly, the high SDI quintile had the highest age-standardized DALY rate in 1990, but in 2019, the low SDI quintile had the highest value. The high SDI quintile had a considerable decrease in age-standardized DALY rates with EAPC at −1.32 (−1.5, −1.14) (**Table 3** and **Supplementary Table S6**, **Figure 2G**, **Figure 3** lower). In terms of geographical regions, Southern Sub-Saharan Africa (29.73 (25.33, 33.8) per 100,000) and Central Europe (25.09 (21.23, 30.39) per 100,000) were the top two regions with the highest DALY rates in 2019. The EAPCs of age-standardized DALYs rates varied in different geographical regions: from the highest [Central Sub-Saharan Africa, 2.66 (2.34, 2.98)] to lowest [High-income North America, −1.48 (−1.66, −1.29)] (**Table 3** and **Figure 3** lower). Regarding observation of countries and territories, in 2019, China (1,469.13 (1,218.16, 1,792.32) ×10^2^) and India (1,397.69 (1,142.67, 1,707.53) ×10^2^) had the most DALYs (**Supplementary Table S6**). Qatar (54.89 (38.8, 78.2)/100,000 persons), Seychelles (54.31 (45.79, 64.76)/100,000 persons), and Lesotho (38.06 (24.43, 53.76)/100,000 persons) had the highest age-standardized DALY rates in 2019 (**Figure 4C** and **Supplementary Table S9**). Jamaica [EAPC = 6.72 (5.81, 7.63)] had the most increase in age-standardized DALY rates, while Netherlands [EAPC = −4.82 (−6.06, −3.55)] had the most decrease (**Figure 5C** and **Supplementary Table S12**).

**Table 3:** The DALYs and age-standardized DALY Rates of CLL in 1990 and 2013, and the trends from 1990 to 2019.

		DALYs (×10^2^, 95% UI)			Age-Standardized DALY Rates per 100,000 (95% UI)		
		1990	2019	Change Percentages of DALYs (%)	1990	2019	EAPCs of Age-Standardized DALY Rates (95% CI)
Global		4,920.74 (4,452.50, 5,322.84)	9,484.64 (8,741.97, 10,652.54)	92.75	12.5 (11.41, 13.48)	11.65 (10.72, 13.1)	-0.34 (-0.4, -0.27)
Genders	Male	2,620.91 (2,263.04, 2,864.75)	4,858.99 (4,363.26, 5,723.79)	85.39	14.8 (12.86, 16.02)	13.01 (11.68, 15.29)	-0.49 (-0.59, -0.39)
	Female	2,299.84 (2,055.07, 2,577.62)	4,625.65 (4,120.79, 5,275.03)	101.13	10.77 (9.61, 12.03)	10.63 (9.47, 12.13)	-0.19 (-0.26, -0.12)
SDI Quintiles	High SDI	1,944.55 (1,776.26, 2,089.29)	2,632.63 (2,353.15, 3,229.85)	35.39	18.53 (16.9, 19.96)	13.64 (12.28, 16.72)	-1.32 (-1.5, -1.14)
	High-middle SDI	1,402.98 (1,248.66, 1,554.35)	2,545.13 (2,341.45, 2,840.04)	81.41	12.9 (11.5, 14.3)	12.71 (11.69, 14.15)	-0.08 (-0.24, 0.08)
	Middle SDI	734.69 (597.50, 867.40)	2,033.96 (1,805.27, 2,342.81)	176.84	6.26 (5.22, 7.22)	8.04 (7.12, 9.26)	1.02 (0.91, 1.13)
	Low-middle SDI	569.47 (479.14, 679.76)	1,555.53 (1,345.94, 1,816.49)	173.15	9.17 (7.66, 10.92)	11.2 (9.67, 13.08)	0.57 (0.43, 0.71)
	Low SDI	266.93 (210.66, 325.61)	713.44 (597.46, 834.46)	167.28	11.24 (8.89, 13.74)	13.88 (11.55, 16.31)	0.75 (0.7, 0.79)
GBD Regions	East Asia	520.51 (383.84, 700.76)	1,501.93 (1,249.41, 1,826.59)	188.55	4.7 (3.51, 6.24)	7.75 (6.44, 9.4)	2.19 (1.95, 2.43)
	Southeast Asia	95.06 (79.00, 116.84)	290.03 (238.18, 364.78)	205.1	3.61 (3.01, 4.37)	4.87 (3.99, 6.11)	1.01 (0.95, 1.07)
	Oceania	0.55 (0.40, 0.73)	1.21 (0.86, 1.70)	121.32	1.45 (1.08, 1.93)	1.36 (0.98, 1.86)	-0.44 (-0.51, -0.36)
	Central Asia	48.80 (40.69, 55.21)	72.53 (60.94, 88.87)	48.63	9.36 (7.82, 10.62)	9.17 (7.69, 11.13)	-0.17 (-0.25, -0.09)
	Central Europe	237.03 (213.69, 284.28)	533.43 (453.28, 643.32)	125.05	15.98 (14.42, 18.91)	25.09 (21.23, 30.39)	1.96 (1.53, 2.38)
	Eastern Europe	572.17 (457.99, 677.76)	734.59 (642.27, 832.19)	28.39	20.13 (16.1, 23.97)	21.56 (18.85, 24.43)	0.1 (-0.1, 0.3)
	High-income Asia Pacific	44.68 (41.66, 54.63)	75.89 (64.80, 93.91)	69.86	2.24 (2.09, 2.73)	1.86 (1.63, 2.21)	-0.61 (-0.73, -0.5)
	Australasia	48.97 (44.43, 57.37)	85.41 (72.99, 106.85)	74.4	20.68 (18.76, 24.18)	16.77 (14.37, 21.09)	-1.05 (-1.24, -0.86)
	Western Europe	1,201.95 (1,103.38, 1,289.05)	1,615.22 (1,433.69, 1,939.40)	34.38	20.77 (19.08, 22.32)	17.13 (15.32, 20.79)	-0.86 (-1.08, -0.63)
	Southern Latin America	44.39 (38.59, 50.01)	67.59 (59.68, 80.02)	52.28	9.63 (8.37, 10.86)	8.05 (7.12, 9.5)	-1.03 (-1.31, -0.74)
	High-income North America	882.94 (793.23, 938.53)	1,181.33 (1,059.13, 1,444.17)	33.8	24.98 (22.45, 26.54)	18.38 (16.56, 22.54)	-1.48 (-1.66, -1.29)
	Caribbean	23.04 (20.49, 25.55)	43.37 (36.26, 51.76)	88.27	8.72 (7.79, 9.65)	8.4 (7.02, 10)	-0.14 (-0.25, -0.03)
	Andean Latin America	8.65 (7.05, 10.96)	30.46 (23.15, 38.63)	251.98	3.68 (3.04, 4.62)	5.3 (4.06, 6.68)	1.5 (1.34, 1.65)
	Central Latin America	45.64 (40.66, 48.47)	126.43 (104.93, 155.05)	176.98	5.12 (4.58, 5.44)	5.36 (4.46, 6.57)	0.03 (-0.07, 0.12)
	Tropical Latin America	61.89 (56.85, 66.36)	169.91 (152.08, 199.02)	174.56	6.79 (6.24, 7.27)	7.1 (6.35, 8.32)	0.24 (0.17, 0.32)
	North Africa and Middle East	154.10 (118.40, 191.88)	393.18 (329.89, 465.10)	155.14	8.15 (6.38, 10.08)	8.82 (7.5, 10.48)	0.19 (0.12, 0.26)
	South Asia	638.87 (529.44, 766.80)	1,826.08 (1,549.90, 2,178.30)	185.83	11.4 (9.38, 13.73)	13.01 (11.03, 15.5)	0.25 (0.1, 0.4)
	Central Sub-Saharan Africa	15.34 (10.57, 23.11)	68.29 (45.38, 99.00)	345.21	6.5 (4.51, 9.57)	12.68 (8.5, 18.45)	2.66 (2.34, 2.98)
	Eastern Sub-Saharan Africa	97.46 (74.45, 124.07)	265.68 (216.60, 332.14)	172.61	13.5 (10.35, 17.06)	17.46 (14.2, 21.77)	0.99 (0.93, 1.05)
	Southern Sub-Saharan Africa	84.00 (72.20, 95.27)	170.79 (146.70, 196.15)	103.32	28.73 (24.35, 32.85)	29.73 (25.33, 33.8)	0.38 (0.13, 0.64)
	Western Sub-Saharan Africa	94.70 (78.04, 111.70)	231.27 (185.09, 277.07)	144.21	10.78 (8.92, 12.65)	12.63 (10.16, 15)	0.68 (0.63, 0.73)

DALY, disability-adjusted life year; EAPCs, estimated annual percentage changes; SDI, socio-demographic index; CI, confidence interval; UI, uncertainty interval.

### The Age and Gender Distribution of Incidence, Mortality, and DALYs

**Figure 6:**
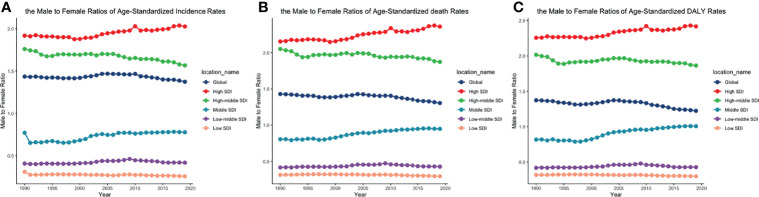
The male-to-female ratios of **(A)** ASIR, **(B)** ASDRs, and **(C)** age-standardized DALY rates by SDI quintiles from 1990 to 2019. (ASIR, age-standardized incidence rate; ASDR, age-standardized death rate; DALY, disability-adjusted life year; SDI, socio-demographic index).

**Figure 7:**
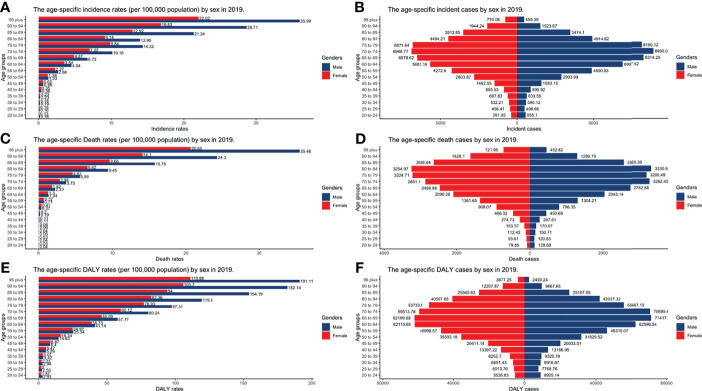
The burden of incidence, deaths, and DALY by gender and age groups in 2019. **(A)** Incident cases; **(B)** Incidence rates; **(C)** death cases; **(D)** death rates; **(E)** DALY rates; **(F)** DALY cases (DALY, disability-adjusted life year).

The trends of age-standardized incidence rates, death rates, and DALY rates of women or men were consistent with those of both genders, both globally and in the scale of SDI quintiles (**Figures 2A–H** and **Supplementary Tables S4**–**S6**). The male-to-female ratios of ASIRs were more than one and increasing from the age group of 55 to 59 significantly, which showed that men were more susceptive to CLL especially in elder people, while male-to-female ratios of incident cases were decreasing from the age group of 70 to 74 probably due to women having a higher average life expectancy than men (**Figures 7A, B** and **Supplementary Table S13**). The ASDRs and the age-standardized DALY rates had a similar trend compared with ASIRs (**Figures 7C–F** and **Supplementary Tables S14**, **S15**). The male-to-female ratios of ASIRs, ASDRs, and age-standardized DALY rates were varied in different SDI quintiles: men had higher burdens of incidence, death, and DALY in high and high-middle SDI quintiles (male-to-female ratio ≥ 1), while the other three lower SDI quintiles had the opposite trend (male-to-female ratio < 1) (**Figures 2B, C, E, F, H, I** and **Figures 6A–C**).

**Figure 8:**
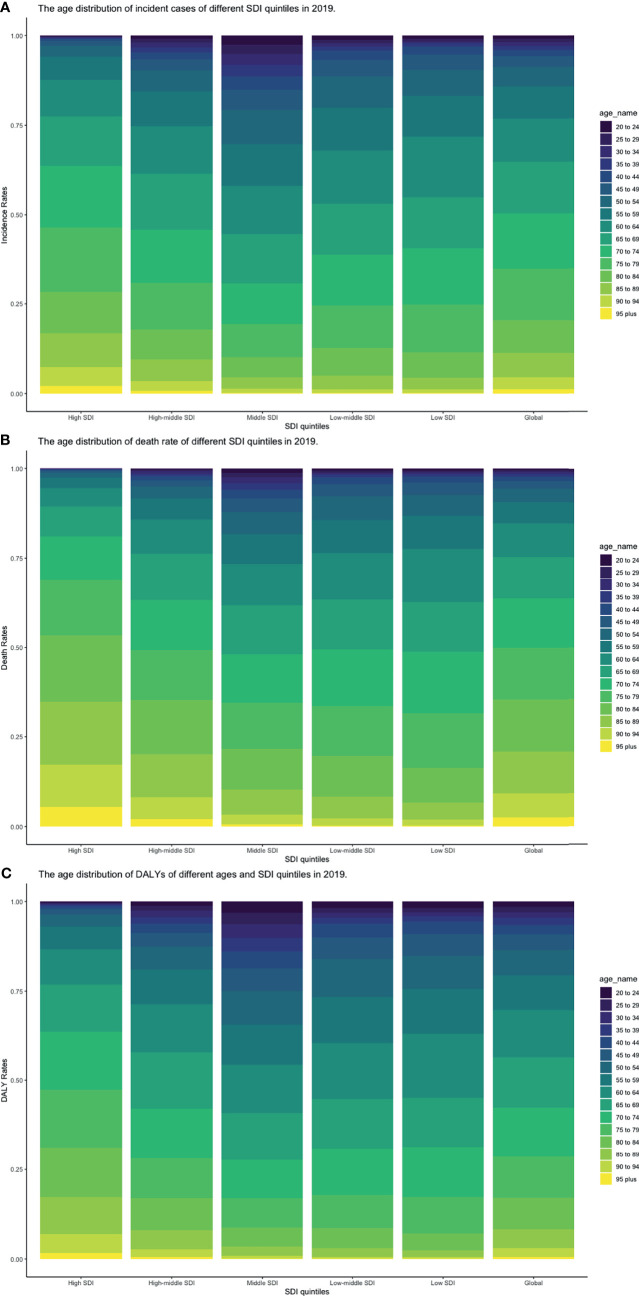
Proportion of age groups on **(A)** incident cases, **(B)** death cases, and **(C)** DALYs by SDI quintiles in 2019 (DALY, disability-adjusted life year; SDI, socio-demographic index).

**Figure 9:**
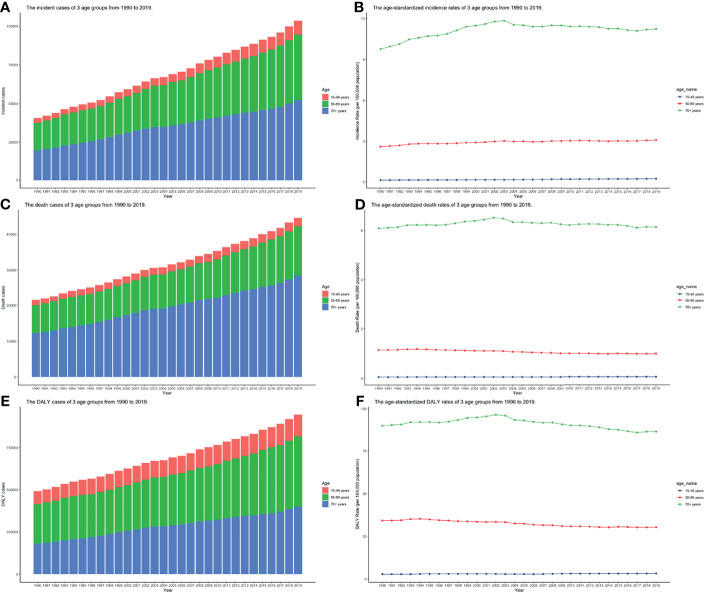
The burden of incidence, deaths, and DALY of 3 age groups from 1990 to 2019. **(A)** Incident cases; **(B)** Incidence rates; **(C)** death cases; **(D)** death rates; **(E)** DALY rates; **(F)** DALYs (ASIR, age-standardized incidence rate; ASDR, age-standardized death rate; DALY, disability-adjusted life year).

CLL occurred almost exclusively after the age of 20 years, and the incidence rates, death rates, and DALY rates increased significantly with age, in both men and women (**Figures 7A, C, E** and **Supplementary Tables S13**-S**15**). People from 60 to 75 had the most incident cases and longest DALYs, and people under the age of 70–85 suffered from the most death cases (**Figures 7B, D, F** and **Supplementary Tables S13**-S**15**). The higher the SDI, the higher proportions of the elderly cases (**Figures 8A–C** and **Supplementary Tables S16**-S**18**) were. The incident case, death cases, and DALYs of different age groups all increased with years, but ASIRs, ASDRs, and age-standardized DALY rates changed slightly (**Figures 9A–F**).

### The Correlation Between SDI and CLLL’s Incidence, Mortality, and DALYs

**Figure 10:**
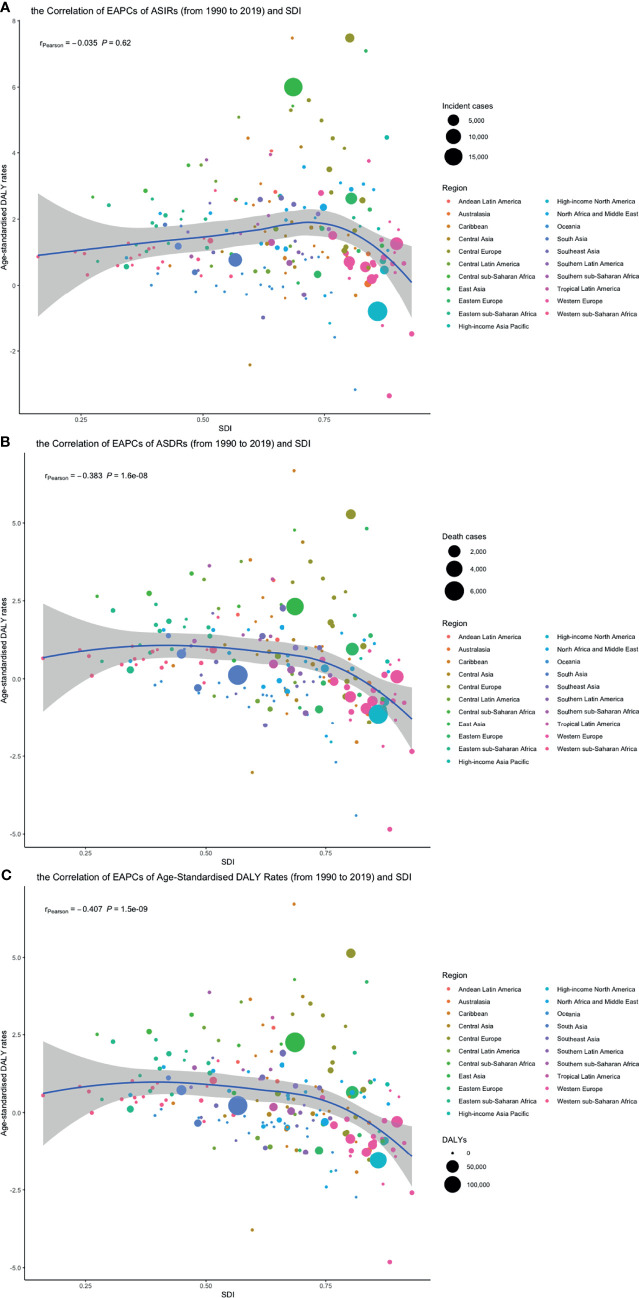
The correlation between EAPCs of **(A)** ASIR, **(B)** ASDRs, and **(C)** age-standardized DALY rates from 1990 to 2019 and SDI in 2019. The circles represent 204 countries or territories, the size of circle represents the number of incident cases, death cases or DALY, and the color of circle represents the region which the country or territory belongs to. (ASIR, age-standardized incidence rate; ASDR, age-standardized death rate; DALY, disability-adjusted life year; SDI, socio-demographic index; EAPC, estimated annual percentage change, SDI, socio-demographic index).

**Figure 11:**
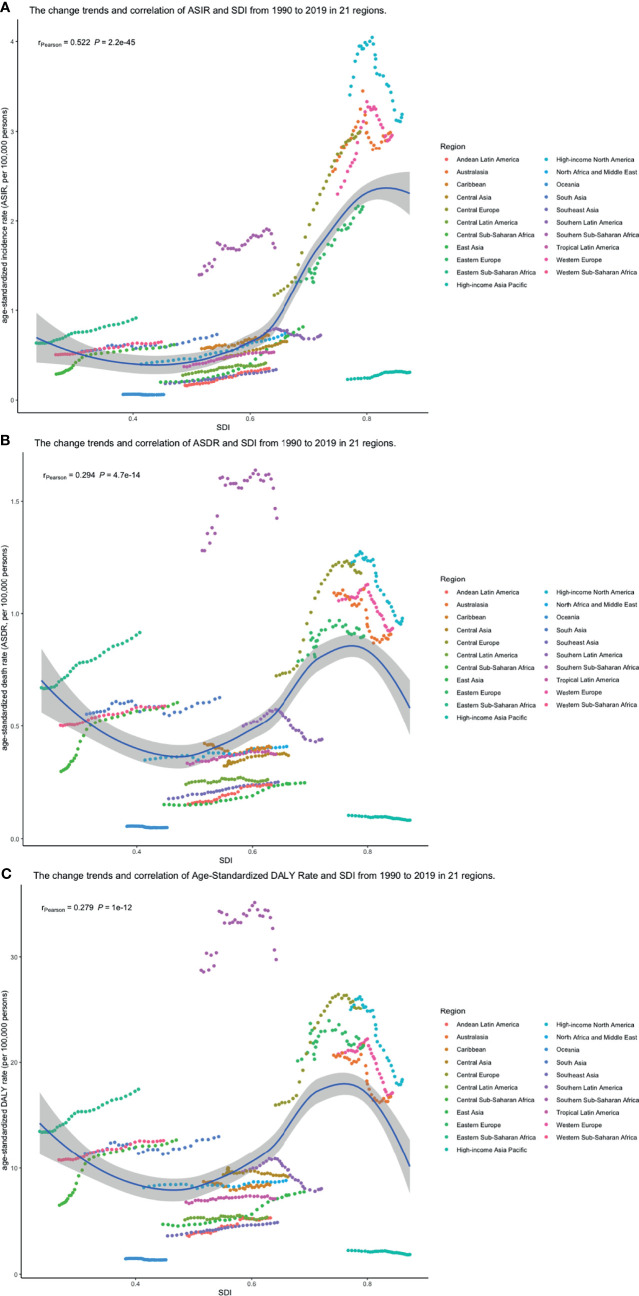
The change trend and correlation between **(A)** ASIR, **(B)** ASDRs, and **(C)** age-standardized DALY rates and SDI from 1990 to 2019. The color of circle represents the 21 different regions. (ASIR, age-standardized incidence rate; ASDR, age-standardized death rate; DALY, disability-adjusted life year; SDI, socio-demographic index; SDI, socio-demographic index).

Pearson correlation analysis did not show the correlation between the EAPCs of ASIRs (*r* = 0.035, *p* = 0.62), ASDRs (*r* = −0.383, *p* < 0.01), and age-standardized DALY rates (*r* = −0.407, *p* < 0.01) from 1990 to 2019 and SDI in 2019. Then, we used loess local weighted regression (LOESS) to fit the correlation between the EAPCs of ASIRs, ASDRs, age-standardized DALY rates from 1990 to 2019, and SDI in 2019. We found that the EAPCs of ASIRs were stable when SDI was lower than 0.65, but increased when SDI was higher, which indicated that the incidence might increase more rapidly in higher SDI countries (**Figure 10A**). The EAPCs of ASDRs and age-standardized DALY rates were stable when SDI was lower than 0.75, too. However, they decreased significantly when SDI was higher, demonstrating that the burdens of death and DALY were declining in higher SDI countries (**Figures 10B, C**). Then, we investigate the correlation between SDI and ASIRs, ASDRs, and age-standardized DALY rates in 21 regions around the globe by Pearson correlation analysis and LOESS. Although Pearson correlation analysis did not show the correlation between the ASDR (*r* = 0.294, *p* < 0.01), age-standardized DALY rates (*r* = 0.279, *p* < 0.01), and SDI from 1990 to 2019, ASIRs (*r* = 0.522, *p* < 0.01) had a positive correlation with SDI. LOESS results showed that all change trends were “S” shaped, dipping when SDI was about 0.45, and ASIRs kept increasing after that, while ASDRs and age-standardized DALY rates peaked when SDI was about 0.75, then declined (**Figures 11A–C**).

### CLL Burden Attributable to Risk Factors

**Figure 12:**
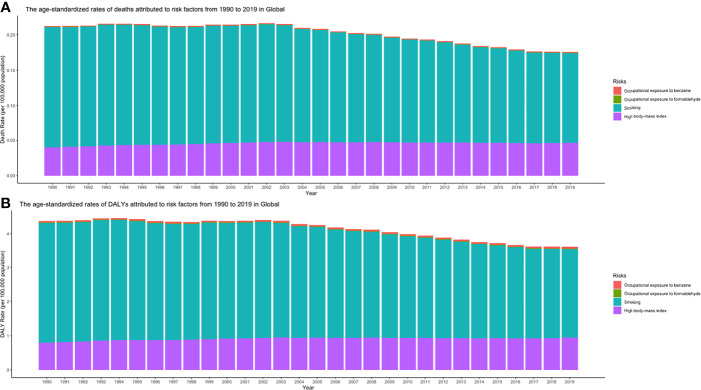
**(A)** ASDRs and **(B)** age-standardized DALY rates attributed to risk factors from 1990 to 2019 in Global (ASDR, age-standardized death rate; DALY, disability-adjusted life year).

From 1990 and 2019, smoking, high body mass index, and occupational exposure to benzene or formaldehyde were the potential risk factors related to CLL burden in the GBD study. Among all risk factors, smoking was the greatest contributor to CLL-related death and DALY from 1990 to 2019 in the globe but dropping gradually (**Figures 12A, B**).

### CLL Incidence and Death Rate Projections Till 2030

**Figure 13:**
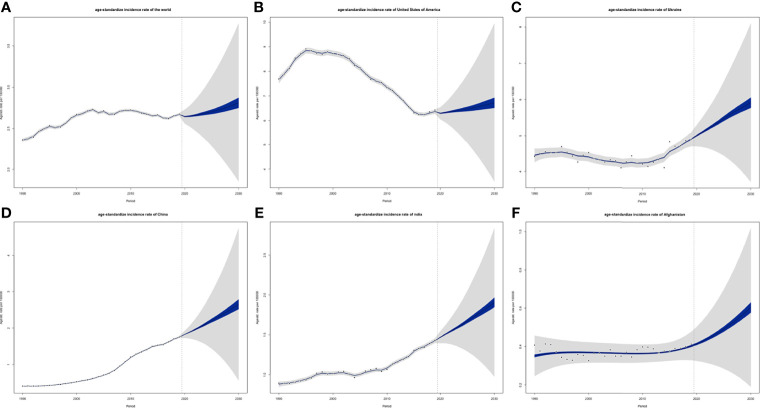
Projections of ASIRs of **(A)** the world, **(B)** USA, **(C)** Ukraine, **(D)** China, **(E)** India and **(F)** Afghanistan from 2020 to 2030: blue areas are 5% confidence intervals and grey areas are 95% confidence intervals; black dots are observation value from 1990 to 2019 (ASIR, age-standardized incidence rate).

**Figure 14:**
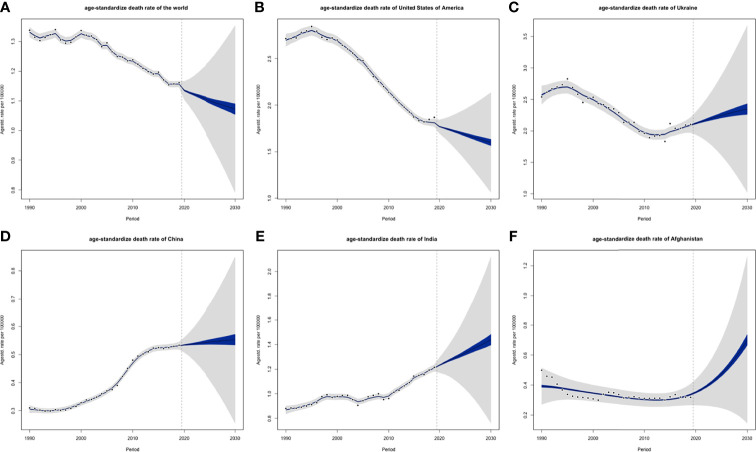
Projections of ASDRs of **(A)** the world, **(B)** USA, **(C)** Ukraine, **(D)** China, **(E)** India and **(F)** Afghanistan from 2020 to 2030: blue areas are 5% confidence intervals and grey areas are 95% confidence intervals; black dots are observation value from 1990 to 2019 (ASDR, age-standardized death rate; DALY).

ASIRs of the world might tend to increase, and so were 5 individual countries that represented 5 different SDI levels, respectively (**Figure 13**). However, their ASDRs were varied: global ASDRs and the ASDRs of the United States (representing the high SDI quintile) might decrease until 2030, and the ASIRs of Ukraine (high-middle SDI quintile), China (middle SDI quintile), India (low-middle SDI quintile), and Afghanistan (low SDI quintile) would increase (**Figure 14**).

## Discussion

In the present study, trends of CLL disease burden were assessed based on the GBD study from 1990 to 2019, which provides valuable epidemiologic information for health promotion and disease prevention. Globally, the ASDRs and age-standardized DALY rates generally declined, yet the ASIRs increased slightly. During the same period, the incident cases, death cases, and DALYs of CLL continued to increase, partly due to population growth in developing countries and population aging in developed countries (33). From 1990 to 2019, the ASIRs showed an increasing trend in most geographical regions except in high-income North America and Oceania. Some middle or high-middle SDI regions such as Eastern Asia and Central Europe experienced the fastest growth. In terms of ASDRs and age-standardized DALY rates, the burden of death and DALY in low SDI quintiles stepped up dramatically. Meanwhile, ASDRs and age-standardized DALY rates of high SDI quintiles, such as high-income North America, Western Europe, and Australasia, dropped obviously.

The occurrence of CLL is strongly genetically determined. Environmental factors may also operate but are likely to be less important (34). In our study, the ASIRs in high SDI regions like North America, Western Europe, and Australasia are about 10 times more than High-income Asia Pacific, which has a similar SDI to them. Some genome-wide association studies (GWAS) may explain this phenomenon: these studies support a hereditary link between disease susceptibility CLL and some single-nucleotide polymorphisms (SNPs), including 10q23.31 [ACTA2 or FAS (ACTA2/FAS), 18q21.33 (BCL2), 11p15.5 (C11orf21), 4q25 (LEF1), 2q33.1 (CASP10 or CASP8), 9p21.3 (CDKN2B-AS1), 18q21.32 (PMAIP1), 15q15.1 (BMF), 2p22.2 (QPCT), 2q13 (ACOXL), 8q22.3 (ODF1) and 5p15.33 (TERT)], several of which are proximal to genes involved in apoptosis (35). GWAS of familial cases of CLL suggests that the 6p21.3 region, which includes HLA-DQA1 and HLA-DRB5, is associated with susceptibility to CLL (36–38). However, ethnic differences are found in SNPs associated with CLL: some SNPs associated with CLL found by GWAS of European population are studied in Chinese of Hong Kong, and it demonstrates that mean allele frequencies (MAF) of some CLL-associated SNPs in Chinese are moderately to extremely lower than those in persons of predominantly European descent, which means that those gene determined in the European population are not significantly associated with susceptibility to CLL in the Chinese population (39). These genetic differences may underlie different etiological, pathogenetic, and biological features of CLL between Asians and persons of predominantly European descent with CLL and explain the regional difference of CLL’s incidence partly. All populations arose from a common African Black ancestor, but different clades have different admixture with archaic hominins including Neanderthals, Denisovans, and *Homo erectus*, which may explain different CLL incidences (5).

Apart from genetic factors, some socioeconomic reasons may also account for the “S”-shaped trend between ASIRs and SDI. Regular blood routine screening may be important in funding asymptomatic patients but unavailable in lower SDI countries: a cross-sectional descriptive study reported 9.1% of patients diagnosed with CLL in Sudan were Rai stage 0 (40), similar percentages were found in Pakistan (41) and Northern India (42), and this percentage in developed countries was 17.6% (43); Binet stage A patients in South-Western Nigeria were 18.2% of all patients diagnosed with CLL (44), while those in developed countries account for 50% (45). The diagnosis of CLL is established by blood counts, blood smears, and immunophenotyping of circulating B lymphocytes to identify a clonal B-cell population (46), which may not be easily accessible in lower SDI countries. In addition, for many developing countries, data sources for informing cancer burden estimation are still sparse (19), and cancer incidence data available in low- and middle-income countries (LMIC) are limited (47); thus, the incidence rates in lower SDI countries may be underestimated.

The pharmaceutic development has changed the landscape of the treatment of CLL, therefore reducing the disease burden of death and DALY in high SDI regions. In the 1970s, oral alkylating agents such as chlorambucil with/without steroids or cyclophosphamide with/without prednisone were the “standard” regimens (48–50). However, the paradigm shifted in the treatment of CLL over the last couple of years. The treatment in CLL has come a long way since the advent of chlorambucil with/without prednisone, purine analogues such as fludarabine, pentostatin, or a combination of fludarabine with cyclophosphamide, and later on combination with rituximab in the late 2000s (51). Moreover, novel targeted drugs for CLL, including Bruton’s tyrosine kinase inhibitors (BTKi; ibrutinib and acalabrutinib), BCL2 inhibitors (venetoclax), and phosphatidylinositol 3-kinase inhibitors (PI3Ki; idelalisib and duvelisib), have improved the prognosis of CLL patients in developed countries significantly in the last 10 years (7). Unfortunately, access to the novel agents is currently limited to certain developed countries, and every effort should be made to make sure patients in developing countries also benefit from these outstanding drugs (12). Most novel drugs and targeted drugs approved by the US Food and Drug Administration (FDA) and marketed in the United States are not available in developing countries such as India (52). In a resource-limited hospital in Nigeria, the major first-line chemotherapy used was cyclophosphamide, vincristine, and prednisolone (CVP) for 42.9% of patients, followed by chlorambucil and prednisolone (CP) for 31.4%. Few patients are accessible to newer drugs, like rituximab with cyclophosphamide, hydroxodaunorubicin, oncovin, and prednisolone (R-CHOP), or fludarabine, cyclophosphamide, and prednisolone (FCP) (53). This situation may partly explain the increasingly serious disease burden of death and DALY in low SDI countries.

CLL occurred almost exclusively after the age of 20 years, and the incidence rates, death rates, and DALY rates increased significantly with age, in both men and women. It poses a serious challenge to aging countries. Unusual low male-to-female ratios of incidence rates (male-to-female ratio < 1) are observed in lower SDI quintiles in our study. Moreover, we found that male-to-female ratios of incidence rates in different age groups are all affected by SDI quintiles (**Supplementary Figures S1A–F**). The low male-to-female ratios are predominant in Africa, South Asia, Greenland, Mongolia, and some countries in Southeast Asia (**Supplementary Figure S2**). Some studies achieved similar results (44, 53), while others did not (40–42). Considering the possible underestimation of incidence rates in lower SDI countries and the male-to-female ratio of CLL in African American patients (male-to-female ratio ≥ 1), we infer that the incidence rates of men were underestimated more badly than those of women. The more significant correlation between incidence rates and SDI observed in men than in women (**Supplementary Figures S3A-C**) may support our inference.

In the current study, smoking, high body mass index, and occupational exposure to benzene or formaldehyde are the potential risk factors related to CLL burden. Several lifestyles and occupational or environmental histories are thought to be risk factors that predispose to CLL/SLL, including farmers (may have benzene and heavy solvent exposure) (54, 55) and central obesity in women (56), but these associations have not been proven in other studies (54).

This study has some limitations. The accuracy of the results depended on the quality and quantity of GBD data (57). However, as data sources for cancer burden estimation were scarce, and cancer registries covered only a small fraction of the population, especially in some underdeveloped regions, the accuracy and integrity of the GBD data were potentially compromised. Besides, misdiagnosed patients might have a potential impact on the incident cases. In addition, the incidence rates of CLL may be underestimated. Furthermore, miscoded incidence/deaths and changes in coding practices or coding systems may also have an effect (19).

## Conclusions

Globally, the incidence of CLL had been increasing from 1990 to 2019, and the burden of death and DALY decreased slightly. The ASIRs showed an increasing trend in most geographical regions except in high-income North America and Oceania. The burden of death and DALY was affected by SDI: in low SDI quintiles, ASDRs and age-standardized DALY rates stepped up dramatically, while ASDRs and age-standardized DALY rates of high SDI quintiles dropped significantly. It might result from imbalanced access to novel agents. It posed a serious challenge to aging countries that the incidence rates, death rates, and DALY rates of CLL increased significantly with age. The incidence rates of men were underestimated compare to those of women from unusually low male-to-female ratios of incidence rates (male-to-female ratio < 1) observed in lower SDI quintiles. Global ASIRs might tend to increase until 2030, while ASDRs of the world would decrease until 2030 possibly because of the dropping ASDRs of high SDI quintile countries. Consequently, strategies for early detection of asymptomatic CLL, development of novel drugs, and measures against attributable factors such as smoking and high body mass index should be implemented to reduce CLL burden, especially in lower SDI quintiles.

## Data Availability Statement

The original contributions presented in the study are included in the article/**Supplementary Material**. Further inquiries can be directed to the corresponding authors.

## Ethics Statement

The study was in accordance with the ethical standards formulated in the Helsinki Declaration and was approved by the respective local Medical Ethics Committees of Jinshan Hospital of Fudan University.

## Author Contributions

YO, YL, and YC conceived the study. YO, YL, YZ, HC, LJ, TQ, XW, and YC performed the literature review, analyzed the data, and drafted the manuscript. HC and XW contributed to the critical revision of the manuscript. All authors contributed to the article and approved the submitted version.

## Funding

This work was supported by grants from Program of the Shanghai Academic/Technology Researcher leader (20XD1401000), Shanghai Engineering Research Center of Tumor Multi-Target Gene Diagnosis (20DZ2254300), and Key Subject Construction Program of Shanghai Health Administrative Authority (ZK2019B30). All authors obtained permission to acknowledge all those mentioned in the Acknowledgments.

## Conflict of Interest

The authors declare that the research was conducted in the absence of any commercial or financial relationships that could be construed as a potential conflict of interest.

## Publisher’s Note

All claims expressed in this article are solely those of the authors and do not necessarily represent those of their affiliated organizations, or those of the publisher, the editors and the reviewers. Any product that may be evaluated in this article, or claim that may be made by its manufacturer, is not guaranteed or endorsed by the publisher.

## Supplementary Material

Supplementary Table S1 Age-standardized incidence rates by genders in different SDI quintiles from 1990 to 2019. SDI, socio-demographic index.

Supplementary Table S2 Age-standardized death rate by gender in different SDI quintiles from 1990 to 2019. SDI, socio-demographic index.

Supplementary Table S3 Age-standardized DALY rates by genders in different SDI quintiles from 1990 to 2019. DALY, disability-adjusted life year; SDI, socio-demographic index.

Supplementary Table S4 Incidence cases of 204 countries and territories in 2019 (from high to low). UI: uncertainty interval.

Supplementary Table S5 Death cases of 204 countries and territories in 2019 (from high to low). UI: uncertainty interval.

Supplementary Table S6 The DALYs 204 countries and territories in 2019 (from high to low). DALY: disability-adjusted life year, UI: uncertainty interval.

Supplementary Table S7 Age-standardized incidence rates of 204 countries and territories in 2019 (from high to low). ASIR: age-standardized incidence rate, UI: uncertainty interval.

Supplementary Table S8 Age-standardized death rates of 204 countries and territories in 2019 (from high to low). ASDR: age-standardized death rate, UI: uncertainty interval.

Supplementary Table S9 Age-standardized DALY rates of 204 countries and territories in 2019 (from high to low). DALY: disability-adjusted life year, UI: uncertainty interval.

Supplementary Table S10 The EAPCs of ASIRs of 204 countries and territories from1990 to 2019 (from high to low). ASIR: age-standardized incidence rate, EAPCs: estimated annual percentage changes, CI: confidence interval.

Supplementary Table S11 The EAPCs of ASDRs of 204 countries and territories from1990 to 2019 (from high to low). ASDR: age-standardized death rate, EAPCs: estimated annual percentage changes, CI: confidence interval.

Supplementary Table S12 The EAPCs of age-standardized DALY Rates of 204 countries and territories from1990 to 2019 (from high to low). DALY: disability-adjusted life year, EAPCs: estimated annual percentage changes, CI: confidence interval.

Supplementary Table S13 The age and gender distribution of incident cases and ASIRs of CLL in 2019. ASIR: age-standardized incidence rate, UI: uncertainty interval.

Supplementary Table S14 The age and gender distribution of death cases and ASIRs of CLL in 2019. ASDR: age-standardized death rate, UI: uncertainty interval.

Supplementary Table S15 The age and gender distribution of DALYs and age-standardized DALY Rates of CLL in 2019. DALY: disability-adjusted life year, UI: uncertainty interval.

Supplementary Table S16 The age and region distribution of incident cases (95% UI) of CLL in 2019. ASIR: age-standardized incidence rate, UI: uncertainty interval.

Supplementary Table S17 The age and region distribution of death cases (95% UI) of CLL in 2019. ASDR: age-standardized death rate, UI: uncertainty interval.

Supplementary Table S18 The age and region distribution of DALYs (95% UI) of CLL in 2019. DALY: disability-adjusted life year, UI: uncertainty interval.
