# Supplementary material for: Trends in Disease Burden of Chronic Lymphocytic Leukemia at the Global, Regional, and National Levels From 1990 to 2019, and Projections Until 2030: A Population-Based Epidemiologic Study
Source: Front Oncol. 2022 Mar 10;12:840616. doi: 10.3389/fonc.2022.840616 (PMC8961301; doi:10.3389/fonc.2022.840616)
Supplement: Supplementary file 1 [file DataSheet_1.pdf]

A. The incidence rates (per 100,000 population) by gender and age groups in high SDI countries.

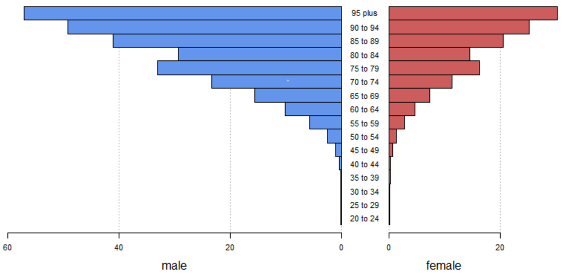

B. The incidence rates (per 100,000 population) by gender and age groups in high-middle SDI countries.

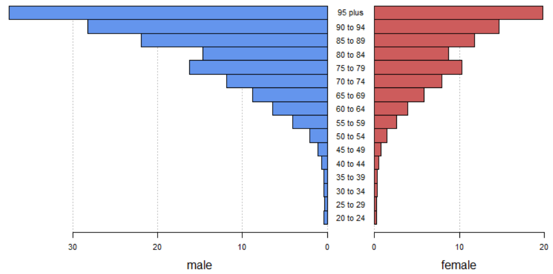

C. The incidence rates (per 100,000 population) by gender and age groups in middle SDI countries.

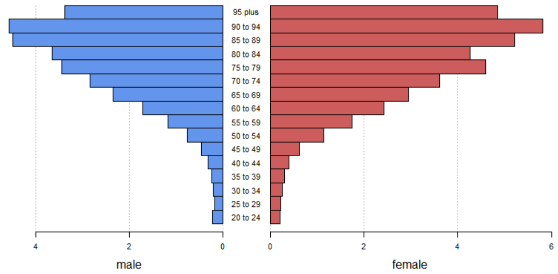

D. The incidence rates (per 100,000 population) by gender and age groups in low-middle SDI countries.

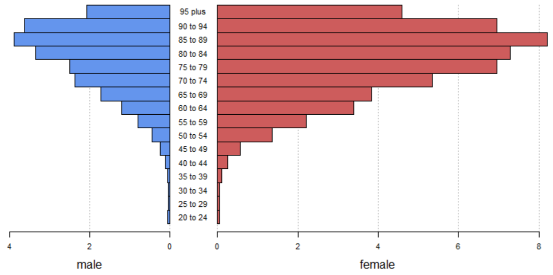

E. The incidence rates (per 100,000 population) by gender and age groups in low SDI countries.

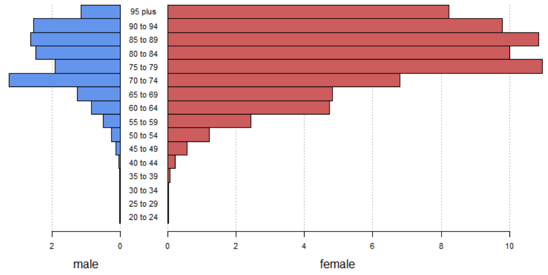

Supplementary Figure S1. The incidence rates of different SDI quintiles by gender and age groups in 2019: (A) The incidence rates of high SDI quintiles, (B) The incidence rates of high middle SDI quintiles, (C) The incidence rates of middle SDI quintiles, (D) The incidence rates of low middle SDI quintiles, (E) The incidence rates of low SDI quintiles (SDI, socio-demographic index).

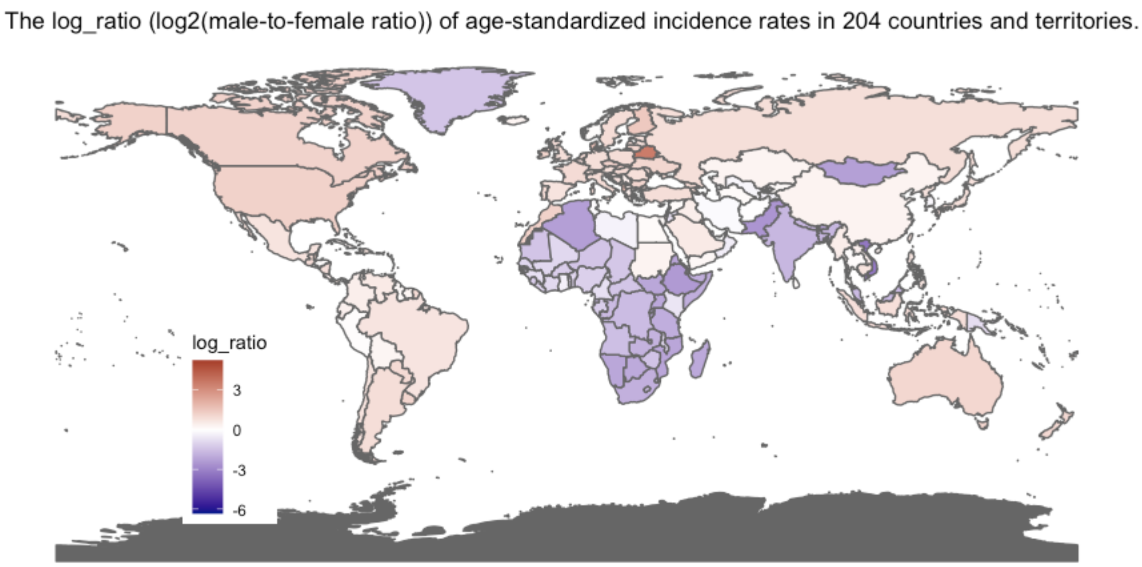

Supplementary Figure S2. Map of the log_ratios (log2(male-to-female ratio)) of ASIRs by country and territory in 2019 (ASIR, age-standardized incidence rate).

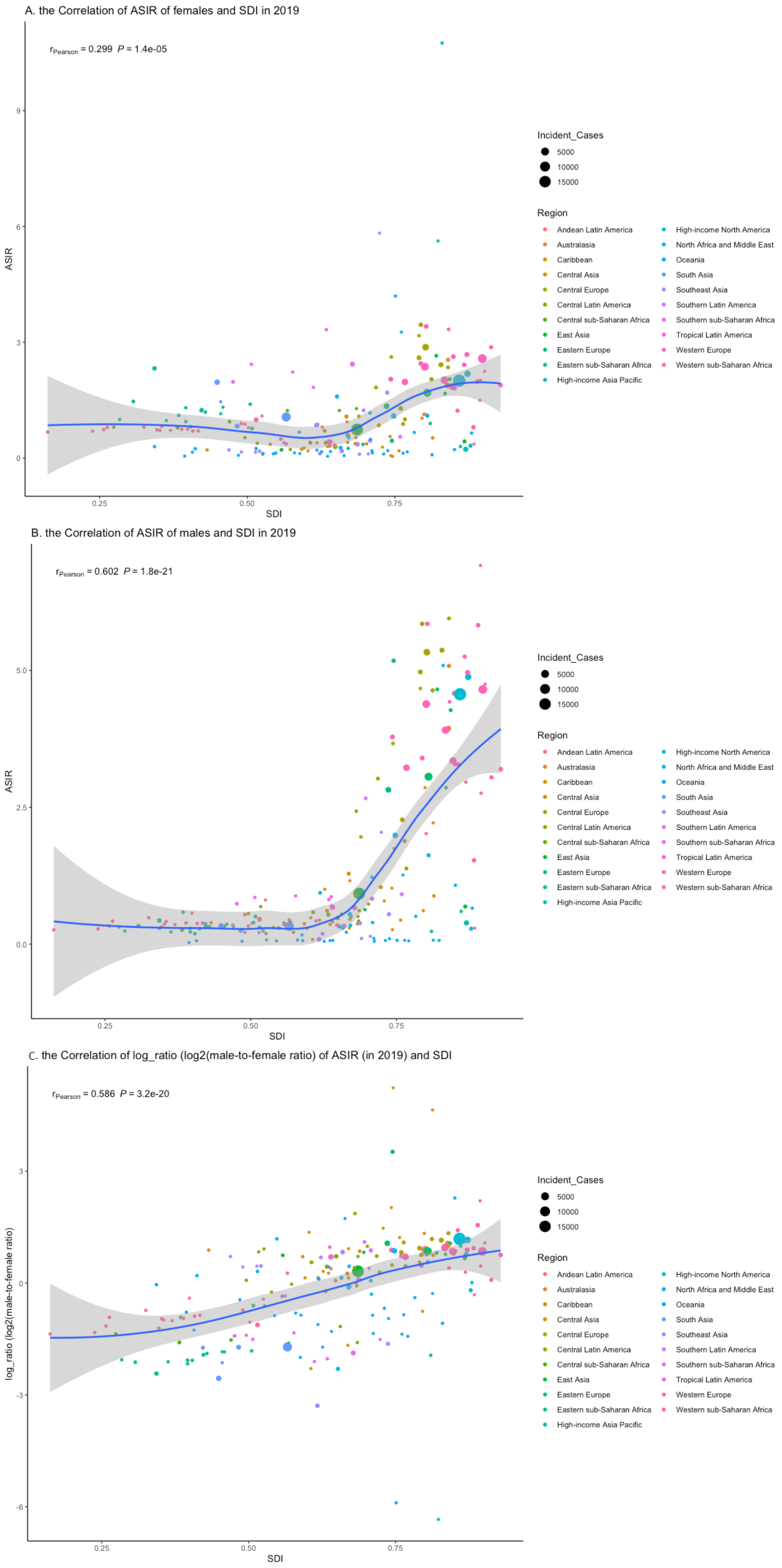

Supplementary Figure S3. The correlation between EAPCs of (A) ASIR of females, (B) ASIR of males and (C) the log_ratios (log2(male-to-female ratio)) of ASIRs from 1990 to 2019 and SDI in 2019 (ASIR, age-standardized incidence rate).
