# Supplementary material for: Trends in Disease Burden of Chronic Lymphocytic Leukemia at the Global, Regional, and National Levels From 1990 to 2019, and Projections Until 2030: A Population-Based Epidemiologic Study
Source: Front Oncol. 2022 Mar 10;12:840616. doi: 10.3389/fonc.2022.840616 (PMC8961301; doi:10.3389/fonc.2022.840616)
Supplement: Supplementary Table S1 — Age-standardized incidence rates by genders in different SDI quintiles from 1990 to 2019. SDI, socio-demographic index. [file Table_1.docx]

**Supplementary table S1. Age-standardized incidence rates by genders in different SDI quintiles from 1990 to 2019.**

| Gender | Year | Age-standardized incidence rate  per 100,000  (95% UI) | | | | | |
| --- | --- | --- | --- | --- | --- | --- | --- |
|  |  | Global | High SDI | High-middle SDI | Middle SDI | Low-middle SDI | Low SDI |
| both | 1990 | 1.09 (1, 1.14) | 2.24 (2.05, 2.39) | 0.97 (0.88, 1.06) | 0.3 (0.25, 0.34) | 0.42 (0.35, 0.5) | 0.51 (0.4, 0.63) |
|  | 1991 | 1.1 (1.02, 1.17) | 2.28 (2.09, 2.44) | 1 (0.9, 1.07) | 0.28 (0.24, 0.32) | 0.42 (0.35, 0.5) | 0.5 (0.39, 0.61) |
|  | 1992 | 1.12 (1.03, 1.19) | 2.34 (2.13, 2.51) | 1.02 (0.94, 1.09) | 0.29 (0.25, 0.32) | 0.42 (0.35, 0.5) | 0.5 (0.39, 0.62) |
|  | 1993 | 1.16 (1.07, 1.23) | 2.43 (2.22, 2.62) | 1.06 (0.99, 1.13) | 0.29 (0.25, 0.32) | 0.42 (0.36, 0.5) | 0.5 (0.4, 0.62) |
|  | 1994 | 1.17 (1.09, 1.24) | 2.47 (2.25, 2.67) | 1.08 (1.01, 1.15) | 0.3 (0.26, 0.33) | 0.43 (0.36, 0.5) | 0.5 (0.4, 0.62) |
|  | 1995 | 1.18 (1.09, 1.26) | 2.5 (2.29, 2.71) | 1.08 (1.02, 1.16) | 0.3 (0.26, 0.34) | 0.43 (0.37, 0.51) | 0.5 (0.4, 0.62) |
|  | 1996 | 1.18 (1.09, 1.26) | 2.5 (2.29, 2.71) | 1.09 (1.02, 1.15) | 0.31 (0.27, 0.34) | 0.44 (0.37, 0.51) | 0.51 (0.4, 0.62) |
|  | 1997 | 1.19 (1.11, 1.27) | 2.51 (2.31, 2.72) | 1.11 (1.04, 1.17) | 0.32 (0.28, 0.36) | 0.46 (0.39, 0.54) | 0.52 (0.41, 0.64) |
|  | 1998 | 1.21 (1.13, 1.3) | 2.55 (2.34, 2.77) | 1.15 (1.07, 1.21) | 0.33 (0.28, 0.36) | 0.46 (0.39, 0.54) | 0.52 (0.42, 0.64) |
|  | 1999 | 1.24 (1.15, 1.33) | 2.62 (2.4, 2.85) | 1.2 (1.13, 1.27) | 0.33 (0.29, 0.37) | 0.46 (0.39, 0.53) | 0.53 (0.42, 0.63) |
|  | 2000 | 1.25 (1.17, 1.34) | 2.63 (2.41, 2.88) | 1.23 (1.15, 1.3) | 0.34 (0.29, 0.37) | 0.46 (0.39, 0.53) | 0.53 (0.42, 0.64) |
|  | 2001 | 1.27 (1.18, 1.37) | 2.68 (2.45, 2.93) | 1.25 (1.17, 1.33) | 0.34 (0.3, 0.38) | 0.47 (0.4, 0.54) | 0.55 (0.44, 0.66) |
|  | 2002 | 1.29 (1.2, 1.39) | 2.73 (2.49, 3) | 1.3 (1.21, 1.39) | 0.35 (0.31, 0.39) | 0.47 (0.41, 0.55) | 0.56 (0.44, 0.67) |
|  | 2003 | 1.3 (1.21, 1.41) | 2.74 (2.51, 3.05) | 1.34 (1.26, 1.45) | 0.36 (0.32, 0.4) | 0.47 (0.4, 0.53) | 0.57 (0.46, 0.68) |
|  | 2004 | 1.28 (1.19, 1.4) | 2.66 (2.45, 2.99) | 1.35 (1.26, 1.47) | 0.37 (0.33, 0.41) | 0.45 (0.39, 0.52) | 0.57 (0.46, 0.68) |
|  | 2005 | 1.28 (1.19, 1.41) | 2.64 (2.44, 2.98) | 1.39 (1.3, 1.52) | 0.38 (0.34, 0.42) | 0.46 (0.41, 0.53) | 0.58 (0.47, 0.69) |
|  | 2006 | 1.27 (1.18, 1.41) | 2.59 (2.41, 2.94) | 1.39 (1.3, 1.52) | 0.39 (0.35, 0.44) | 0.48 (0.42, 0.55) | 0.58 (0.47, 0.7) |
|  | 2007 | 1.28 (1.19, 1.42) | 2.56 (2.38, 2.95) | 1.43 (1.33, 1.56) | 0.41 (0.37, 0.46) | 0.48 (0.42, 0.56) | 0.58 (0.47, 0.69) |
|  | 2008 | 1.3 (1.2, 1.45) | 2.56 (2.38, 2.97) | 1.47 (1.37, 1.61) | 0.44 (0.39, 0.49) | 0.49 (0.43, 0.56) | 0.59 (0.48, 0.7) |
|  | 2009 | 1.3 (1.2, 1.46) | 2.54 (2.35, 2.98) | 1.49 (1.39, 1.64) | 0.46 (0.41, 0.52) | 0.48 (0.42, 0.55) | 0.59 (0.48, 0.7) |
|  | 2010 | 1.29 (1.2, 1.46) | 2.48 (2.3, 2.93) | 1.5 (1.4, 1.65) | 0.49 (0.44, 0.56) | 0.49 (0.43, 0.55) | 0.6 (0.49, 0.7) |
|  | 2011 | 1.3 (1.2, 1.46) | 2.46 (2.27, 2.91) | 1.5 (1.41, 1.66) | 0.51 (0.46, 0.59) | 0.5 (0.45, 0.57) | 0.61 (0.5, 0.72) |
|  | 2012 | 1.29 (1.2, 1.46) | 2.41 (2.23, 2.86) | 1.5 (1.39, 1.65) | 0.52 (0.47, 0.6) | 0.51 (0.46, 0.58) | 0.62 (0.51, 0.74) |
|  | 2013 | 1.28 (1.18, 1.47) | 2.38 (2.19, 2.84) | 1.5 (1.39, 1.65) | 0.54 (0.48, 0.62) | 0.53 (0.47, 0.61) | 0.63 (0.52, 0.74) |
|  | 2014 | 1.27 (1.18, 1.45) | 2.3 (2.12, 2.78) | 1.5 (1.4, 1.66) | 0.56 (0.5, 0.64) | 0.54 (0.48, 0.62) | 0.64 (0.53, 0.75) |
|  | 2015 | 1.27 (1.18, 1.46) | 2.26 (2.07, 2.76) | 1.53 (1.43, 1.7) | 0.57 (0.51, 0.65) | 0.57 (0.51, 0.64) | 0.65 (0.53, 0.76) |
|  | 2016 | 1.26 (1.16, 1.45) | 2.24 (2.05, 2.75) | 1.52 (1.41, 1.68) | 0.57 (0.51, 0.66) | 0.58 (0.51, 0.66) | 0.66 (0.54, 0.77) |
|  | 2017 | 1.26 (1.15, 1.44) | 2.24 (2.02, 2.74) | 1.51 (1.39, 1.68) | 0.59 (0.52, 0.68) | 0.59 (0.52, 0.68) | 0.66 (0.55, 0.78) |
|  | 2018 | 1.28 (1.16, 1.48) | 2.27 (2, 2.82) | 1.52 (1.39, 1.7) | 0.61 (0.54, 0.71) | 0.61 (0.53, 0.69) | 0.68 (0.56, 0.8) |
|  | 2019 | 1.28 (1.16, 1.48) | 2.29 (1.99, 2.82) | 1.53 (1.38, 1.71) | 0.62 (0.54, 0.72) | 0.62 (0.54, 0.71) | 0.69 (0.58, 0.82) |
| female | 1990 | 0.92 (0.85, 0.98) | 1.62 (1.49, 1.73) | 0.74 (0.68, 0.81) | 0.33 (0.27, 0.41) | 0.6 (0.48, 0.74) | 0.78 (0.58, 1.02) |
|  | 1991 | 0.93 (0.86, 1) | 1.66 (1.52, 1.77) | 0.76 (0.7, 0.82) | 0.34 (0.27, 0.42) | 0.6 (0.47, 0.74) | 0.78 (0.58, 1.01) |
|  | 1992 | 0.95 (0.87, 1.02) | 1.68 (1.55, 1.8) | 0.78 (0.72, 0.84) | 0.35 (0.28, 0.43) | 0.6 (0.47, 0.73) | 0.79 (0.58, 1.01) |
|  | 1993 | 0.98 (0.9, 1.05) | 1.76 (1.62, 1.89) | 0.82 (0.76, 0.88) | 0.35 (0.28, 0.43) | 0.6 (0.48, 0.75) | 0.79 (0.59, 1.01) |
|  | 1994 | 0.99 (0.91, 1.07) | 1.79 (1.65, 1.93) | 0.84 (0.78, 0.9) | 0.36 (0.29, 0.44) | 0.61 (0.49, 0.75) | 0.79 (0.59, 1.01) |
|  | 1995 | 1 (0.92, 1.07) | 1.81 (1.67, 1.95) | 0.85 (0.79, 0.91) | 0.36 (0.29, 0.44) | 0.61 (0.49, 0.75) | 0.79 (0.6, 1.01) |
|  | 1996 | 1 (0.93, 1.08) | 1.81 (1.67, 1.97) | 0.85 (0.79, 0.91) | 0.37 (0.3, 0.45) | 0.63 (0.51, 0.75) | 0.79 (0.6, 1) |
|  | 1997 | 1.01 (0.94, 1.09) | 1.82 (1.67, 1.99) | 0.86 (0.8, 0.92) | 0.39 (0.31, 0.47) | 0.65 (0.53, 0.78) | 0.8 (0.61, 1.03) |
|  | 1998 | 1.03 (0.95, 1.11) | 1.84 (1.69, 2.03) | 0.89 (0.83, 0.96) | 0.39 (0.31, 0.47) | 0.66 (0.54, 0.79) | 0.82 (0.63, 1.03) |
|  | 1999 | 1.05 (0.97, 1.13) | 1.9 (1.74, 2.1) | 0.93 (0.87, 1.01) | 0.39 (0.32, 0.48) | 0.65 (0.53, 0.77) | 0.82 (0.63, 1.03) |
|  | 2000 | 1.06 (0.98, 1.15) | 1.91 (1.75, 2.11) | 0.96 (0.89, 1.04) | 0.4 (0.32, 0.49) | 0.65 (0.53, 0.77) | 0.83 (0.64, 1.03) |
|  | 2001 | 1.07 (0.98, 1.16) | 1.93 (1.76, 2.14) | 0.97 (0.9, 1.05) | 0.4 (0.33, 0.49) | 0.66 (0.54, 0.78) | 0.85 (0.66, 1.05) |
|  | 2002 | 1.08 (1, 1.18) | 1.96 (1.8, 2.17) | 1 (0.93, 1.09) | 0.41 (0.33, 0.49) | 0.67 (0.55, 0.79) | 0.87 (0.69, 1.07) |
|  | 2003 | 1.09 (1, 1.2) | 1.96 (1.79, 2.2) | 1.04 (0.97, 1.14) | 0.41 (0.33, 0.5) | 0.65 (0.54, 0.78) | 0.88 (0.69, 1.08) |
|  | 2004 | 1.06 (0.98, 1.18) | 1.89 (1.73, 2.14) | 1.05 (0.96, 1.15) | 0.42 (0.34, 0.5) | 0.62 (0.52, 0.74) | 0.89 (0.7, 1.1) |
|  | 2005 | 1.07 (0.98, 1.19) | 1.86 (1.7, 2.13) | 1.07 (0.99, 1.19) | 0.43 (0.35, 0.52) | 0.64 (0.54, 0.76) | 0.9 (0.72, 1.09) |
|  | 2006 | 1.06 (0.97, 1.18) | 1.82 (1.66, 2.09) | 1.07 (0.99, 1.18) | 0.45 (0.37, 0.54) | 0.66 (0.55, 0.78) | 0.91 (0.72, 1.11) |
|  | 2007 | 1.06 (0.97, 1.19) | 1.8 (1.64, 2.06) | 1.11 (1.01, 1.23) | 0.46 (0.38, 0.56) | 0.66 (0.56, 0.79) | 0.91 (0.72, 1.11) |
|  | 2008 | 1.08 (0.99, 1.2) | 1.79 (1.63, 2.07) | 1.15 (1.05, 1.27) | 0.49 (0.4, 0.6) | 0.66 (0.56, 0.8) | 0.91 (0.73, 1.11) |
|  | 2009 | 1.08 (0.99, 1.22) | 1.77 (1.61, 2.06) | 1.17 (1.08, 1.31) | 0.52 (0.42, 0.63) | 0.65 (0.54, 0.78) | 0.91 (0.73, 1.11) |
|  | 2010 | 1.08 (0.98, 1.21) | 1.7 (1.54, 2) | 1.18 (1.09, 1.31) | 0.55 (0.45, 0.68) | 0.66 (0.56, 0.78) | 0.93 (0.75, 1.12) |
|  | 2011 | 1.09 (1, 1.23) | 1.71 (1.55, 2.02) | 1.18 (1.1, 1.33) | 0.58 (0.46, 0.71) | 0.69 (0.58, 0.82) | 0.94 (0.76, 1.14) |
|  | 2012 | 1.08 (0.99, 1.23) | 1.68 (1.52, 1.99) | 1.18 (1.08, 1.33) | 0.59 (0.48, 0.72) | 0.7 (0.59, 0.83) | 0.97 (0.79, 1.17) |
|  | 2013 | 1.08 (0.99, 1.23) | 1.65 (1.5, 1.97) | 1.18 (1.08, 1.33) | 0.61 (0.49, 0.74) | 0.72 (0.61, 0.84) | 0.98 (0.79, 1.19) |
|  | 2014 | 1.08 (0.98, 1.22) | 1.6 (1.44, 1.89) | 1.18 (1.08, 1.34) | 0.62 (0.5, 0.78) | 0.74 (0.62, 0.87) | 1 (0.8, 1.19) |
|  | 2015 | 1.08 (0.99, 1.23) | 1.57 (1.41, 1.86) | 1.22 (1.11, 1.38) | 0.63 (0.51, 0.78) | 0.78 (0.67, 0.91) | 1.01 (0.81, 1.2) |
|  | 2016 | 1.08 (0.98, 1.22) | 1.55 (1.39, 1.84) | 1.2 (1.1, 1.36) | 0.64 (0.52, 0.79) | 0.8 (0.68, 0.93) | 1.02 (0.83, 1.22) |
|  | 2017 | 1.08 (0.97, 1.23) | 1.53 (1.36, 1.84) | 1.2 (1.08, 1.36) | 0.65 (0.53, 0.82) | 0.81 (0.68, 0.96) | 1.03 (0.84, 1.23) |
|  | 2018 | 1.09 (0.98, 1.26) | 1.54 (1.35, 1.84) | 1.22 (1.09, 1.42) | 0.68 (0.54, 0.86) | 0.84 (0.68, 0.98) | 1.06 (0.86, 1.28) |
|  | 2019 | 1.1 (0.98, 1.27) | 1.56 (1.34, 1.87) | 1.23 (1.08, 1.43) | 0.69 (0.55, 0.88) | 0.86 (0.71, 1.02) | 1.08 (0.88, 1.3) |
| male | 1990 | 1.31 (1.15, 1.4) | 3.1 (2.69, 3.46) | 1.3 (1.1, 1.52) | 0.26 (0.2, 0.32) | 0.24 (0.17, 0.31) | 0.24 (0.14, 0.34) |
|  | 1991 | 1.33 (1.18, 1.42) | 3.16 (2.75, 3.54) | 1.33 (1.13, 1.54) | 0.22 (0.18, 0.26) | 0.24 (0.17, 0.31) | 0.21 (0.12, 0.3) |
|  | 1992 | 1.36 (1.2, 1.46) | 3.24 (2.8, 3.62) | 1.36 (1.18, 1.56) | 0.23 (0.18, 0.27) | 0.24 (0.17, 0.31) | 0.21 (0.13, 0.31) |
|  | 1993 | 1.4 (1.24, 1.52) | 3.36 (2.91, 3.77) | 1.39 (1.24, 1.58) | 0.23 (0.19, 0.26) | 0.24 (0.18, 0.31) | 0.22 (0.13, 0.31) |
|  | 1994 | 1.41 (1.26, 1.54) | 3.42 (2.97, 3.83) | 1.41 (1.27, 1.6) | 0.24 (0.19, 0.27) | 0.25 (0.18, 0.31) | 0.22 (0.13, 0.31) |
|  | 1995 | 1.42 (1.28, 1.55) | 3.46 (3.01, 3.89) | 1.42 (1.29, 1.63) | 0.24 (0.2, 0.28) | 0.25 (0.18, 0.31) | 0.22 (0.13, 0.3) |
|  | 1996 | 1.42 (1.26, 1.55) | 3.45 (3.02, 3.87) | 1.44 (1.29, 1.61) | 0.24 (0.2, 0.28) | 0.25 (0.19, 0.31) | 0.22 (0.13, 0.31) |
|  | 1997 | 1.43 (1.27, 1.55) | 3.45 (3.04, 3.86) | 1.47 (1.3, 1.63) | 0.25 (0.2, 0.28) | 0.26 (0.19, 0.33) | 0.22 (0.14, 0.31) |
|  | 1998 | 1.46 (1.3, 1.59) | 3.51 (3.1, 3.94) | 1.52 (1.36, 1.66) | 0.26 (0.21, 0.29) | 0.26 (0.2, 0.33) | 0.23 (0.14, 0.31) |
|  | 1999 | 1.49 (1.35, 1.64) | 3.57 (3.18, 4.05) | 1.58 (1.44, 1.71) | 0.26 (0.21, 0.29) | 0.26 (0.2, 0.32) | 0.23 (0.14, 0.31) |
|  | 2000 | 1.51 (1.36, 1.65) | 3.59 (3.22, 4.09) | 1.62 (1.48, 1.75) | 0.27 (0.22, 0.31) | 0.26 (0.2, 0.32) | 0.23 (0.15, 0.31) |
|  | 2001 | 1.53 (1.39, 1.68) | 3.66 (3.29, 4.19) | 1.65 (1.51, 1.78) | 0.28 (0.23, 0.32) | 0.27 (0.21, 0.33) | 0.23 (0.15, 0.32) |
|  | 2002 | 1.56 (1.41, 1.73) | 3.72 (3.37, 4.27) | 1.71 (1.56, 1.85) | 0.3 (0.25, 0.34) | 0.27 (0.22, 0.33) | 0.24 (0.15, 0.33) |
|  | 2003 | 1.58 (1.44, 1.77) | 3.75 (3.39, 4.33) | 1.76 (1.62, 1.94) | 0.3 (0.25, 0.36) | 0.28 (0.22, 0.33) | 0.24 (0.16, 0.33) |
|  | 2004 | 1.56 (1.42, 1.74) | 3.66 (3.31, 4.21) | 1.78 (1.63, 1.95) | 0.31 (0.26, 0.36) | 0.27 (0.22, 0.32) | 0.24 (0.16, 0.33) |
|  | 2005 | 1.57 (1.43, 1.75) | 3.63 (3.3, 4.21) | 1.82 (1.68, 2.01) | 0.32 (0.27, 0.37) | 0.28 (0.22, 0.32) | 0.24 (0.16, 0.33) |
|  | 2006 | 1.55 (1.42, 1.75) | 3.57 (3.25, 4.15) | 1.83 (1.67, 2.01) | 0.33 (0.28, 0.39) | 0.29 (0.23, 0.34) | 0.24 (0.16, 0.33) |
|  | 2007 | 1.56 (1.42, 1.77) | 3.53 (3.23, 4.15) | 1.87 (1.71, 2.05) | 0.35 (0.3, 0.42) | 0.29 (0.24, 0.34) | 0.24 (0.17, 0.33) |
|  | 2008 | 1.58 (1.44, 1.8) | 3.53 (3.23, 4.21) | 1.91 (1.76, 2.12) | 0.38 (0.32, 0.44) | 0.3 (0.25, 0.35) | 0.25 (0.17, 0.33) |
|  | 2009 | 1.58 (1.45, 1.82) | 3.51 (3.2, 4.25) | 1.93 (1.77, 2.14) | 0.4 (0.34, 0.47) | 0.3 (0.25, 0.35) | 0.25 (0.18, 0.33) |
|  | 2010 | 1.58 (1.45, 1.83) | 3.46 (3.18, 4.21) | 1.95 (1.79, 2.19) | 0.42 (0.36, 0.5) | 0.29 (0.25, 0.35) | 0.25 (0.17, 0.33) |
|  | 2011 | 1.57 (1.43, 1.83) | 3.39 (3.11, 4.15) | 1.94 (1.79, 2.19) | 0.44 (0.37, 0.52) | 0.3 (0.26, 0.36) | 0.26 (0.18, 0.34) |
|  | 2012 | 1.56 (1.42, 1.83) | 3.33 (3.04, 4.12) | 1.94 (1.79, 2.2) | 0.46 (0.38, 0.54) | 0.31 (0.26, 0.37) | 0.26 (0.18, 0.34) |
|  | 2013 | 1.54 (1.41, 1.82) | 3.27 (2.98, 4.1) | 1.93 (1.79, 2.17) | 0.47 (0.39, 0.55) | 0.31 (0.27, 0.38) | 0.26 (0.18, 0.34) |
|  | 2014 | 1.53 (1.4, 1.82) | 3.18 (2.88, 4.07) | 1.94 (1.79, 2.19) | 0.48 (0.41, 0.58) | 0.32 (0.27, 0.4) | 0.26 (0.18, 0.33) |
|  | 2015 | 1.52 (1.39, 1.82) | 3.12 (2.83, 4.01) | 1.96 (1.81, 2.24) | 0.49 (0.41, 0.59) | 0.33 (0.28, 0.41) | 0.26 (0.18, 0.34) |
|  | 2016 | 1.5 (1.38, 1.81) | 3.1 (2.8, 3.98) | 1.94 (1.78, 2.22) | 0.5 (0.41, 0.6) | 0.33 (0.28, 0.42) | 0.27 (0.19, 0.35) |
|  | 2017 | 1.5 (1.35, 1.81) | 3.11 (2.76, 4) | 1.92 (1.75, 2.17) | 0.51 (0.42, 0.62) | 0.34 (0.29, 0.43) | 0.27 (0.18, 0.34) |
|  | 2018 | 1.51 (1.36, 1.83) | 3.15 (2.7, 4.14) | 1.92 (1.71, 2.19) | 0.53 (0.42, 0.64) | 0.35 (0.29, 0.44) | 0.27 (0.19, 0.35) |
|  | 2019 | 1.52 (1.34, 1.83) | 3.16 (2.69, 4.1) | 1.92 (1.7, 2.21) | 0.54 (0.44, 0.65) | 0.36 (0.3, 0.44) | 0.28 (0.19, 0.35) |

SDI, socio-demographic index.

**Supplementary table S2. Age-standardized death rate by gender in different SDI quintiles from 1990 to 2019.**

| Gender | Year | Age-standardized death rate  per 100,000  (95% UI) | | | | | |
| --- | --- | --- | --- | --- | --- | --- | --- |
|  |  | Global | High SDI | High-middle SDI | Middle SDI | Low-middle SDI | Low SDI |
| both | 1990 | 0.62 (0.56, 0.66) | 0.94 (0.85, 1.01) | 0.58 (0.52, 0.63) | 0.26 (0.22, 0.29) | 0.42 (0.35, 0.5) | 0.53 (0.41, 0.64) |
|  | 1991 | 0.62 (0.56, 0.66) | 0.94 (0.85, 1.01) | 0.58 (0.53, 0.64) | 0.26 (0.23, 0.29) | 0.42 (0.35, 0.49) | 0.53 (0.42, 0.66) |
|  | 1992 | 0.62 (0.57, 0.66) | 0.94 (0.85, 1.01) | 0.59 (0.54, 0.64) | 0.27 (0.23, 0.3) | 0.42 (0.35, 0.5) | 0.53 (0.41, 0.66) |
|  | 1993 | 0.63 (0.58, 0.67) | 0.95 (0.86, 1.02) | 0.61 (0.57, 0.66) | 0.27 (0.23, 0.3) | 0.42 (0.35, 0.5) | 0.53 (0.42, 0.65) |
|  | 1994 | 0.63 (0.58, 0.67) | 0.94 (0.85, 1.02) | 0.62 (0.58, 0.67) | 0.27 (0.23, 0.3) | 0.42 (0.35, 0.5) | 0.53 (0.42, 0.66) |
|  | 1995 | 0.63 (0.57, 0.67) | 0.94 (0.85, 1.01) | 0.62 (0.58, 0.67) | 0.27 (0.24, 0.3) | 0.42 (0.36, 0.5) | 0.53 (0.42, 0.66) |
|  | 1996 | 0.62 (0.57, 0.66) | 0.93 (0.84, 1) | 0.61 (0.57, 0.66) | 0.28 (0.24, 0.3) | 0.43 (0.36, 0.5) | 0.54 (0.42, 0.66) |
|  | 1997 | 0.62 (0.57, 0.66) | 0.92 (0.83, 0.99) | 0.61 (0.57, 0.66) | 0.28 (0.25, 0.31) | 0.45 (0.38, 0.52) | 0.55 (0.43, 0.67) |
|  | 1998 | 0.63 (0.57, 0.67) | 0.92 (0.83, 1) | 0.62 (0.58, 0.66) | 0.29 (0.25, 0.31) | 0.45 (0.38, 0.53) | 0.55 (0.44, 0.67) |
|  | 1999 | 0.63 (0.58, 0.67) | 0.93 (0.84, 1.01) | 0.64 (0.6, 0.68) | 0.29 (0.25, 0.31) | 0.45 (0.38, 0.52) | 0.55 (0.44, 0.67) |
|  | 2000 | 0.63 (0.58, 0.67) | 0.93 (0.83, 1.01) | 0.65 (0.6, 0.69) | 0.29 (0.25, 0.31) | 0.45 (0.38, 0.52) | 0.56 (0.44, 0.68) |
|  | 2001 | 0.63 (0.58, 0.68) | 0.93 (0.83, 1.02) | 0.65 (0.6, 0.69) | 0.29 (0.26, 0.32) | 0.45 (0.39, 0.52) | 0.57 (0.45, 0.69) |
|  | 2002 | 0.64 (0.59, 0.68) | 0.93 (0.83, 1.02) | 0.67 (0.62, 0.71) | 0.29 (0.26, 0.32) | 0.46 (0.39, 0.53) | 0.58 (0.46, 0.7) |
|  | 2003 | 0.64 (0.58, 0.68) | 0.92 (0.83, 1.02) | 0.67 (0.63, 0.73) | 0.29 (0.26, 0.32) | 0.45 (0.38, 0.52) | 0.59 (0.47, 0.71) |
|  | 2004 | 0.62 (0.57, 0.67) | 0.89 (0.8, 0.98) | 0.67 (0.62, 0.72) | 0.29 (0.26, 0.32) | 0.43 (0.37, 0.49) | 0.59 (0.47, 0.71) |
|  | 2005 | 0.62 (0.56, 0.67) | 0.87 (0.79, 0.97) | 0.67 (0.62, 0.73) | 0.29 (0.27, 0.33) | 0.44 (0.38, 0.5) | 0.6 (0.48, 0.71) |
|  | 2006 | 0.61 (0.56, 0.66) | 0.85 (0.77, 0.96) | 0.67 (0.61, 0.72) | 0.3 (0.27, 0.33) | 0.45 (0.39, 0.52) | 0.6 (0.48, 0.72) |
|  | 2007 | 0.61 (0.55, 0.67) | 0.84 (0.75, 0.95) | 0.67 (0.61, 0.72) | 0.3 (0.28, 0.34) | 0.45 (0.39, 0.52) | 0.6 (0.48, 0.72) |
|  | 2008 | 0.61 (0.55, 0.67) | 0.82 (0.74, 0.95) | 0.67 (0.61, 0.73) | 0.31 (0.28, 0.35) | 0.45 (0.4, 0.52) | 0.6 (0.49, 0.72) |
|  | 2009 | 0.6 (0.55, 0.66) | 0.81 (0.72, 0.94) | 0.66 (0.61, 0.72) | 0.32 (0.29, 0.36) | 0.44 (0.38, 0.5) | 0.6 (0.49, 0.71) |
|  | 2010 | 0.6 (0.54, 0.66) | 0.79 (0.7, 0.92) | 0.66 (0.6, 0.72) | 0.33 (0.3, 0.37) | 0.44 (0.38, 0.51) | 0.6 (0.49, 0.72) |
|  | 2011 | 0.6 (0.54, 0.66) | 0.78 (0.7, 0.92) | 0.65 (0.6, 0.71) | 0.33 (0.3, 0.37) | 0.45 (0.4, 0.51) | 0.61 (0.5, 0.73) |
|  | 2012 | 0.59 (0.54, 0.66) | 0.77 (0.69, 0.91) | 0.64 (0.59, 0.71) | 0.34 (0.3, 0.38) | 0.45 (0.4, 0.51) | 0.62 (0.51, 0.74) |
|  | 2013 | 0.59 (0.53, 0.66) | 0.75 (0.67, 0.9) | 0.63 (0.58, 0.7) | 0.34 (0.31, 0.38) | 0.46 (0.41, 0.53) | 0.63 (0.51, 0.75) |
|  | 2014 | 0.58 (0.53, 0.65) | 0.73 (0.65, 0.88) | 0.63 (0.57, 0.69) | 0.34 (0.31, 0.39) | 0.48 (0.42, 0.54) | 0.64 (0.52, 0.75) |
|  | 2015 | 0.58 (0.53, 0.65) | 0.72 (0.64, 0.87) | 0.63 (0.57, 0.69) | 0.34 (0.31, 0.39) | 0.49 (0.43, 0.56) | 0.64 (0.53, 0.76) |
|  | 2016 | 0.57 (0.52, 0.65) | 0.71 (0.63, 0.86) | 0.61 (0.56, 0.68) | 0.34 (0.31, 0.39) | 0.5 (0.44, 0.57) | 0.65 (0.53, 0.77) |
|  | 2017 | 0.57 (0.51, 0.64) | 0.7 (0.62, 0.85) | 0.6 (0.55, 0.67) | 0.34 (0.3, 0.39) | 0.5 (0.44, 0.58) | 0.65 (0.53, 0.77) |
|  | 2018 | 0.57 (0.51, 0.64) | 0.71 (0.63, 0.86) | 0.6 (0.54, 0.66) | 0.34 (0.31, 0.39) | 0.51 (0.44, 0.59) | 0.66 (0.54, 0.78) |
|  | 2019 | 0.57 (0.51, 0.64) | 0.72 (0.63, 0.87) | 0.6 (0.54, 0.66) | 0.34 (0.3, 0.4) | 0.52 (0.44, 0.6) | 0.67 (0.55, 0.8) |
| female | 1990 | 0.53 (0.48, 0.57) | 0.65 (0.58, 0.69) | 0.41 (0.37, 0.45) | 0.29 (0.23, 0.35) | 0.59 (0.47, 0.72) | 0.8 (0.6, 1.04) |
|  | 1991 | 0.53 (0.48, 0.57) | 0.65 (0.58, 0.69) | 0.41 (0.38, 0.45) | 0.29 (0.23, 0.35) | 0.58 (0.46, 0.72) | 0.8 (0.6, 1.05) |
|  | 1992 | 0.53 (0.48, 0.58) | 0.64 (0.57, 0.69) | 0.42 (0.39, 0.45) | 0.3 (0.24, 0.35) | 0.59 (0.46, 0.72) | 0.8 (0.6, 1.05) |
|  | 1993 | 0.54 (0.48, 0.59) | 0.65 (0.58, 0.69) | 0.44 (0.41, 0.47) | 0.29 (0.24, 0.35) | 0.59 (0.47, 0.74) | 0.8 (0.6, 1.04) |
|  | 1994 | 0.54 (0.49, 0.59) | 0.65 (0.57, 0.69) | 0.45 (0.42, 0.48) | 0.3 (0.24, 0.36) | 0.59 (0.47, 0.72) | 0.8 (0.6, 1.03) |
|  | 1995 | 0.54 (0.49, 0.59) | 0.64 (0.57, 0.68) | 0.45 (0.42, 0.48) | 0.3 (0.24, 0.36) | 0.59 (0.48, 0.72) | 0.8 (0.6, 1.03) |
|  | 1996 | 0.54 (0.48, 0.58) | 0.63 (0.56, 0.68) | 0.44 (0.41, 0.47) | 0.3 (0.25, 0.36) | 0.6 (0.49, 0.73) | 0.81 (0.61, 1.03) |
|  | 1997 | 0.54 (0.49, 0.59) | 0.63 (0.56, 0.68) | 0.44 (0.41, 0.47) | 0.31 (0.25, 0.37) | 0.62 (0.51, 0.75) | 0.82 (0.62, 1.04) |
|  | 1998 | 0.54 (0.49, 0.59) | 0.63 (0.56, 0.68) | 0.45 (0.41, 0.48) | 0.31 (0.25, 0.38) | 0.63 (0.51, 0.76) | 0.83 (0.63, 1.04) |
|  | 1999 | 0.55 (0.5, 0.6) | 0.64 (0.57, 0.69) | 0.46 (0.42, 0.5) | 0.31 (0.26, 0.37) | 0.62 (0.51, 0.75) | 0.83 (0.63, 1.04) |
|  | 2000 | 0.55 (0.5, 0.6) | 0.63 (0.56, 0.69) | 0.47 (0.43, 0.5) | 0.31 (0.26, 0.37) | 0.62 (0.51, 0.75) | 0.84 (0.65, 1.05) |
|  | 2001 | 0.55 (0.49, 0.6) | 0.63 (0.56, 0.69) | 0.47 (0.43, 0.51) | 0.31 (0.26, 0.37) | 0.62 (0.51, 0.74) | 0.86 (0.66, 1.06) |
|  | 2002 | 0.55 (0.5, 0.6) | 0.63 (0.56, 0.69) | 0.48 (0.44, 0.52) | 0.31 (0.26, 0.37) | 0.63 (0.52, 0.76) | 0.88 (0.68, 1.08) |
|  | 2003 | 0.54 (0.49, 0.6) | 0.62 (0.55, 0.68) | 0.49 (0.44, 0.53) | 0.31 (0.26, 0.36) | 0.61 (0.5, 0.73) | 0.88 (0.69, 1.08) |
|  | 2004 | 0.53 (0.48, 0.58) | 0.59 (0.52, 0.66) | 0.48 (0.44, 0.52) | 0.31 (0.25, 0.37) | 0.58 (0.48, 0.69) | 0.89 (0.69, 1.09) |
|  | 2005 | 0.53 (0.48, 0.59) | 0.58 (0.51, 0.65) | 0.48 (0.44, 0.53) | 0.31 (0.26, 0.36) | 0.59 (0.5, 0.71) | 0.89 (0.7, 1.1) |
|  | 2006 | 0.53 (0.47, 0.58) | 0.56 (0.5, 0.63) | 0.48 (0.43, 0.52) | 0.31 (0.27, 0.37) | 0.61 (0.51, 0.72) | 0.9 (0.72, 1.1) |
|  | 2007 | 0.52 (0.47, 0.58) | 0.55 (0.48, 0.62) | 0.48 (0.44, 0.53) | 0.32 (0.27, 0.38) | 0.61 (0.51, 0.73) | 0.9 (0.71, 1.09) |
|  | 2008 | 0.52 (0.47, 0.58) | 0.54 (0.47, 0.62) | 0.49 (0.44, 0.53) | 0.33 (0.27, 0.39) | 0.61 (0.51, 0.73) | 0.9 (0.71, 1.1) |
|  | 2009 | 0.52 (0.46, 0.57) | 0.53 (0.46, 0.61) | 0.48 (0.43, 0.53) | 0.33 (0.28, 0.39) | 0.58 (0.49, 0.69) | 0.89 (0.71, 1.08) |
|  | 2010 | 0.51 (0.46, 0.57) | 0.51 (0.44, 0.59) | 0.48 (0.43, 0.52) | 0.34 (0.28, 0.4) | 0.59 (0.49, 0.71) | 0.9 (0.72, 1.11) |
|  | 2011 | 0.52 (0.47, 0.58) | 0.51 (0.44, 0.59) | 0.47 (0.43, 0.52) | 0.34 (0.29, 0.41) | 0.61 (0.51, 0.72) | 0.91 (0.73, 1.12) |
|  | 2012 | 0.52 (0.46, 0.57) | 0.5 (0.43, 0.58) | 0.47 (0.42, 0.52) | 0.34 (0.29, 0.41) | 0.61 (0.52, 0.73) | 0.93 (0.75, 1.14) |
|  | 2013 | 0.51 (0.46, 0.57) | 0.49 (0.42, 0.58) | 0.46 (0.41, 0.51) | 0.35 (0.29, 0.41) | 0.63 (0.53, 0.75) | 0.95 (0.75, 1.14) |
|  | 2014 | 0.51 (0.46, 0.57) | 0.48 (0.41, 0.56) | 0.45 (0.41, 0.51) | 0.35 (0.29, 0.41) | 0.64 (0.54, 0.76) | 0.96 (0.77, 1.15) |
|  | 2015 | 0.51 (0.46, 0.57) | 0.47 (0.4, 0.55) | 0.46 (0.41, 0.51) | 0.35 (0.29, 0.42) | 0.67 (0.56, 0.78) | 0.96 (0.77, 1.15) |
|  | 2016 | 0.51 (0.46, 0.57) | 0.46 (0.39, 0.54) | 0.45 (0.4, 0.5) | 0.35 (0.29, 0.42) | 0.68 (0.57, 0.8) | 0.97 (0.78, 1.17) |
|  | 2017 | 0.5 (0.45, 0.57) | 0.45 (0.38, 0.54) | 0.44 (0.39, 0.49) | 0.35 (0.29, 0.42) | 0.68 (0.57, 0.82) | 0.98 (0.79, 1.18) |
|  | 2018 | 0.51 (0.45, 0.57) | 0.45 (0.38, 0.54) | 0.44 (0.39, 0.5) | 0.35 (0.29, 0.42) | 0.7 (0.57, 0.84) | 1 (0.81, 1.21) |
|  | 2019 | 0.51 (0.45, 0.57) | 0.45 (0.38, 0.54) | 0.44 (0.39, 0.5) | 0.35 (0.28, 0.43) | 0.71 (0.57, 0.84) | 1.01 (0.82, 1.23) |
| male | 1990 | 0.75 (0.66, 0.81) | 1.4 (1.21, 1.55) | 0.84 (0.71, 0.99) | 0.23 (0.18, 0.28) | 0.24 (0.17, 0.32) | 0.25 (0.15, 0.36) |
|  | 1991 | 0.75 (0.66, 0.81) | 1.4 (1.21, 1.55) | 0.84 (0.73, 0.99) | 0.23 (0.19, 0.28) | 0.25 (0.17, 0.32) | 0.25 (0.15, 0.36) |
|  | 1992 | 0.75 (0.66, 0.81) | 1.4 (1.21, 1.55) | 0.85 (0.75, 0.99) | 0.23 (0.19, 0.28) | 0.25 (0.17, 0.32) | 0.25 (0.15, 0.36) |
|  | 1993 | 0.76 (0.68, 0.82) | 1.41 (1.22, 1.58) | 0.87 (0.79, 1.01) | 0.24 (0.19, 0.28) | 0.25 (0.18, 0.32) | 0.26 (0.15, 0.36) |
|  | 1994 | 0.76 (0.68, 0.82) | 1.41 (1.22, 1.57) | 0.88 (0.8, 1.01) | 0.24 (0.19, 0.28) | 0.25 (0.18, 0.32) | 0.26 (0.16, 0.36) |
|  | 1995 | 0.76 (0.68, 0.82) | 1.4 (1.22, 1.58) | 0.88 (0.8, 1.02) | 0.24 (0.19, 0.28) | 0.25 (0.18, 0.31) | 0.26 (0.16, 0.36) |
|  | 1996 | 0.75 (0.67, 0.81) | 1.39 (1.21, 1.55) | 0.87 (0.79, 1) | 0.25 (0.2, 0.28) | 0.26 (0.19, 0.32) | 0.26 (0.16, 0.37) |
|  | 1997 | 0.75 (0.67, 0.81) | 1.37 (1.2, 1.52) | 0.88 (0.79, 0.98) | 0.25 (0.2, 0.28) | 0.26 (0.2, 0.33) | 0.26 (0.16, 0.37) |
|  | 1998 | 0.75 (0.67, 0.81) | 1.37 (1.2, 1.52) | 0.89 (0.8, 0.97) | 0.25 (0.2, 0.28) | 0.27 (0.2, 0.33) | 0.27 (0.17, 0.37) |
|  | 1999 | 0.76 (0.68, 0.82) | 1.37 (1.21, 1.54) | 0.91 (0.83, 0.99) | 0.25 (0.21, 0.29) | 0.26 (0.2, 0.32) | 0.27 (0.17, 0.37) |
|  | 2000 | 0.76 (0.68, 0.82) | 1.36 (1.21, 1.53) | 0.92 (0.84, 1) | 0.26 (0.21, 0.29) | 0.26 (0.2, 0.32) | 0.27 (0.17, 0.38) |
|  | 2001 | 0.77 (0.68, 0.83) | 1.37 (1.22, 1.56) | 0.93 (0.85, 1) | 0.26 (0.22, 0.3) | 0.27 (0.21, 0.32) | 0.28 (0.18, 0.38) |
|  | 2002 | 0.77 (0.68, 0.84) | 1.37 (1.21, 1.57) | 0.95 (0.86, 1.04) | 0.27 (0.23, 0.31) | 0.27 (0.21, 0.33) | 0.28 (0.18, 0.38) |
|  | 2003 | 0.77 (0.69, 0.85) | 1.36 (1.19, 1.57) | 0.96 (0.88, 1.05) | 0.27 (0.23, 0.31) | 0.27 (0.21, 0.32) | 0.28 (0.19, 0.39) |
|  | 2004 | 0.76 (0.67, 0.83) | 1.32 (1.16, 1.52) | 0.96 (0.87, 1.04) | 0.27 (0.23, 0.31) | 0.27 (0.21, 0.31) | 0.28 (0.19, 0.38) |
|  | 2005 | 0.75 (0.67, 0.84) | 1.3 (1.14, 1.5) | 0.96 (0.87, 1.06) | 0.28 (0.23, 0.32) | 0.27 (0.22, 0.32) | 0.28 (0.19, 0.38) |
|  | 2006 | 0.74 (0.66, 0.82) | 1.27 (1.12, 1.48) | 0.95 (0.86, 1.03) | 0.28 (0.24, 0.32) | 0.28 (0.22, 0.32) | 0.28 (0.19, 0.39) |
|  | 2007 | 0.74 (0.66, 0.82) | 1.25 (1.11, 1.46) | 0.94 (0.86, 1.03) | 0.29 (0.24, 0.33) | 0.28 (0.23, 0.32) | 0.28 (0.19, 0.38) |
|  | 2008 | 0.74 (0.66, 0.82) | 1.23 (1.1, 1.46) | 0.94 (0.85, 1.04) | 0.3 (0.25, 0.35) | 0.28 (0.23, 0.33) | 0.29 (0.2, 0.38) |
|  | 2009 | 0.72 (0.65, 0.82) | 1.2 (1.07, 1.44) | 0.93 (0.84, 1.03) | 0.3 (0.26, 0.35) | 0.28 (0.23, 0.33) | 0.29 (0.19, 0.38) |
|  | 2010 | 0.72 (0.65, 0.82) | 1.18 (1.06, 1.43) | 0.93 (0.84, 1.03) | 0.31 (0.27, 0.37) | 0.27 (0.23, 0.32) | 0.28 (0.2, 0.38) |
|  | 2011 | 0.72 (0.65, 0.81) | 1.16 (1.04, 1.41) | 0.92 (0.83, 1.02) | 0.32 (0.27, 0.38) | 0.28 (0.23, 0.33) | 0.29 (0.2, 0.38) |
|  | 2012 | 0.71 (0.64, 0.81) | 1.15 (1.02, 1.4) | 0.91 (0.83, 1.02) | 0.32 (0.27, 0.39) | 0.28 (0.23, 0.33) | 0.29 (0.2, 0.38) |
|  | 2013 | 0.7 (0.63, 0.81) | 1.12 (1, 1.39) | 0.89 (0.81, 1) | 0.33 (0.28, 0.39) | 0.28 (0.24, 0.34) | 0.29 (0.2, 0.38) |
|  | 2014 | 0.69 (0.62, 0.81) | 1.1 (0.97, 1.36) | 0.88 (0.8, 1) | 0.33 (0.28, 0.39) | 0.29 (0.24, 0.35) | 0.29 (0.2, 0.38) |
|  | 2015 | 0.69 (0.62, 0.8) | 1.07 (0.95, 1.34) | 0.88 (0.8, 1) | 0.33 (0.28, 0.39) | 0.29 (0.25, 0.36) | 0.29 (0.2, 0.38) |
|  | 2016 | 0.68 (0.61, 0.79) | 1.06 (0.93, 1.34) | 0.86 (0.78, 0.98) | 0.33 (0.28, 0.4) | 0.29 (0.25, 0.36) | 0.29 (0.2, 0.38) |
|  | 2017 | 0.67 (0.6, 0.79) | 1.06 (0.93, 1.34) | 0.84 (0.77, 0.96) | 0.33 (0.28, 0.4) | 0.3 (0.25, 0.36) | 0.29 (0.2, 0.38) |
|  | 2018 | 0.67 (0.6, 0.78) | 1.07 (0.94, 1.35) | 0.83 (0.75, 0.95) | 0.33 (0.28, 0.4) | 0.3 (0.25, 0.37) | 0.3 (0.2, 0.38) |
|  | 2019 | 0.66 (0.59, 0.78) | 1.07 (0.94, 1.35) | 0.82 (0.74, 0.94) | 0.33 (0.28, 0.41) | 0.3 (0.25, 0.38) | 0.3 (0.2, 0.38) |

SDI, socio-demographic index.

**Supplementary table S3. Age-standardized DALY rates by genders in different SDI quintiles from 1990 to 2019.**

| Gender | Year | Age-standardized DALY rate  per 100,000  (95% UI) | | | | | |
| --- | --- | --- | --- | --- | --- | --- | --- |
|  |  | Global | High SDI | High-middle SDI | Middle SDI | Low-middle SDI | Low SDI |
| both | 1990 | 12.5 (11.41, 13.48) | 18.53 (16.9, 19.96) | 12.9 (11.5, 14.3) | 6.26 (5.22, 7.22) | 9.17 (7.66, 10.92) | 11.24 (8.89, 13.74) |
|  | 1991 | 12.5 (11.45, 13.47) | 18.54 (16.92, 20.03) | 12.98 (11.69, 14.25) | 6.28 (5.3, 7.23) | 9.13 (7.66, 10.75) | 11.25 (8.87, 13.9) |
|  | 1992 | 12.55 (11.53, 13.51) | 18.52 (16.9, 19.95) | 13.17 (12.06, 14.26) | 6.34 (5.35, 7.26) | 9.15 (7.64, 10.83) | 11.3 (8.92, 13.96) |
|  | 1993 | 12.7 (11.78, 13.6) | 18.7 (17.04, 20.31) | 13.63 (12.81, 14.73) | 6.3 (5.38, 7.12) | 9.2 (7.72, 10.85) | 11.34 (8.92, 13.8) |
|  | 1994 | 12.77 (11.83, 13.72) | 18.66 (17.03, 20.27) | 13.84 (13.04, 15.04) | 6.38 (5.45, 7.22) | 9.27 (7.81, 10.89) | 11.37 (8.91, 13.85) |
|  | 1995 | 12.72 (11.85, 13.7) | 18.56 (16.99, 20.22) | 13.76 (13, 14.97) | 6.4 (5.44, 7.25) | 9.32 (7.89, 10.86) | 11.4 (9.01, 13.99) |
|  | 1996 | 12.62 (11.68, 13.49) | 18.32 (16.77, 19.99) | 13.5 (12.74, 14.53) | 6.48 (5.61, 7.26) | 9.48 (8.06, 11.05) | 11.45 (9.08, 13.91) |
|  | 1997 | 12.62 (11.68, 13.47) | 18.13 (16.61, 19.8) | 13.4 (12.54, 14.26) | 6.62 (5.71, 7.34) | 9.81 (8.34, 11.4) | 11.62 (9.28, 14.08) |
|  | 1998 | 12.64 (11.7, 13.55) | 18.15 (16.61, 19.82) | 13.46 (12.6, 14.33) | 6.67 (5.79, 7.41) | 9.86 (8.38, 11.46) | 11.75 (9.42, 14.17) |
|  | 1999 | 12.73 (11.83, 13.68) | 18.3 (16.79, 20.14) | 13.84 (12.99, 14.75) | 6.65 (5.74, 7.34) | 9.7 (8.27, 11.28) | 11.76 (9.35, 14.18) |
|  | 2000 | 12.73 (11.8, 13.65) | 18.2 (16.66, 20.08) | 13.97 (13.17, 14.93) | 6.69 (5.85, 7.38) | 9.7 (8.32, 11.22) | 11.89 (9.36, 14.32) |
|  | 2001 | 12.77 (11.88, 13.72) | 18.31 (16.77, 20.27) | 13.99 (13.17, 14.96) | 6.71 (5.95, 7.4) | 9.8 (8.45, 11.31) | 12.12 (9.67, 14.49) |
|  | 2002 | 12.82 (11.94, 13.87) | 18.35 (16.83, 20.37) | 14.19 (13.35, 15.27) | 6.7 (6.01, 7.37) | 9.85 (8.52, 11.42) | 12.36 (9.89, 14.71) |
|  | 2003 | 12.75 (11.92, 13.86) | 18.14 (16.59, 20.22) | 14.32 (13.48, 15.55) | 6.69 (5.97, 7.34) | 9.63 (8.36, 11.1) | 12.51 (10, 14.97) |
|  | 2004 | 12.49 (11.66, 13.58) | 17.54 (16.08, 19.67) | 14.23 (13.41, 15.39) | 6.69 (6.05, 7.36) | 9.32 (8.15, 10.69) | 12.54 (9.99, 14.89) |
|  | 2005 | 12.47 (11.66, 13.57) | 17.23 (15.84, 19.46) | 14.37 (13.48, 15.72) | 6.76 (6.13, 7.46) | 9.46 (8.31, 10.73) | 12.62 (10.2, 14.94) |
|  | 2006 | 12.33 (11.55, 13.47) | 16.81 (15.45, 19.09) | 14.1 (13.3, 15.33) | 6.85 (6.22, 7.54) | 9.64 (8.42, 11) | 12.66 (10.27, 14.91) |
|  | 2007 | 12.26 (11.47, 13.43) | 16.43 (15.14, 18.9) | 14.12 (13.24, 15.29) | 6.97 (6.35, 7.72) | 9.68 (8.53, 11.13) | 12.59 (10.2, 14.87) |
|  | 2008 | 12.29 (11.45, 13.53) | 16.16 (14.87, 18.79) | 14.24 (13.34, 15.48) | 7.23 (6.59, 8.03) | 9.72 (8.61, 11.21) | 12.62 (10.24, 14.94) |
|  | 2009 | 12.15 (11.33, 13.44) | 15.78 (14.48, 18.5) | 14.08 (13.16, 15.32) | 7.4 (6.66, 8.27) | 9.48 (8.37, 10.88) | 12.59 (10.32, 14.71) |
|  | 2010 | 12.1 (11.28, 13.43) | 15.3 (14.02, 18.15) | 14.01 (13.14, 15.31) | 7.64 (6.89, 8.58) | 9.61 (8.55, 11.21) | 12.7 (10.39, 14.97) |
|  | 2011 | 12.09 (11.22, 13.39) | 15.05 (13.77, 18.01) | 13.8 (12.96, 15.01) | 7.81 (7.09, 8.78) | 9.92 (8.85, 11.35) | 12.89 (10.57, 15.09) |
|  | 2012 | 11.98 (11.19, 13.34) | 14.72 (13.48, 17.62) | 13.59 (12.77, 14.86) | 7.86 (7.15, 8.86) | 10 (8.9, 11.4) | 13.08 (10.76, 15.25) |
|  | 2013 | 11.88 (11.06, 13.21) | 14.39 (13.15, 17.34) | 13.38 (12.55, 14.59) | 7.93 (7.21, 8.87) | 10.13 (9.03, 11.51) | 13.19 (10.82, 15.37) |
|  | 2014 | 11.77 (10.93, 13.14) | 13.93 (12.7, 16.89) | 13.24 (12.39, 14.53) | 8 (7.22, 9.01) | 10.31 (9.21, 11.67) | 13.29 (10.98, 15.47) |
|  | 2015 | 11.77 (10.97, 13.15) | 13.59 (12.35, 16.63) | 13.27 (12.37, 14.6) | 8.02 (7.23, 9.03) | 10.76 (9.56, 12.16) | 13.38 (11.14, 15.64) |
|  | 2016 | 11.67 (10.82, 13.06) | 13.41 (12.19, 16.42) | 13.03 (12.07, 14.41) | 8.01 (7.24, 9) | 10.89 (9.6, 12.38) | 13.46 (11.19, 15.73) |
|  | 2017 | 11.59 (10.75, 13.01) | 13.39 (12.14, 16.37) | 12.79 (11.88, 14.2) | 8.01 (7.19, 9.07) | 10.96 (9.62, 12.69) | 13.55 (11.21, 15.82) |
|  | 2018 | 11.63 (10.76, 13.06) | 13.54 (12.23, 16.59) | 12.76 (11.75, 14.21) | 8.03 (7.17, 9.15) | 11.09 (9.63, 12.73) | 13.75 (11.4, 16.1) |
|  | 2019 | 11.65 (10.72, 13.1) | 13.64 (12.28, 16.72) | 12.71 (11.69, 14.15) | 8.04 (7.12, 9.26) | 11.2 (9.67, 13.08) | 13.88 (11.55, 16.31) |
| female | 1990 | 10.77 (9.61, 12.03) | 12.09 (11.12, 12.92) | 8.98 (8.19, 10.05) | 6.86 (5.24, 8.56) | 12.96 (10.4, 15.91) | 17.18 (12.85, 21.8) |
|  | 1991 | 10.78 (9.72, 12.03) | 12.08 (11.11, 12.98) | 9.09 (8.31, 10.03) | 6.87 (5.26, 8.55) | 12.86 (10.29, 15.72) | 17.16 (12.95, 22.31) |
|  | 1992 | 10.84 (9.77, 12.08) | 11.99 (11.01, 12.86) | 9.23 (8.54, 10.08) | 6.99 (5.43, 8.65) | 12.91 (10.27, 15.96) | 17.24 (12.91, 22.32) |
|  | 1993 | 10.99 (9.9, 12.18) | 12.14 (11.18, 13.04) | 9.7 (9, 10.45) | 6.89 (5.32, 8.45) | 12.92 (10.37, 15.83) | 17.26 (12.97, 22.02) |
|  | 1994 | 11.13 (10.05, 12.37) | 12.11 (11.07, 13.05) | 9.99 (9.22, 10.84) | 7.06 (5.55, 8.89) | 13.02 (10.51, 15.89) | 17.27 (13.07, 22.02) |
|  | 1995 | 11.11 (10.02, 12.31) | 12.03 (11.04, 13.04) | 9.95 (9.24, 10.74) | 7.07 (5.56, 8.75) | 13.1 (10.55, 15.98) | 17.3 (13.18, 21.92) |
|  | 1996 | 11.06 (9.96, 12.22) | 11.87 (10.89, 12.97) | 9.7 (9.06, 10.59) | 7.16 (5.59, 8.92) | 13.31 (10.89, 16.09) | 17.35 (13.27, 21.99) |
|  | 1997 | 11.13 (10.03, 12.33) | 11.73 (10.77, 12.86) | 9.62 (8.93, 10.44) | 7.38 (5.77, 9.16) | 13.78 (11.25, 16.57) | 17.56 (13.48, 22.15) |
|  | 1998 | 11.15 (10.05, 12.34) | 11.73 (10.76, 12.88) | 9.64 (8.92, 10.54) | 7.43 (5.76, 9.3) | 13.81 (11.26, 16.5) | 17.77 (13.65, 22.35) |
|  | 1999 | 11.19 (10.13, 12.41) | 11.87 (10.88, 13.07) | 9.91 (9.25, 10.93) | 7.34 (5.79, 9.07) | 13.55 (11.12, 16.22) | 17.76 (13.75, 22.21) |
|  | 2000 | 11.16 (10.12, 12.39) | 11.76 (10.77, 12.95) | 9.98 (9.33, 11.01) | 7.31 (5.84, 9.01) | 13.49 (11.11, 16.05) | 17.92 (13.94, 22.63) |
|  | 2001 | 11.15 (10.1, 12.35) | 11.76 (10.73, 13) | 9.98 (9.33, 11) | 7.25 (5.77, 8.89) | 13.59 (11.34, 16.19) | 18.3 (14.19, 22.88) |
|  | 2002 | 11.14 (10.08, 12.37) | 11.76 (10.72, 13.04) | 10.05 (9.37, 11.06) | 7.11 (5.68, 8.7) | 13.63 (11.2, 16.3) | 18.69 (14.69, 22.98) |
|  | 2003 | 11.01 (9.95, 12.28) | 11.55 (10.48, 12.86) | 10.13 (9.4, 11.16) | 7 (5.69, 8.49) | 13.18 (10.9, 16) | 18.88 (14.7, 23.06) |
|  | 2004 | 10.73 (9.75, 11.99) | 11.05 (10.1, 12.41) | 10 (9.33, 11.11) | 6.94 (5.67, 8.47) | 12.67 (10.57, 15.16) | 18.95 (14.75, 23.18) |
|  | 2005 | 10.73 (9.71, 12.02) | 10.81 (9.82, 12.2) | 10.1 (9.37, 11.22) | 6.97 (5.79, 8.39) | 12.85 (10.74, 15.49) | 19.08 (15.01, 23.27) |
|  | 2006 | 10.65 (9.64, 11.93) | 10.51 (9.57, 11.91) | 9.91 (9.21, 11) | 7.07 (5.86, 8.54) | 13.09 (10.99, 15.54) | 19.16 (15.13, 23.39) |
|  | 2007 | 10.6 (9.68, 11.81) | 10.24 (9.28, 11.66) | 9.97 (9.3, 11.12) | 7.13 (5.96, 8.62) | 13.12 (11.14, 15.56) | 18.98 (15.07, 23.06) |
|  | 2008 | 10.64 (9.69, 11.96) | 10.05 (9.11, 11.53) | 10.09 (9.34, 11.16) | 7.36 (6.05, 8.88) | 13.11 (11.01, 15.51) | 19.03 (15.14, 23.13) |
|  | 2009 | 10.54 (9.54, 11.83) | 9.79 (8.83, 11.35) | 10.02 (9.28, 11.15) | 7.52 (6.17, 9.04) | 12.69 (10.68, 15.21) | 18.94 (15.26, 22.83) |
|  | 2010 | 10.56 (9.6, 11.92) | 9.35 (8.43, 10.88) | 9.95 (9.22, 10.99) | 7.8 (6.36, 9.36) | 13.02 (11.06, 15.55) | 19.19 (15.41, 23.23) |
|  | 2011 | 10.66 (9.64, 12.06) | 9.34 (8.41, 10.92) | 9.84 (9.11, 10.97) | 7.94 (6.46, 9.56) | 13.45 (11.48, 16.04) | 19.42 (15.74, 23.42) |
|  | 2012 | 10.61 (9.61, 11.86) | 9.12 (8.2, 10.63) | 9.67 (8.93, 10.83) | 7.94 (6.45, 9.63) | 13.59 (11.53, 16.13) | 19.78 (15.97, 23.76) |
|  | 2013 | 10.56 (9.53, 11.97) | 8.94 (8.06, 10.46) | 9.51 (8.76, 10.67) | 7.99 (6.53, 9.52) | 13.8 (11.65, 16.3) | 19.94 (16.02, 23.65) |
|  | 2014 | 10.53 (9.52, 11.95) | 8.61 (7.71, 10.08) | 9.43 (8.66, 10.69) | 8.02 (6.56, 9.63) | 14.12 (11.96, 16.52) | 20.16 (16.39, 23.96) |
|  | 2015 | 10.63 (9.62, 12.05) | 8.4 (7.49, 9.85) | 9.49 (8.73, 10.73) | 8.02 (6.59, 9.71) | 14.84 (12.47, 17.11) | 20.25 (16.48, 24.19) |
|  | 2016 | 10.57 (9.54, 12.01) | 8.25 (7.37, 9.77) | 9.31 (8.53, 10.6) | 7.99 (6.57, 9.69) | 15.03 (12.7, 17.66) | 20.38 (16.53, 24.33) |
|  | 2017 | 10.51 (9.46, 11.97) | 8.15 (7.27, 9.58) | 9.14 (8.32, 10.48) | 7.96 (6.56, 9.74) | 15.11 (12.61, 17.8) | 20.53 (16.79, 24.6) |
|  | 2018 | 10.57 (9.48, 12.07) | 8.21 (7.31, 9.7) | 9.18 (8.23, 10.62) | 7.99 (6.48, 9.73) | 15.26 (12.59, 18.17) | 20.87 (17.06, 25.08) |
|  | 2019 | 10.63 (9.47, 12.13) | 8.29 (7.35, 9.81) | 9.16 (8.21, 10.52) | 7.99 (6.45, 9.86) | 15.41 (12.55, 18.49) | 21.08 (17.3, 25.25) |
| male | 1990 | 14.8 (12.86, 16.02) | 27.27 (23.77, 30.34) | 18.1 (15.16, 20.84) | 5.61 (4.24, 6.92) | 5.43 (3.77, 7.14) | 5.46 (3.19, 7.73) |
|  | 1991 | 14.78 (12.91, 15.99) | 27.28 (23.78, 30.51) | 18.15 (15.44, 20.88) | 5.63 (4.3, 6.92) | 5.43 (3.77, 7.12) | 5.48 (3.24, 7.73) |
|  | 1992 | 14.8 (13.06, 16.12) | 27.31 (23.68, 30.43) | 18.35 (15.95, 21.07) | 5.63 (4.34, 6.83) | 5.42 (3.79, 7.08) | 5.5 (3.18, 7.73) |
|  | 1993 | 14.96 (13.35, 16.22) | 27.51 (23.99, 30.95) | 18.8 (17, 21.12) | 5.66 (4.41, 6.75) | 5.5 (3.93, 7.01) | 5.55 (3.31, 7.83) |
|  | 1994 | 14.95 (13.38, 16.32) | 27.44 (23.84, 30.73) | 18.91 (17.26, 21.2) | 5.67 (4.43, 6.69) | 5.54 (3.98, 7.09) | 5.58 (3.37, 7.79) |
|  | 1995 | 14.87 (13.3, 16.24) | 27.3 (23.92, 30.8) | 18.78 (17.25, 21.06) | 5.69 (4.49, 6.67) | 5.54 (4.02, 6.97) | 5.59 (3.38, 7.7) |
|  | 1996 | 14.71 (13.14, 15.95) | 26.9 (23.71, 30.34) | 18.51 (16.89, 20.63) | 5.77 (4.65, 6.72) | 5.63 (4.07, 7.07) | 5.62 (3.44, 7.79) |
|  | 1997 | 14.62 (12.99, 15.75) | 26.59 (23.45, 29.87) | 18.39 (16.57, 20.57) | 5.82 (4.62, 6.69) | 5.82 (4.27, 7.36) | 5.71 (3.5, 7.87) |
|  | 1998 | 14.64 (12.99, 15.9) | 26.59 (23.51, 30) | 18.51 (16.64, 20.35) | 5.87 (4.7, 6.7) | 5.85 (4.34, 7.29) | 5.74 (3.58, 7.81) |
|  | 1999 | 14.76 (13.16, 16.04) | 26.67 (23.78, 30.3) | 19 (17.4, 20.76) | 5.92 (4.75, 6.73) | 5.77 (4.37, 7.09) | 5.74 (3.65, 7.83) |
|  | 2000 | 14.79 (13.21, 16.12) | 26.53 (23.77, 30.24) | 19.19 (17.66, 20.88) | 6.02 (4.85, 6.92) | 5.82 (4.39, 7.15) | 5.81 (3.71, 7.96) |
|  | 2001 | 14.89 (13.4, 16.27) | 26.74 (23.97, 30.53) | 19.26 (17.68, 20.95) | 6.14 (5.01, 7.04) | 5.9 (4.56, 7.15) | 5.89 (3.82, 8.05) |
|  | 2002 | 15.01 (13.48, 16.45) | 26.79 (24.12, 30.79) | 19.61 (18.03, 21.47) | 6.24 (5.14, 7.34) | 5.95 (4.66, 7.23) | 5.96 (3.95, 8.04) |
|  | 2003 | 14.99 (13.54, 16.65) | 26.53 (23.94, 30.7) | 19.76 (18.31, 21.82) | 6.33 (5.24, 7.32) | 5.95 (4.72, 7.13) | 6.05 (3.99, 8.13) |
|  | 2004 | 14.75 (13.31, 16.24) | 25.76 (23.16, 29.71) | 19.7 (18.23, 21.68) | 6.4 (5.32, 7.53) | 5.85 (4.69, 6.89) | 6.03 (4.06, 8.05) |
|  | 2005 | 14.71 (13.35, 16.41) | 25.32 (22.93, 29.43) | 19.87 (18.28, 22.02) | 6.5 (5.32, 7.64) | 5.94 (4.75, 6.97) | 6.04 (4.07, 7.99) |
|  | 2006 | 14.48 (13.14, 16.19) | 24.71 (22.42, 28.76) | 19.5 (18.07, 21.44) | 6.6 (5.53, 7.75) | 6.04 (4.93, 7.09) | 6.03 (4.07, 8.08) |
|  | 2007 | 14.38 (13.05, 16.05) | 24.16 (21.92, 28.56) | 19.44 (17.98, 21.33) | 6.76 (5.64, 7.91) | 6.07 (4.96, 7.26) | 6.05 (4.19, 8) |
|  | 2008 | 14.38 (13.05, 16.25) | 23.77 (21.56, 28.49) | 19.53 (18.01, 21.59) | 7.05 (5.86, 8.37) | 6.16 (5.06, 7.38) | 6.05 (4.18, 7.98) |
|  | 2009 | 14.2 (12.87, 16.17) | 23.21 (21.12, 28.16) | 19.24 (17.7, 21.29) | 7.24 (6.07, 8.59) | 6.09 (5.07, 7.28) | 6.06 (4.18, 7.96) |
|  | 2010 | 14.08 (12.81, 16.14) | 22.65 (20.64, 27.62) | 19.17 (17.72, 21.31) | 7.45 (6.23, 8.86) | 6 (5.05, 7.19) | 6.01 (4.22, 7.92) |
|  | 2011 | 13.95 (12.66, 15.97) | 22.1 (20.14, 27.14) | 18.85 (17.38, 20.94) | 7.64 (6.27, 9.11) | 6.17 (5.13, 7.43) | 6.12 (4.26, 7.98) |
|  | 2012 | 13.78 (12.54, 15.89) | 21.62 (19.64, 26.75) | 18.57 (17.16, 20.79) | 7.76 (6.41, 9.35) | 6.15 (5.17, 7.49) | 6.11 (4.22, 7.95) |
|  | 2013 | 13.6 (12.35, 15.7) | 21.08 (19.13, 26.47) | 18.28 (16.8, 20.44) | 7.84 (6.5, 9.42) | 6.18 (5.24, 7.64) | 6.15 (4.3, 7.89) |
|  | 2014 | 13.4 (12.16, 15.63) | 20.43 (18.5, 26.07) | 18.06 (16.59, 20.23) | 7.95 (6.56, 9.57) | 6.22 (5.23, 7.63) | 6.11 (4.24, 7.89) |
|  | 2015 | 13.3 (12.1, 15.54) | 19.94 (18, 25.78) | 18.02 (16.62, 20.33) | 7.99 (6.55, 9.46) | 6.36 (5.4, 7.89) | 6.19 (4.28, 7.94) |
|  | 2016 | 13.15 (11.91, 15.41) | 19.69 (17.7, 25.62) | 17.69 (16.29, 20.13) | 8 (6.56, 9.58) | 6.43 (5.45, 7.95) | 6.2 (4.31, 7.95) |
|  | 2017 | 13.04 (11.81, 15.34) | 19.72 (17.71, 25.46) | 17.33 (15.88, 19.59) | 8.02 (6.55, 9.78) | 6.47 (5.46, 8.14) | 6.22 (4.3, 7.98) |
|  | 2018 | 13.05 (11.79, 15.29) | 19.95 (17.85, 25.46) | 17.19 (15.53, 19.58) | 8.05 (6.62, 9.79) | 6.55 (5.48, 8.21) | 6.28 (4.37, 8.08) |
|  | 2019 | 13.01 (11.68, 15.29) | 20.04 (17.85, 25.45) | 17.08 (15.35, 19.42) | 8.05 (6.53, 9.82) | 6.6 (5.5, 8.45) | 6.31 (4.35, 8.09) |

DALY, disability-adjusted life year; SDI, socio-demographic index.

**Supplementary table S4. Incidence cases of 204 countries and territories in 2019 (from high to low).**

| Countries and Territories | Incident cases (×10^2^, 95% UI) |
| --- | --- |
| United States of America | 183.19 (152.66, 228.91) |
| China | 159.1 (130.45, 194.43) |
| India | 76.73 (63.3, 91.63) |
| Germany | 67.3 (51.63, 95.4) |
| Russian Federation | 52.35 (44.38, 61.19) |
| Italy | 47.48 (37.91, 57.97) |
| France | 40.65 (30.55, 53.76) |
| United Kingdom | 33.21 (26.81, 43.62) |
| Poland | 27.51 (21.59, 39.04) |
| Spain | 24.74 (19.04, 31.79) |
| Canada | 24.03 (17.74, 32.1) |
| Ukraine | 14.41 (11.61, 17.82) |
| Turkey | 13.12 (9.66, 20.53) |
| Pakistan | 12.39 (9.84, 16.19) |
| Brazil | 12.29 (10.82, 14.35) |
| Australia | 12.19 (8.98, 16.65) |
| Japan | 9.72 (7.68, 13.19) |
| Sweden | 8.35 (6.89, 10.07) |
| Czechia | 8.02 (6.33, 9.99) |
| South Africa | 7.39 (6.17, 8.63) |
| Hungary | 6.94 (5.62, 8.67) |
| Greece | 6.83 (5.23, 9.25) |
| Portugal | 6.76 (5.07, 8.68) |
| Bangladesh | 6.6 (4.73, 8.98) |
| Austria | 6.42 (4.94, 8.47) |
| Romania | 6.14 (4.94, 7.88) |
| Nigeria | 5.97 (4.25, 7.6) |
| Belgium | 5.81 (4.4, 7.54) |
| Israel | 5.3 (3.93, 7.16) |
| Indonesia | 5.11 (3.71, 7.1) |
| Ethiopia | 4.8 (3.05, 6.89) |
| Viet Nam | 4.68 (3.1, 7.16) |
| Denmark | 4.47 (3.35, 6.03) |
| Switzerland | 4.47 (3.36, 5.87) |
| Iran (Islamic Republic of) | 4.37 (2.62, 5.06) |
| Mexico | 4.34 (3.57, 5.49) |
| Croatia | 4.02 (3.03, 5.42) |
| Netherlands | 3.88 (2.7, 8.37) |
| Thailand | 3.74 (2.37, 5.22) |
| Belarus | 3.64 (2.69, 4.87) |
| Argentina | 3.36 (2.56, 4.5) |
| New Zealand | 2.99 (2.32, 3.7) |
| Algeria | 2.98 (2.11, 3.91) |
| Slovakia | 2.96 (2.23, 4) |
| Ireland | 2.84 (2.09, 3.72) |
| Norway | 2.82 (2.33, 3.45) |
| Malaysia | 2.79 (1.99, 4.33) |
| Finland | 2.66 (1.95, 3.5) |
| Republic of Korea | 2.52 (1.67, 3.27) |
| Colombia | 2.44 (1.79, 3.23) |
| Saudi Arabia | 2.35 (1.66, 3.25) |
| Cuba | 2.21 (1.72, 2.86) |
| Taiwan (Province of China) | 2.15 (1.5, 3.53) |
| Egypt | 2.01 (1.23, 3.37) |
| Bulgaria | 1.85 (1.37, 2.47) |
| Democratic Republic of the Congo | 1.83 (1.08, 2.79) |
| United Republic of Tanzania | 1.81 (1.42, 2.29) |
| Serbia | 1.72 (1.23, 2.29) |
| Slovenia | 1.71 (1.25, 2.33) |
| Chile | 1.71 (1.29, 2.25) |
| Nepal | 1.7 (1.22, 2.33) |
| Lithuania | 1.66 (1.28, 2.25) |
| Kazakhstan | 1.66 (1.3, 2.15) |
| Uzbekistan | 1.48 (1.05, 1.95) |
| Bosnia and Herzegovina | 1.44 (1.05, 1.93) |
| Venezuela (Bolivarian Republic of) | 1.37 (1.01, 1.84) |
| Latvia | 1.36 (1.06, 1.74) |
| Kenya | 1.27 (0.98, 1.74) |
| Philippines | 1.18 (0.93, 1.54) |
| Syrian Arab Republic | 1.14 (0.72, 1.7) |
| Peru | 1.11 (0.75, 1.56) |
| Zimbabwe | 1.03 (0.74, 1.38) |
| Uruguay | 1.03 (0.8, 1.31) |
| North Macedonia | 1.02 (0.75, 1.35) |
| Myanmar | 0.92 (0.65, 1.29) |
| Mozambique | 0.9 (0.64, 1.29) |
| Democratic People's Republic of Korea | 0.88 (0.57, 1.29) |
| Cameroon | 0.8 (0.58, 1.11) |
| Cyprus | 0.75 (0.6, 0.92) |
| Iraq | 0.71 (0.48, 1.03) |
| Uganda | 0.69 (0.51, 0.88) |
| Angola | 0.67 (0.44, 0.97) |
| Ghana | 0.67 (0.5, 0.86) |
| Morocco | 0.67 (0.38, 0.97) |
| Tunisia | 0.65 (0.43, 0.95) |
| Albania | 0.64 (0.39, 0.98) |
| Lebanon | 0.62 (0.38, 0.93) |
| Armenia | 0.6 (0.46, 0.81) |
| Malawi | 0.6 (0.44, 0.8) |
| Sudan | 0.59 (0.25, 0.93) |
| Madagascar | 0.57 (0.4, 0.79) |
| Zambia | 0.55 (0.4, 0.76) |
| Estonia | 0.55 (0.39, 0.72) |
| Ecuador | 0.53 (0.4, 0.7) |
| Côte d'Ivoire | 0.5 (0.36, 0.67) |
| Mali | 0.5 (0.38, 0.67) |
| Puerto Rico | 0.49 (0.36, 0.65) |
| Burkina Faso | 0.47 (0.35, 0.63) |
| Afghanistan | 0.44 (0.18, 0.73) |
| Rwanda | 0.44 (0.34, 0.57) |
| Senegal | 0.41 (0.31, 0.55) |
| Costa Rica | 0.39 (0.28, 0.54) |
| Yemen | 0.39 (0.16, 0.62) |
| Montenegro | 0.38 (0.3, 0.5) |
| Qatar | 0.37 (0.26, 0.53) |
| Niger | 0.35 (0.25, 0.48) |
| Somalia | 0.35 (0.18, 0.56) |
| Azerbaijan | 0.35 (0.26, 0.5) |
| Guinea | 0.34 (0.24, 0.45) |
| Bolivia (Plurinational State of) | 0.34 (0.24, 0.47) |
| Paraguay | 0.34 (0.24, 0.46) |
| Singapore | 0.33 (0.24, 0.43) |
| Honduras | 0.31 (0.21, 0.42) |
| Cambodia | 0.31 (0.21, 0.41) |
| Georgia | 0.31 (0.2, 0.41) |
| Botswana | 0.3 (0.19, 0.44) |
| United Arab Emirates | 0.27 (0.15, 0.46) |
| Republic of Moldova | 0.27 (0.22, 0.33) |
| Dominican Republic | 0.27 (0.19, 0.39) |
| Sri Lanka | 0.25 (0.17, 0.34) |
| Benin | 0.25 (0.19, 0.34) |
| Burundi | 0.24 (0.17, 0.34) |
| Chad | 0.24 (0.18, 0.33) |
| Nicaragua | 0.23 (0.18, 0.3) |
| Libya | 0.23 (0.13, 0.34) |
| Luxembourg | 0.23 (0.18, 0.3) |
| Lesotho | 0.21 (0.14, 0.3) |
| Haiti | 0.2 (0.12, 0.33) |
| Eritrea | 0.2 (0.14, 0.29) |
| Panama | 0.2 (0.15, 0.28) |
| Congo | 0.2 (0.14, 0.29) |
| South Sudan | 0.19 (0.12, 0.28) |
| Sierra Leone | 0.18 (0.13, 0.25) |
| Togo | 0.18 (0.13, 0.25) |
| Guatemala | 0.17 (0.13, 0.22) |
| Kuwait | 0.17 (0.13, 0.24) |
| Namibia | 0.17 (0.12, 0.24) |
| Tajikistan | 0.17 (0.12, 0.24) |
| Turkmenistan | 0.17 (0.13, 0.24) |
| Jamaica | 0.15 (0.11, 0.2) |
| Iceland | 0.15 (0.11, 0.18) |
| Malta | 0.15 (0.11, 0.19) |
| Bahrain | 0.14 (0.11, 0.19) |
| El Salvador | 0.13 (0.09, 0.18) |
| Kyrgyzstan | 0.13 (0.1, 0.18) |
| Mauritania | 0.12 (0.09, 0.16) |
| Central African Republic | 0.11 (0.07, 0.17) |
| Palestine | 0.11 (0.08, 0.14) |
| Oman | 0.1 (0.06, 0.15) |
| Liberia | 0.1 (0.07, 0.14) |
| Brunei Darussalam | 0.09 (0.08, 0.12) |
| Eswatini | 0.09 (0.06, 0.13) |
| Gabon | 0.09 (0.06, 0.13) |
| Lao People's Democratic Republic | 0.08 (0.06, 0.11) |
| Jordan | 0.07 (0.05, 0.09) |
| Mongolia | 0.07 (0.05, 0.1) |
| Trinidad and Tobago | 0.06 (0.04, 0.08) |
| Andorra | 0.06 (0.04, 0.08) |
| Gambia | 0.06 (0.04, 0.08) |
| Bhutan | 0.05 (0.03, 0.06) |
| Comoros | 0.04 (0.03, 0.05) |
| Djibouti | 0.04 (0.03, 0.06) |
| Cabo Verde | 0.04 (0.03, 0.05) |
| United States Virgin Islands | 0.04 (0.03, 0.05) |
| Barbados | 0.04 (0.03, 0.05) |
| Guinea-Bissau | 0.04 (0.03, 0.06) |
| Equatorial Guinea | 0.04 (0.02, 0.07) |
| Seychelles | 0.04 (0.04, 0.05) |
| Bahamas | 0.03 (0.03, 0.04) |
| Monaco | 0.03 (0.01, 0.05) |
| Guyana | 0.03 (0.02, 0.03) |
| Saint Lucia | 0.02 (0.01, 0.02) |
| Suriname | 0.02 (0.02, 0.02) |
| Papua New Guinea | 0.02 (0.01, 0.04) |
| Mauritius | 0.02 (0.02, 0.03) |
| Timor-Leste | 0.01 (0.01, 0.02) |
| Saint Kitts and Nevis | 0.01 (0, 0.01) |
| Bermuda | 0.01 (0.01, 0.02) |
| Saint Vincent and the Grenadines | 0.01 (0.01, 0.01) |
| Sao Tome and Principe | 0.01 (0, 0.01) |
| Fiji | 0.01 (0.01, 0.02) |
| Greenland | 0.01 (0.01, 0.02) |
| Belize | 0.01 (0.01, 0.01) |
| Maldives | 0.01 (0.01, 0.01) |
| Grenada | 0.01 (0.01, 0.01) |
| Tonga | 0 (0, 0) |
| Vanuatu | 0 (0, 0) |
| Palau | 0 (0, 0) |
| American Samoa | 0 (0, 0) |
| San Marino | 0 (0, 0) |
| Tokelau | 0 (0, 0) |
| Antigua and Barbuda | 0 (0, 0) |
| Kiribati | 0 (0, 0) |
| Guam | 0 (0, 0) |
| Tuvalu | 0 (0, 0) |
| Cook Islands | 0 (0, 0) |
| Nauru | 0 (0, 0) |
| Marshall Islands | 0 (0, 0) |
| Dominica | 0 (0, 0.01) |
| Micronesia (Federated States of) | 0 (0, 0) |
| Samoa | 0 (0, 0) |
| Solomon Islands | 0 (0, 0.01) |
| Niue | 0 (0, 0) |
| Northern Mariana Islands | 0 (0, 0) |

UI: uncertainty interval.

**Supplementary table S5. Death cases of 204 countries and territories in 2019 (from high to low).**

| Countries and Territories | Incident cases (×10^2^, 95% UI) |
| --- | --- |
| India | 61.96 (50.77, 74.94) |
| United States of America | 59.42 (52.53, 72.18) |
| China | 47.12 (38.86, 58.22) |
| Germany | 24.4 (20.62, 31.61) |
| Russian Federation | 21.92 (18.47, 25.28) |
| Italy | 16.17 (13.62, 18.34) |
| France | 15.85 (12.81, 19.36) |
| United Kingdom | 13.22 (11.29, 16.73) |
| Poland | 11.45 (9.01, 15.54) |
| Pakistan | 10.02 (8.05, 12.69) |
| Spain | 8.61 (6.99, 10.23) |
| Brazil | 8.38 (7.29, 9.79) |
| Canada | 7.53 (6.28, 9.05) |
| Turkey | 6.55 (4.78, 10.21) |
| Ukraine | 6.27 (5.19, 7.71) |
| South Africa | 5.68 (4.48, 6.52) |
| Bangladesh | 5.3 (3.82, 7.26) |
| Nigeria | 5.12 (3.61, 6.44) |
| Ethiopia | 4.57 (2.87, 6.59) |
| Indonesia | 4.02 (2.97, 5.55) |
| Australia | 3.98 (3.29, 5.15) |
| Japan | 3.45 (2.76, 4.7) |
| Sweden | 3.01 (2.57, 3.44) |
| Viet Nam | 2.96 (1.92, 4.63) |
| Czechia | 2.92 (2.33, 3.58) |
| Hungary | 2.87 (2.33, 3.51) |
| Romania | 2.8 (2.25, 3.54) |
| Mexico | 2.73 (2.23, 3.42) |
| Greece | 2.66 (2.26, 3.35) |
| Portugal | 2.61 (2.15, 3.12) |
| Austria | 2.29 (1.9, 2.83) |
| Thailand | 2.26 (1.4, 3.14) |
| Argentina | 2.19 (1.84, 2.74) |
| Israel | 2.18 (1.82, 2.72) |
| Belgium | 2.17 (1.79, 2.58) |
| Iran (Islamic Republic of) | 1.86 (1.15, 2.14) |
| Democratic Republic of the Congo | 1.68 (1, 2.58) |
| United Republic of Tanzania | 1.65 (1.31, 2.09) |
| Malaysia | 1.63 (1.17, 2.52) |
| Algeria | 1.61 (1.21, 2.07) |
| Switzerland | 1.53 (1.26, 1.85) |
| Croatia | 1.49 (1.15, 1.94) |
| Denmark | 1.47 (1.21, 1.85) |
| Nepal | 1.44 (1.04, 1.96) |
| Colombia | 1.41 (1.04, 1.88) |
| Belarus | 1.4 (1.07, 1.85) |
| Kenya | 1.2 (0.93, 1.64) |
| Egypt | 1.15 (0.68, 1.94) |
| Slovakia | 1.14 (0.86, 1.5) |
| Netherlands | 1.12 (0.87, 2.45) |
| Cuba | 1.1 (0.87, 1.4) |
| New Zealand | 1 (0.8, 1.15) |
| Finland | 0.98 (0.81, 1.18) |
| Saudi Arabia | 0.94 (0.69, 1.3) |
| Bulgaria | 0.92 (0.68, 1.21) |
| Chile | 0.89 (0.75, 1.09) |
| Mozambique | 0.88 (0.62, 1.24) |
| Zimbabwe | 0.86 (0.62, 1.15) |
| Lithuania | 0.85 (0.65, 1.14) |
| Philippines | 0.85 (0.69, 1.1) |
| Venezuela (Bolivarian Republic of) | 0.83 (0.62, 1.11) |
| Ireland | 0.82 (0.66, 1.01) |
| Norway | 0.82 (0.71, 0.94) |
| Kazakhstan | 0.79 (0.61, 1.01) |
| Uzbekistan | 0.79 (0.56, 1.03) |
| Taiwan (Province of China) | 0.77 (0.55, 1.21) |
| Myanmar | 0.75 (0.54, 1.03) |
| Cameroon | 0.7 (0.5, 0.96) |
| Uruguay | 0.69 (0.55, 0.81) |
| Serbia | 0.69 (0.49, 0.9) |
| Bosnia and Herzegovina | 0.68 (0.51, 0.91) |
| Peru | 0.65 (0.44, 0.89) |
| Latvia | 0.65 (0.51, 0.81) |
| Uganda | 0.62 (0.46, 0.79) |
| Republic of Korea | 0.6 (0.39, 0.74) |
| Slovenia | 0.6 (0.43, 0.82) |
| Angola | 0.59 (0.39, 0.85) |
| Syrian Arab Republic | 0.58 (0.37, 0.9) |
| Ghana | 0.57 (0.43, 0.73) |
| Malawi | 0.55 (0.4, 0.72) |
| Democratic People's Republic of Korea | 0.52 (0.35, 0.74) |
| Madagascar | 0.52 (0.37, 0.72) |
| Zambia | 0.48 (0.36, 0.65) |
| Mali | 0.45 (0.34, 0.6) |
| Côte d'Ivoire | 0.45 (0.33, 0.6) |
| North Macedonia | 0.44 (0.33, 0.58) |
| Morocco | 0.44 (0.26, 0.64) |
| Burkina Faso | 0.43 (0.32, 0.57) |
| Sudan | 0.41 (0.18, 0.64) |
| Rwanda | 0.4 (0.3, 0.5) |
| Iraq | 0.38 (0.26, 0.56) |
| Ecuador | 0.38 (0.28, 0.49) |
| Senegal | 0.37 (0.28, 0.5) |
| Afghanistan | 0.36 (0.15, 0.6) |
| Somalia | 0.35 (0.18, 0.55) |
| Guinea | 0.32 (0.23, 0.42) |
| Niger | 0.32 (0.23, 0.45) |
| Armenia | 0.29 (0.23, 0.37) |
| Tunisia | 0.29 (0.19, 0.42) |
| Bolivia (Plurinational State of) | 0.29 (0.2, 0.39) |
| Albania | 0.28 (0.18, 0.42) |
| Yemen | 0.28 (0.12, 0.45) |
| Puerto Rico | 0.25 (0.19, 0.33) |
| Honduras | 0.25 (0.17, 0.33) |
| Cambodia | 0.24 (0.17, 0.32) |
| Paraguay | 0.24 (0.17, 0.32) |
| Costa Rica | 0.23 (0.16, 0.31) |
| Burundi | 0.23 (0.16, 0.32) |
| Cyprus | 0.23 (0.18, 0.28) |
| Chad | 0.23 (0.17, 0.31) |
| Benin | 0.23 (0.17, 0.31) |
| Lebanon | 0.22 (0.13, 0.34) |
| Estonia | 0.21 (0.16, 0.28) |
| Dominican Republic | 0.2 (0.14, 0.28) |
| Botswana | 0.2 (0.13, 0.29) |
| Azerbaijan | 0.2 (0.15, 0.28) |
| Eritrea | 0.19 (0.13, 0.26) |
| Lesotho | 0.19 (0.13, 0.27) |
| Georgia | 0.18 (0.12, 0.24) |
| Haiti | 0.18 (0.11, 0.31) |
| South Sudan | 0.18 (0.12, 0.27) |
| Congo | 0.18 (0.12, 0.25) |
| Sierra Leone | 0.16 (0.12, 0.23) |
| Togo | 0.16 (0.12, 0.22) |
| Montenegro | 0.15 (0.11, 0.19) |
| Republic of Moldova | 0.15 (0.13, 0.19) |
| Namibia | 0.13 (0.1, 0.18) |
| Guatemala | 0.13 (0.1, 0.16) |
| Panama | 0.13 (0.1, 0.18) |
| Nicaragua | 0.13 (0.1, 0.17) |
| United Arab Emirates | 0.13 (0.07, 0.23) |
| Sri Lanka | 0.12 (0.08, 0.16) |
| Libya | 0.12 (0.07, 0.18) |
| Tajikistan | 0.11 (0.08, 0.17) |
| Qatar | 0.11 (0.08, 0.15) |
| Central African Republic | 0.11 (0.07, 0.17) |
| Singapore | 0.1 (0.08, 0.12) |
| Jamaica | 0.1 (0.07, 0.13) |
| Turkmenistan | 0.1 (0.07, 0.13) |
| Mauritania | 0.1 (0.07, 0.13) |
| Liberia | 0.09 (0.06, 0.12) |
| El Salvador | 0.08 (0.06, 0.11) |
| Gabon | 0.08 (0.05, 0.11) |
| Eswatini | 0.08 (0.05, 0.11) |
| Kyrgyzstan | 0.07 (0.06, 0.1) |
| Lao People's Democratic Republic | 0.07 (0.05, 0.09) |
| Luxembourg | 0.07 (0.06, 0.09) |
| Kuwait | 0.06 (0.04, 0.08) |
| Gambia | 0.05 (0.04, 0.07) |
| Palestine | 0.05 (0.04, 0.07) |
| Malta | 0.05 (0.04, 0.07) |
| Bahrain | 0.05 (0.04, 0.07) |
| Brunei Darussalam | 0.04 (0.03, 0.05) |
| Iceland | 0.04 (0.03, 0.05) |
| Trinidad and Tobago | 0.04 (0.03, 0.05) |
| Djibouti | 0.04 (0.03, 0.05) |
| Bhutan | 0.04 (0.03, 0.05) |
| Guinea-Bissau | 0.04 (0.03, 0.05) |
| Mongolia | 0.04 (0.03, 0.06) |
| Oman | 0.04 (0.02, 0.05) |
| Jordan | 0.03 (0.02, 0.03) |
| Comoros | 0.03 (0.03, 0.04) |
| Seychelles | 0.03 (0.02, 0.03) |
| Equatorial Guinea | 0.03 (0.02, 0.05) |
| Cabo Verde | 0.03 (0.02, 0.03) |
| United States Virgin Islands | 0.02 (0.02, 0.03) |
| Andorra | 0.02 (0.01, 0.03) |
| Papua New Guinea | 0.02 (0.01, 0.03) |
| Barbados | 0.02 (0.02, 0.03) |
| Bahamas | 0.02 (0.02, 0.02) |
| Guyana | 0.02 (0.01, 0.03) |
| Suriname | 0.01 (0.01, 0.02) |
| Saint Vincent and the Grenadines | 0.01 (0.01, 0.01) |
| Maldives | 0.01 (0, 0.01) |
| Timor-Leste | 0.01 (0.01, 0.02) |
| Fiji | 0.01 (0.01, 0.01) |
| Saint Lucia | 0.01 (0.01, 0.02) |
| Greenland | 0.01 (0.01, 0.01) |
| Mauritius | 0.01 (0.01, 0.01) |
| Belize | 0.01 (0.01, 0.01) |
| Bermuda | 0.01 (0, 0.01) |
| Monaco | 0.01 (0, 0.02) |
| Vanuatu | 0 (0, 0) |
| Grenada | 0 (0, 0.01) |
| Dominica | 0 (0, 0) |
| Saint Kitts and Nevis | 0 (0, 0) |
| Kiribati | 0 (0, 0) |
| American Samoa | 0 (0, 0) |
| Solomon Islands | 0 (0, 0) |
| Tokelau | 0 (0, 0) |
| Micronesia (Federated States of) | 0 (0, 0) |
| Tuvalu | 0 (0, 0) |
| Antigua and Barbuda | 0 (0, 0) |
| San Marino | 0 (0, 0) |
| Tonga | 0 (0, 0) |
| Nauru | 0 (0, 0) |
| Cook Islands | 0 (0, 0) |
| Guam | 0 (0, 0) |
| Palau | 0 (0, 0) |
| Marshall Islands | 0 (0, 0) |
| Samoa | 0 (0, 0) |
| Northern Mariana Islands | 0 (0, 0) |
| Niue | 0 (0, 0) |
| Sao Tome and Principe | 0 (0, 0.01) |

UI: uncertainty interval.

**Supplementary table S6. The DALYs 204 countries and territories in 2019 (from high to low).**

| Countries and Territories | DALYs (×10^2^, 95% UI) |
| --- | --- |
| China | 1469.13 (1218.16, 1792.32) |
| India | 1397.69 (1142.67, 1707.53) |
| United States of America | 1046.63 (936.42, 1290.35) |
| Russian Federation | 506.94 (425.01, 590.27) |
| Germany | 407.58 (344.21, 537.21) |
| Pakistan | 278.27 (219.6, 359.68) |
| Italy | 259.56 (226.09, 301.73) |
| France | 236.62 (196.48, 291.15) |
| Poland | 231.56 (181.37, 325.89) |
| United Kingdom | 211.07 (186.67, 268) |
| Brazil | 164.87 (147.47, 193.74) |
| Ukraine | 157.46 (130.03, 193.7) |
| Turkey | 139.88 (102.63, 217.56) |
| Spain | 136.16 (114.2, 161.17) |
| Canada | 134.49 (112.06, 162.14) |
| South Africa | 131.16 (109.9, 153.45) |
| Nigeria | 120.56 (84.99, 154.77) |
| Bangladesh | 117.54 (84.42, 161.45) |
| Indonesia | 98.9 (72.88, 139.67) |
| Ethiopia | 95.08 (60.74, 135.06) |
| Australia | 68.05 (57.18, 89.39) |
| Romania | 59.46 (47.49, 76.24) |
| Viet Nam | 58.63 (39.38, 89.05) |
| Mexico | 58.6 (47.76, 74.93) |
| Japan | 57.64 (48.08, 78.71) |
| Hungary | 55.85 (44.85, 70.23) |
| Czechia | 55.64 (44.01, 69.74) |
| Iran (Islamic Republic of) | 53.34 (31.93, 61.08) |
| Sweden | 45.63 (39.83, 52.94) |
| Thailand | 43.23 (27.21, 61.36) |
| Portugal | 43.01 (35.7, 51.85) |
| Democratic Republic of the Congo | 42.68 (25.27, 65.13) |
| Greece | 42.3 (35.96, 55.37) |
| Argentina | 40.55 (34.46, 50.46) |
| Austria | 37.22 (31.2, 48.09) |
| United Republic of Tanzania | 36.89 (28.56, 47.71) |
| Israel | 35.89 (30.23, 46.18) |
| Algeria | 35.79 (25.75, 46.79) |
| Egypt | 35.18 (21.52, 60.03) |
| Belgium | 34.41 (28.79, 41.14) |
| Malaysia | 34.13 (24.84, 51.33) |
| Belarus | 34.05 (25.11, 45.33) |
| Nepal | 31.81 (22.68, 44.68) |
| Colombia | 28.8 (20.95, 38.56) |
| Kenya | 28.16 (21.45, 38.61) |
| Croatia | 27.08 (20.38, 36.21) |
| Saudi Arabia | 26.94 (18.9, 37.35) |
| Denmark | 25.52 (21.09, 32.43) |
| Uzbekistan | 24.36 (17.45, 31.94) |
| Zimbabwe | 24.32 (17.43, 33.04) |
| Switzerland | 24.19 (20.11, 29.34) |
| Philippines | 23.81 (19.26, 30.79) |
| Slovakia | 23.74 (17.88, 31.79) |
| Cuba | 22.11 (17.17, 28.7) |
| Kazakhstan | 21.4 (16.65, 27.75) |
| Netherlands | 20.49 (15.68, 45.47) |
| Bulgaria | 19.29 (14.13, 25.73) |
| Mozambique | 19 (13.03, 27.69) |
| Myanmar | 18.74 (13.01, 26.52) |
| Venezuela (Bolivarian Republic of) | 18.29 (13.33, 24.58) |
| Democratic People's Republic of Korea | 17.48 (10.87, 26.34) |
| New Zealand | 17.36 (13.9, 20.04) |
| Finland | 16.8 (13.63, 20.59) |
| Cameroon | 16.59 (12.01, 23.3) |
| Peru | 16.22 (10.94, 22.8) |
| Lithuania | 16.13 (12.21, 21.75) |
| Serbia | 15.98 (11.4, 21.27) |
| Chile | 15.84 (13.43, 19.4) |
| Angola | 15.49 (10.12, 22.85) |
| Syrian Arab Republic | 15.44 (9.89, 23.51) |
| Taiwan (Province of China) | 15.33 (10.73, 24.68) |
| Ireland | 15.11 (12.01, 18.48) |
| Republic of Korea | 15.07 (10.19, 18.67) |
| Bosnia and Herzegovina | 14.58 (10.46, 19.68) |
| Uganda | 14.44 (10.48, 18.91) |
| Norway | 14.42 (12.7, 16.66) |
| Malawi | 13.34 (9.58, 18.05) |
| Madagascar | 13.29 (9.26, 18.72) |
| Ghana | 12.84 (9.68, 16.75) |
| Afghanistan | 12.71 (5.29, 21.48) |
| Latvia | 12.48 (9.75, 15.62) |
| Sudan | 12.47 (5.34, 19.89) |
| Zambia | 11.53 (8.47, 15.99) |
| Morocco | 11.51 (6.6, 17.03) |
| Uruguay | 11.2 (9.22, 12.99) |
| Mali | 11.14 (8.32, 15.13) |
| Iraq | 10.93 (7.41, 15.9) |
| Slovenia | 10.67 (7.73, 14.4) |
| Côte d'Ivoire | 10.58 (7.69, 14.4) |
| North Macedonia | 10.14 (7.34, 13.49) |
| Burkina Faso | 10.02 (7.31, 13.6) |
| Rwanda | 9.01 (6.81, 11.97) |
| Yemen | 8.85 (3.7, 14.29) |
| Somalia | 8.67 (4.62, 14.03) |
| Senegal | 8.51 (6.25, 11.47) |
| Ecuador | 7.89 (5.81, 10.39) |
| Niger | 7.87 (5.54, 10.74) |
| Guinea | 7.69 (5.49, 10.14) |
| Armenia | 7.15 (5.57, 9.82) |
| Tunisia | 7.04 (4.68, 10.24) |
| Bolivia (Plurinational State of) | 6.36 (4.4, 8.61) |
| Albania | 6.23 (3.73, 9.63) |
| Cambodia | 6.12 (4.29, 8.17) |
| Burundi | 5.59 (3.76, 7.95) |
| Azerbaijan | 5.59 (4.15, 7.86) |
| Honduras | 5.5 (3.66, 7.27) |
| Chad | 5.47 (3.92, 7.52) |
| Botswana | 5.35 (3.39, 7.79) |
| Benin | 5.27 (3.83, 7.3) |
| Lebanon | 5.2 (3.21, 7.85) |
| Paraguay | 5.05 (3.59, 6.84) |
| Lesotho | 4.84 (3.06, 6.98) |
| Eritrea | 4.7 (3.19, 6.68) |
| Haiti | 4.66 (2.89, 7.69) |
| United Arab Emirates | 4.59 (2.68, 7.82) |
| Congo | 4.55 (3.07, 6.74) |
| Dominican Republic | 4.52 (3.11, 6.5) |
| Cyprus | 4.34 (3.48, 5.34) |
| Puerto Rico | 4.28 (3.14, 5.71) |
| Georgia | 4.25 (2.63, 5.69) |
| South Sudan | 4.19 (2.64, 6.39) |
| Estonia | 4.16 (2.95, 5.41) |
| Costa Rica | 4.06 (2.92, 5.5) |
| Togo | 3.81 (2.74, 5.25) |
| Sierra Leone | 3.73 (2.67, 5.21) |
| Tajikistan | 3.44 (2.39, 5.02) |
| Nicaragua | 3.44 (2.64, 4.48) |
| Libya | 3.43 (1.98, 4.99) |
| Guatemala | 3.37 (2.51, 4.37) |
| Republic of Moldova | 3.36 (2.75, 4.11) |
| Montenegro | 3.2 (2.47, 4.15) |
| Namibia | 3.18 (2.28, 4.54) |
| Qatar | 3.14 (2.17, 4.43) |
| Central African Republic | 3.03 (1.84, 4.76) |
| Sri Lanka | 2.97 (2.11, 4.13) |
| Turkmenistan | 2.86 (2.1, 3.9) |
| Panama | 2.47 (1.8, 3.5) |
| Mauritania | 2.28 (1.59, 3.14) |
| Kyrgyzstan | 2.15 (1.72, 2.98) |
| Jamaica | 2.13 (1.49, 2.83) |
| Singapore | 2.13 (1.55, 2.57) |
| Liberia | 2.02 (1.38, 2.89) |
| Eswatini | 1.94 (1.25, 2.85) |
| El Salvador | 1.88 (1.3, 2.53) |
| Lao People's Democratic Republic | 1.79 (1.25, 2.51) |
| Gabon | 1.75 (1.15, 2.56) |
| Kuwait | 1.55 (1.13, 2.11) |
| Palestine | 1.54 (1.15, 2.05) |
| Mongolia | 1.33 (0.92, 1.9) |
| Bahrain | 1.33 (0.99, 1.75) |
| Luxembourg | 1.32 (1.07, 1.63) |
| Gambia | 1.31 (0.95, 1.71) |
| Oman | 1.1 (0.63, 1.58) |
| Brunei Darussalam | 1.05 (0.86, 1.28) |
| Guinea-Bissau | 0.93 (0.66, 1.27) |
| Trinidad and Tobago | 0.91 (0.63, 1.24) |
| Malta | 0.9 (0.7, 1.17) |
| Djibouti | 0.87 (0.59, 1.27) |
| Jordan | 0.82 (0.61, 1.05) |
| Equatorial Guinea | 0.79 (0.43, 1.35) |
| Bhutan | 0.77 (0.55, 1.03) |
| Iceland | 0.74 (0.58, 0.93) |
| Comoros | 0.72 (0.54, 0.96) |
| Papua New Guinea | 0.64 (0.4, 0.98) |
| Seychelles | 0.61 (0.51, 0.73) |
| Cabo Verde | 0.54 (0.43, 0.7) |
| Guyana | 0.51 (0.37, 0.67) |
| Bahamas | 0.48 (0.39, 0.6) |
| United States Virgin Islands | 0.47 (0.37, 0.6) |
| Barbados | 0.46 (0.37, 0.57) |
| Suriname | 0.34 (0.27, 0.44) |
| Andorra | 0.33 (0.23, 0.46) |
| Timor-Leste | 0.3 (0.19, 0.45) |
| Mauritius | 0.29 (0.22, 0.37) |
| Saint Lucia | 0.27 (0.22, 0.34) |
| Fiji | 0.23 (0.15, 0.35) |
| Saint Vincent and the Grenadines | 0.2 (0.16, 0.24) |
| Greenland | 0.19 (0.15, 0.24) |
| Monaco | 0.18 (0.06, 0.26) |
| Belize | 0.17 (0.14, 0.21) |
| Maldives | 0.12 (0.08, 0.17) |
| Bermuda | 0.11 (0.09, 0.15) |
| Sao Tome and Principe | 0.11 (0.08, 0.15) |
| Grenada | 0.11 (0.09, 0.13) |
| Solomon Islands | 0.1 (0.06, 0.16) |
| Dominica | 0.08 (0.06, 0.1) |
| Saint Kitts and Nevis | 0.08 (0.06, 0.1) |
| Samoa | 0.04 (0.02, 0.05) |
| Vanuatu | 0.03 (0.02, 0.05) |
| Kiribati | 0.02 (0.01, 0.03) |
| Micronesia (Federated States of) | 0.02 (0.01, 0.03) |
| Guam | 0.02 (0.02, 0.03) |
| Antigua and Barbuda | 0.02 (0.02, 0.03) |
| San Marino | 0.01 (0.01, 0.02) |
| Marshall Islands | 0.01 (0.01, 0.01) |
| Tonga | 0.01 (0.01, 0.01) |
| American Samoa | 0.01 (0.01, 0.01) |
| Northern Mariana Islands | 0.01 (0.01, 0.01) |
| Palau | 0 (0, 0.01) |
| Tuvalu | 0 (0, 0) |
| Cook Islands | 0 (0, 0) |
| Tokelau | 0 (0, 0) |
| Nauru | 0 (0, 0) |
| Niue | 0 (0, 0) |

DALY: disability-adjusted life year, UI: uncertainty interval.

**Supplementary table S7. Age-standardized incidence rates of 204 countries and territories in 2019 (from high to low).**

| Countries and Territories | ASIRs per 100,000 (95% UI) |
| --- | --- |
| Qatar | 6.57 (4.64, 9.31) |
| Israel | 4.53 (3.34, 6.14) |
| Croatia | 4.47 (3.34, 6.05) |
|  |  |
|  |  |
|  |  |
| Andorra | 4.16 (2.92, 5.8) |
| Seychelles | 4.02 (3.36, 4.84) |
| Poland | 3.93 (3.07, 5.59) |
| Slovenia | 3.92 (2.84, 5.36) |
| Cyprus | 3.84 (3.12, 4.68) |
| Montenegro | 3.83 (3, 4.99) |
| Denmark | 3.77 (2.81, 5.06) |
| Sweden | 3.75 (3.09, 4.56) |
| Ireland | 3.75 (2.75, 4.93) |
| New Zealand | 3.73 (2.88, 4.62) |
| Czechia | 3.7 (2.93, 4.61) |
| Hungary | 3.58 (2.88, 4.49) |
| Germany | 3.51 (2.68, 5.04) |
| Austria | 3.48 (2.68, 4.68) |
| Canada | 3.43 (2.52, 4.57) |
| Monaco | 3.41 (1.23, 4.96) |
| Latvia | 3.38 (2.65, 4.34) |
| Italy | 3.27 (2.63, 4.01) |
| United States of America | 3.16 (2.63, 3.95) |
| Slovakia | 3.14 (2.38, 4.23) |
| North Macedonia | 3.11 (2.31, 4.07) |
| Brunei Darussalam | 3.05 (2.54, 3.67) |
| Norway | 2.94 (2.4, 3.6) |
| Greece | 2.89 (2.19, 3.96) |
| Lithuania | 2.89 (2.22, 3.94) |
| France | 2.85 (2.1, 3.82) |
| Australia | 2.85 (2.1, 3.92) |
| Portugal | 2.79 (2.1, 3.6) |
| Iceland | 2.66 (2.03, 3.32) |
| Spain | 2.53 (1.94, 3.31) |
| United Kingdom | 2.53 (2.05, 3.3) |
| Switzerland | 2.48 (1.86, 3.29) |
| Belgium | 2.48 (1.88, 3.24) |
| Bosnia and Herzegovina | 2.36 (1.74, 3.18) |
| Luxembourg | 2.35 (1.83, 2.97) |
| Belarus | 2.3 (1.7, 3.06) |
| Botswana | 2.29 (1.52, 3.27) |
| Russian Federation | 2.22 (1.88, 2.6) |
| Greenland | 2.2 (1.74, 2.76) |
| Finland | 2.15 (1.57, 2.83) |
| Estonia | 2.11 (1.48, 2.77) |
| Ukraine | 1.93 (1.56, 2.39) |
| United States Virgin Islands | 1.9 (1.52, 2.41) |
| Bahrain | 1.86 (1.43, 2.44) |
| Lesotho | 1.84 (1.19, 2.6) |
| Uruguay | 1.82 (1.39, 2.31) |
| Eswatini | 1.74 (1.17, 2.46) |
| South Africa | 1.72 (1.4, 2) |
| Romania | 1.71 (1.37, 2.19) |
| Malta | 1.53 (1.18, 1.98) |
| Turkey | 1.51 (1.11, 2.36) |
| Albania | 1.5 (0.91, 2.3) |
| Zimbabwe | 1.46 (1.07, 1.97) |
| Armenia | 1.45 (1.13, 1.94) |
| Saudi Arabia | 1.41 (1.05, 1.95) |
| Ethiopia | 1.35 (0.84, 1.95) |
| Bulgaria | 1.32 (0.98, 1.77) |
| Namibia | 1.25 (0.92, 1.73) |
| Lebanon | 1.19 (0.73, 1.79) |
| Cuba | 1.17 (0.91, 1.52) |
| Serbia | 1.16 (0.83, 1.55) |
| Netherlands | 1.14 (0.79, 2.45) |
| Pakistan | 1.13 (0.91, 1.43) |
| Malaysia | 1.12 (0.8, 1.74) |
| Bermuda | 1.03 (0.8, 1.34) |
| Mozambique | 1.03 (0.74, 1.43) |
| Cabo Verde | 0.97 (0.77, 1.26) |
| Zambia | 0.94 (0.71, 1.27) |
| Gabon | 0.94 (0.63, 1.32) |
| Algeria | 0.93 (0.68, 1.2) |
| Kazakhstan | 0.93 (0.73, 1.2) |
| Eritrea | 0.93 (0.66, 1.26) |
| Equatorial Guinea | 0.93 (0.54, 1.51) |
| Syrian Arab Republic | 0.91 (0.58, 1.36) |
| Saint Vincent and the Grenadines | 0.9 (0.75, 1.09) |
| Malawi | 0.89 (0.66, 1.17) |
| Bhutan | 0.89 (0.64, 1.17) |
| Saint Lucia | 0.86 (0.69, 1.07) |
| Rwanda | 0.85 (0.66, 1.08) |
| Bahamas | 0.84 (0.69, 1.03) |
| Congo | 0.84 (0.6, 1.17) |
| Saint Kitts and Nevis | 0.84 (0.67, 1.09) |
| China | 0.83 (0.68, 1.01) |
| United Republic of Tanzania | 0.83 (0.65, 1.04) |
| Nepal | 0.82 (0.6, 1.11) |
| Djibouti | 0.82 (0.61, 1.12) |
| Costa Rica | 0.78 (0.57, 1.06) |
| Comoros | 0.78 (0.6, 1.02) |
| Barbados | 0.77 (0.6, 0.94) |
| Nigeria | 0.75 (0.54, 0.93) |
| Kuwait | 0.74 (0.53, 1.05) |
| Cameroon | 0.73 (0.53, 1) |
| India | 0.71 (0.59, 0.85) |
| Chile | 0.71 (0.54, 0.94) |
| Angola | 0.68 (0.46, 0.95) |
| Puerto Rico | 0.68 (0.49, 0.9) |
| Uzbekistan | 0.67 (0.47, 0.87) |
| Kenya | 0.66 (0.51, 0.91) |
| Gambia | 0.66 (0.48, 0.87) |
| Somalia | 0.65 (0.34, 1.01) |
| United Arab Emirates | 0.64 (0.33, 1.2) |
| Paraguay | 0.64 (0.45, 0.85) |
| Guinea | 0.64 (0.46, 0.84) |
| Guinea-Bissau | 0.64 (0.45, 0.85) |
| Grenada | 0.63 (0.52, 0.75) |
| Burundi | 0.62 (0.43, 0.86) |
| Argentina | 0.62 (0.47, 0.83) |
| Madagascar | 0.61 (0.44, 0.83) |
| Central African Republic | 0.6 (0.36, 0.94) |
| Mauritania | 0.6 (0.44, 0.8) |
| Mali | 0.6 (0.46, 0.8) |
| Sao Tome and Principe | 0.59 (0.43, 0.79) |
| Senegal | 0.58 (0.43, 0.77) |
| Iran (Islamic Republic of) | 0.57 (0.34, 0.66) |
| Democratic Republic of the Congo | 0.57 (0.34, 0.88) |
| South Sudan | 0.57 (0.38, 0.83) |
| Oman | 0.56 (0.34, 0.84) |
| Benin | 0.56 (0.41, 0.75) |
| Uganda | 0.56 (0.41, 0.71) |
| Burkina Faso | 0.56 (0.41, 0.74) |
| Togo | 0.56 (0.41, 0.76) |
| Taiwan (Province of China) | 0.55 (0.38, 0.9) |
| Viet Nam | 0.55 (0.36, 0.85) |
| Honduras | 0.54 (0.37, 0.72) |
| Côte d'Ivoire | 0.54 (0.39, 0.71) |
| Georgia | 0.53 (0.34, 0.71) |
| Dominica | 0.53 (0.4, 0.69) |
| Brazil | 0.53 (0.46, 0.62) |
| Bangladesh | 0.53 (0.38, 0.72) |
| Liberia | 0.53 (0.37, 0.76) |
| Jamaica | 0.52 (0.36, 0.69) |
| Tunisia | 0.52 (0.34, 0.75) |
| Sierra Leone | 0.52 (0.38, 0.73) |
| Nicaragua | 0.52 (0.4, 0.67) |
| Panama | 0.49 (0.36, 0.68) |
| Venezuela (Bolivarian Republic of) | 0.48 (0.36, 0.65) |
| Niger | 0.47 (0.33, 0.64) |
| Chad | 0.47 (0.34, 0.63) |
| Republic of Moldova | 0.47 (0.39, 0.58) |
| Colombia | 0.46 (0.34, 0.62) |
| Ghana | 0.45 (0.34, 0.58) |
| Libya | 0.44 (0.25, 0.65) |
| Singapore | 0.43 (0.31, 0.56) |
| Palestine | 0.43 (0.3, 0.58) |
| Turkmenistan | 0.43 (0.32, 0.58) |
| Guyana | 0.41 (0.3, 0.54) |
| Bolivia (Plurinational State of) | 0.4 (0.28, 0.55) |
| Azerbaijan | 0.38 (0.28, 0.51) |
| Mexico | 0.38 (0.31, 0.48) |
| Thailand | 0.37 (0.24, 0.52) |
| Ecuador | 0.36 (0.27, 0.46) |
| Maldives | 0.35 (0.24, 0.48) |
| Belize | 0.34 (0.27, 0.41) |
| Peru | 0.34 (0.23, 0.48) |
| Suriname | 0.33 (0.25, 0.41) |
| Tajikistan | 0.33 (0.24, 0.46) |
| San Marino | 0.33 (0.23, 0.47) |
| Trinidad and Tobago | 0.31 (0.22, 0.42) |
| Haiti | 0.3 (0.18, 0.5) |
| Japan | 0.3 (0.24, 0.4) |
| Republic of Korea | 0.3 (0.2, 0.38) |
| Dominican Republic | 0.3 (0.21, 0.42) |
| Egypt | 0.29 (0.17, 0.47) |
| Iraq | 0.29 (0.2, 0.42) |
| Afghanistan | 0.29 (0.12, 0.48) |
| Democratic People's Republic of Korea | 0.28 (0.18, 0.4) |
| Sudan | 0.28 (0.12, 0.45) |
| Kyrgyzstan | 0.27 (0.22, 0.36) |
| Mongolia | 0.27 (0.19, 0.38) |
| Cambodia | 0.26 (0.19, 0.35) |
| Yemen | 0.26 (0.11, 0.42) |
| Indonesia | 0.25 (0.18, 0.34) |
| El Salvador | 0.22 (0.15, 0.3) |
| Morocco | 0.21 (0.12, 0.31) |
| Myanmar | 0.2 (0.15, 0.28) |
| Lao People's Democratic Republic | 0.19 (0.13, 0.25) |
| Timor-Leste | 0.18 (0.12, 0.27) |
| Antigua and Barbuda | 0.16 (0.12, 0.2) |
| Nauru | 0.16 (0.1, 0.22) |
| Philippines | 0.15 (0.12, 0.19) |
| Niue | 0.15 (0.11, 0.21) |
| Palau | 0.15 (0.11, 0.2) |
| Guatemala | 0.14 (0.11, 0.19) |
| Fiji | 0.13 (0.09, 0.2) |
| Mauritius | 0.12 (0.09, 0.16) |
| Samoa | 0.12 (0.08, 0.17) |
| Micronesia (Federated States of) | 0.11 (0.07, 0.16) |
| Northern Mariana Islands | 0.11 (0.08, 0.15) |
| Solomon Islands | 0.11 (0.07, 0.17) |
| Tokelau | 0.11 (0.07, 0.16) |
| Sri Lanka | 0.1 (0.07, 0.14) |
| Kiribati | 0.1 (0.06, 0.15) |
| American Samoa | 0.1 (0.07, 0.13) |
| Cook Islands | 0.1 (0.07, 0.14) |
| Marshall Islands | 0.09 (0.06, 0.12) |
| Jordan | 0.09 (0.06, 0.11) |
| Tuvalu | 0.09 (0.06, 0.13) |
| Guam | 0.08 (0.06, 0.1) |
| Tonga | 0.06 (0.04, 0.08) |
| Vanuatu | 0.06 (0.04, 0.09) |
| Papua New Guinea | 0.04 (0.02, 0.06) |

ASIR: age-standardized incidence rate, UI: uncertainty interval.

**Supplementary table S8. Age-standardized death rates of 204 countries and territories in 2019 (from high to low).**

| Countries and Territories | ASDRs per 100,000 (95% UI) |
| --- | --- |
| Qatar | 3.87 (2.44, 5.84) |
| Seychelles | 2.53 (2.12, 3.03) |
| Lesotho | 1.79 (1.17, 2.5) |
| Botswana | 1.77 (1.16, 2.49) |
| Israel | 1.74 (1.47, 2.17) |
| Eswatini | 1.65 (1.12, 2.27) |
| Poland | 1.57 (1.23, 2.14) |
| Brunei Darussalam | 1.55 (1.33, 1.81) |
| Croatia | 1.54 (1.19, 2.01) |
| Latvia | 1.5 (1.18, 1.87) |
| Montenegro | 1.48 (1.15, 1.9) |
| South Africa | 1.42 (1.09, 1.62) |
| North Macedonia | 1.41 (1.08, 1.83) |
| Hungary | 1.4 (1.14, 1.73) |
| Greenland | 1.36 (1.08, 1.68) |
| Lithuania | 1.36 (1.04, 1.81) |
| Ethiopia | 1.35 (0.84, 1.96) |
| Zimbabwe | 1.31 (0.97, 1.79) |
| Czechia | 1.3 (1.03, 1.61) |
| Slovenia | 1.24 (0.9, 1.69) |
| Cyprus | 1.23 (0.98, 1.49) |
| Andorra | 1.22 (0.87, 1.68) |
| Slovakia | 1.22 (0.92, 1.59) |
| Sweden | 1.18 (1.02, 1.35) |
| United States Virgin Islands | 1.17 (0.94, 1.45) |
| New Zealand | 1.17 (0.94, 1.34) |
| Denmark | 1.16 (0.96, 1.45) |
| Uruguay | 1.13 (0.92, 1.32) |
| Bosnia and Herzegovina | 1.13 (0.84, 1.48) |
| Austria | 1.11 (0.93, 1.39) |
| Germany | 1.1 (0.94, 1.43) |
| Mozambique | 1.09 (0.78, 1.5) |
| Ireland | 1.06 (0.85, 1.3) |
| Namibia | 1.04 (0.78, 1.39) |
| Bahrain | 1.03 (0.8, 1.35) |
| Canada | 1 (0.85, 1.2) |
| Pakistan | 0.99 (0.8, 1.24) |
| United States of America | 0.98 (0.87, 1.19) |
| Eritrea | 0.95 (0.68, 1.26) |
| Monaco | 0.94 (0.33, 1.34) |
| Portugal | 0.94 (0.78, 1.11) |
| United Kingdom | 0.93 (0.8, 1.17) |
| Italy | 0.93 (0.8, 1.07) |
| Greece | 0.93 (0.79, 1.18) |
| Russian Federation | 0.92 (0.78, 1.07) |
| France | 0.92 (0.76, 1.12) |
| Zambia | 0.89 (0.69, 1.18) |
| Belarus | 0.87 (0.66, 1.16) |
| Gabon | 0.86 (0.59, 1.2) |
| Australia | 0.86 (0.72, 1.12) |
| Malawi | 0.86 (0.65, 1.12) |
| Rwanda | 0.84 (0.65, 1.06) |
| Equatorial Guinea | 0.84 (0.5, 1.3) |
| Ukraine | 0.82 (0.68, 1) |
| Congo | 0.81 (0.58, 1.09) |
| Djibouti | 0.8 (0.59, 1.06) |
| Belgium | 0.8 (0.67, 0.95) |
| United Republic of Tanzania | 0.8 (0.63, 1) |
| Turkey | 0.78 (0.57, 1.22) |
| Comoros | 0.77 (0.59, 0.99) |
| Norway | 0.77 (0.67, 0.89) |
| Nepal | 0.75 (0.54, 1) |
| Switzerland | 0.74 (0.62, 0.9) |
| Bhutan | 0.74 (0.54, 0.95) |
| Malaysia | 0.74 (0.53, 1.13) |
| Estonia | 0.74 (0.54, 0.96) |
| Spain | 0.74 (0.61, 0.87) |
| Romania | 0.73 (0.58, 0.92) |
| Saudi Arabia | 0.73 (0.55, 1) |
| Finland | 0.71 (0.59, 0.85) |
| Cabo Verde | 0.7 (0.55, 0.9) |
| Armenia | 0.69 (0.55, 0.89) |
| Luxembourg | 0.69 (0.56, 0.83) |
| Somalia | 0.69 (0.36, 1.07) |
| Iceland | 0.68 (0.53, 0.82) |
| Saint Vincent and the Grenadines | 0.68 (0.57, 0.82) |
| Kenya | 0.68 (0.53, 0.92) |
| Nigeria | 0.68 (0.48, 0.84) |
| Angola | 0.66 (0.44, 0.93) |
| Cameroon | 0.66 (0.48, 0.91) |
| Albania | 0.64 (0.43, 0.97) |
| Burundi | 0.63 (0.44, 0.87) |
| Central African Republic | 0.63 (0.38, 0.99) |
| Bulgaria | 0.62 (0.47, 0.82) |
| Guinea | 0.61 (0.44, 0.81) |
| Madagascar | 0.61 (0.45, 0.82) |
| Guinea-Bissau | 0.61 (0.43, 0.81) |
| India | 0.61 (0.5, 0.74) |
| Gambia | 0.6 (0.44, 0.79) |
| South Sudan | 0.6 (0.4, 0.86) |
| Saint Lucia | 0.58 (0.47, 0.72) |
| Cuba | 0.57 (0.45, 0.72) |
| Mali | 0.56 (0.43, 0.73) |
| Algeria | 0.56 (0.44, 0.72) |
| Democratic Republic of the Congo | 0.56 (0.34, 0.87) |
| Bahamas | 0.55 (0.45, 0.67) |
| Uganda | 0.54 (0.4, 0.68) |
| Senegal | 0.54 (0.4, 0.71) |
| Burkina Faso | 0.52 (0.39, 0.69) |
| Malta | 0.52 (0.41, 0.67) |
| Mauritania | 0.52 (0.38, 0.68) |
| Benin | 0.52 (0.38, 0.7) |
| Syrian Arab Republic | 0.52 (0.33, 0.78) |
| Togo | 0.51 (0.38, 0.69) |
| Côte d'Ivoire | 0.51 (0.37, 0.67) |
| Liberia | 0.49 (0.34, 0.7) |
| Sierra Leone | 0.49 (0.35, 0.68) |
| Sao Tome and Principe | 0.49 (0.36, 0.65) |
| Saint Kitts and Nevis | 0.48 (0.39, 0.61) |
| Barbados | 0.47 (0.37, 0.57) |
| Kazakhstan | 0.46 (0.36, 0.59) |
| Honduras | 0.46 (0.32, 0.62) |
| Paraguay | 0.46 (0.32, 0.61) |
| Chad | 0.46 (0.34, 0.61) |
| Costa Rica | 0.45 (0.32, 0.62) |
| Bangladesh | 0.45 (0.32, 0.62) |
| Niger | 0.45 (0.32, 0.62) |
| Serbia | 0.44 (0.32, 0.57) |
| Dominica | 0.42 (0.32, 0.54) |
| Lebanon | 0.42 (0.26, 0.65) |
| United Arab Emirates | 0.42 (0.21, 0.82) |
| Grenada | 0.41 (0.34, 0.48) |
| Bermuda | 0.41 (0.32, 0.52) |
| Uzbekistan | 0.41 (0.29, 0.54) |
| Ghana | 0.4 (0.3, 0.51) |
| Argentina | 0.4 (0.33, 0.49) |
| Viet Nam | 0.39 (0.24, 0.61) |
| Chile | 0.37 (0.31, 0.45) |
| Brazil | 0.37 (0.32, 0.44) |
| Bolivia (Plurinational State of) | 0.35 (0.25, 0.48) |
| Guyana | 0.33 (0.25, 0.44) |
| Jamaica | 0.32 (0.22, 0.42) |
| Nicaragua | 0.32 (0.26, 0.41) |
| Puerto Rico | 0.31 (0.23, 0.41) |
| Venezuela (Bolivarian Republic of) | 0.31 (0.23, 0.4) |
| Panama | 0.31 (0.23, 0.43) |
| Georgia | 0.3 (0.19, 0.39) |
| Netherlands | 0.3 (0.24, 0.66) |
| Kuwait | 0.29 (0.2, 0.41) |
| Haiti | 0.29 (0.18, 0.49) |
| Colombia | 0.27 (0.19, 0.35) |
| Ecuador | 0.27 (0.2, 0.34) |
| Iran (Islamic Republic of) | 0.26 (0.17, 0.3) |
| Afghanistan | 0.26 (0.11, 0.43) |
| Republic of Moldova | 0.26 (0.22, 0.32) |
| Turkmenistan | 0.26 (0.19, 0.36) |
| Oman | 0.26 (0.16, 0.38) |
| Mexico | 0.25 (0.2, 0.31) |
| China | 0.25 (0.21, 0.31) |
| Palestine | 0.25 (0.17, 0.34) |
| Tajikistan | 0.25 (0.18, 0.36) |
| Libya | 0.25 (0.14, 0.36) |
| Suriname | 0.25 (0.2, 0.32) |
| Belize | 0.25 (0.2, 0.31) |
| Azerbaijan | 0.24 (0.18, 0.32) |
| Tunisia | 0.24 (0.16, 0.35) |
| Cambodia | 0.23 (0.16, 0.3) |
| Dominican Republic | 0.23 (0.16, 0.31) |
| Thailand | 0.23 (0.14, 0.32) |
| Sudan | 0.22 (0.1, 0.34) |
| Indonesia | 0.22 (0.16, 0.29) |
| Yemen | 0.21 (0.09, 0.33) |
| Trinidad and Tobago | 0.2 (0.15, 0.27) |
| Peru | 0.2 (0.14, 0.27) |
| Taiwan (Province of China) | 0.19 (0.14, 0.31) |
| Maldives | 0.19 (0.13, 0.26) |
| Egypt | 0.18 (0.1, 0.29) |
| Myanmar | 0.18 (0.13, 0.24) |
| Iraq | 0.18 (0.12, 0.26) |
| Mongolia | 0.18 (0.13, 0.26) |
| Timor-Leste | 0.17 (0.11, 0.25) |
| Lao People's Democratic Republic | 0.17 (0.12, 0.23) |
| Democratic People's Republic of Korea | 0.16 (0.11, 0.23) |
| Kyrgyzstan | 0.16 (0.13, 0.22) |
| Morocco | 0.15 (0.09, 0.22) |
| El Salvador | 0.14 (0.1, 0.18) |
| Singapore | 0.14 (0.11, 0.17) |
| Philippines | 0.12 (0.1, 0.15) |
| Guatemala | 0.11 (0.09, 0.15) |
| Fiji | 0.11 (0.08, 0.16) |
| Kiribati | 0.1 (0.06, 0.14) |
| Nauru | 0.1 (0.07, 0.14) |
| Antigua and Barbuda | 0.1 (0.07, 0.12) |
| Japan | 0.08 (0.07, 0.11) |
| Niue | 0.08 (0.05, 0.11) |
| Micronesia (Federated States of) | 0.08 (0.05, 0.12) |
| Solomon Islands | 0.08 (0.05, 0.13) |
| Samoa | 0.08 (0.05, 0.12) |
| Tokelau | 0.07 (0.04, 0.1) |
| Republic of Korea | 0.07 (0.05, 0.08) |
| San Marino | 0.07 (0.04, 0.11) |
| Tuvalu | 0.07 (0.05, 0.1) |
| Palau | 0.07 (0.05, 0.1) |
| Marshall Islands | 0.07 (0.05, 0.1) |
| American Samoa | 0.06 (0.05, 0.09) |
| Mauritius | 0.06 (0.05, 0.08) |
| Vanuatu | 0.05 (0.04, 0.08) |
| Sri Lanka | 0.05 (0.04, 0.07) |
| Northern Mariana Islands | 0.05 (0.03, 0.06) |
| Jordan | 0.04 (0.03, 0.06) |
| Cook Islands | 0.04 (0.03, 0.06) |
| Guam | 0.04 (0.03, 0.05) |
| Tonga | 0.04 (0.03, 0.06) |
| Papua New Guinea | 0.03 (0.02, 0.05) |

ASDR: age-standardized death rate, UI: uncertainty interval.

**Supplementary table S9. Age-standardized DALY rates of 204 countries and territories in 2019 (from high to low).**

| Countries and Territories | Age-Standardized DALY Rates per 100,000  (95% UI) |
| --- | --- |
| Qatar | 54.89 (38.8, 78.2) |
| Seychelles | 54.31 (45.79, 64.76) |
| Lesotho | 38.06 (24.43, 53.76) |
| Botswana | 37.49 (24.32, 53.85) |
| Eswatini | 33.63 (21.96, 48.42) |
| Poland | 33.3 (25.82, 47.1) |
| Brunei Darussalam | 33.18 (27.99, 39.41) |
| Montenegro | 32.13 (24.96, 41.87) |
| Latvia | 31.48 (24.57, 39.87) |
| Zimbabwe | 31.28 (22.61, 42.08) |
| North Macedonia | 30.92 (22.58, 40.86) |
| Israel | 30.64 (25.89, 39.43) |
| Croatia | 30.01 (22.54, 40.35) |
| Hungary | 29.04 (23.29, 36.97) |
| South Africa | 29.02 (23.75, 33.75) |
| Lithuania | 28.51 (21.48, 38.52) |
| Greenland | 28.2 (22.27, 35.34) |
| Czechia | 25.86 (20.45, 32.64) |
| United States Virgin Islands | 25.44 (20.15, 32.57) |
| Slovakia | 25.41 (19.14, 34.06) |
| Ethiopia | 25.04 (15.88, 35.6) |
| Slovenia | 24.2 (17.48, 32.66) |
| Bosnia and Herzegovina | 24.17 (17.36, 32.71) |
| Andorra | 23.39 (16.56, 32.44) |
| Pakistan | 22.99 (18.43, 29.18) |
| Namibia | 22.31 (16.22, 31.29) |
| Cyprus | 22.18 (18, 27.16) |
| Belarus | 21.75 (16.06, 28.62) |
| Russian Federation | 21.7 (18.15, 25.33) |
| New Zealand | 21.65 (17.47, 24.84) |
| Denmark | 21.5 (17.74, 27.15) |
| Ukraine | 21.46 (17.73, 26.46) |
| Germany | 20.97 (17.91, 27.93) |
| Sweden | 20.32 (17.83, 23.67) |
| Uruguay | 20.08 (16.66, 23.27) |
| Austria | 20.03 (16.77, 26.42) |
| Ireland | 19.89 (15.81, 24.25) |
| Mozambique | 19.33 (13.63, 27.58) |
| Canada | 19.22 (16.08, 23.15) |
| Eritrea | 18.69 (13.07, 25.67) |
| Monaco | 18.54 (6.2, 27.32) |
| Malawi | 18.28 (13.34, 24.35) |
| United States of America | 18.27 (16.47, 22.54) |
| Zambia | 17.91 (13.34, 24.53) |
| Armenia | 17.56 (13.85, 23.67) |
| Greece | 17.55 (14.94, 23.14) |
| Portugal | 17.55 (14.71, 21.39) |
| Italy | 17.5 (15.37, 20.36) |
| Congo | 16.98 (11.91, 24.19) |
| Gabon | 16.97 (11.36, 24.29) |
| Bahrain | 16.85 (13.06, 22.01) |
| Romania | 16.84 (13.39, 21.46) |
| Equatorial Guinea | 16.54 (9.42, 26.92) |
| France | 16.34 (13.58, 20.34) |
| United Kingdom | 16.18 (14.38, 20.42) |
| Turkey | 15.94 (11.71, 24.81) |
| Estonia | 15.9 (11.23, 20.88) |
| Rwanda | 15.89 (12.15, 20.53) |
| Australia | 15.85 (13.4, 20.8) |
| United Republic of Tanzania | 15.65 (12.32, 19.98) |
| Djibouti | 15.46 (11.14, 21.47) |
| Comoros | 15.21 (11.31, 19.91) |
| Saudi Arabia | 14.9 (11.05, 20.48) |
| Norway | 14.87 (13.07, 17.27) |
| Albania | 14.83 (8.79, 22.74) |
| Saint Vincent and the Grenadines | 14.77 (12.24, 17.77) |
| Nepal | 14.58 (10.5, 19.98) |
| Belgium | 14.5 (12.26, 17.44) |
| Nigeria | 14.4 (10.17, 18.18) |
| Cameroon | 14.16 (10.23, 19.67) |
| Bhutan | 14.06 (10.11, 18.61) |
| Cabo Verde | 14.03 (11.14, 18.18) |
| Somalia | 13.9 (7.25, 22.01) |
| Bulgaria | 13.89 (10.21, 18.55) |
| Guinea | 13.79 (9.84, 18.34) |
| Angola | 13.68 (9.13, 19.56) |
| Central African Republic | 13.67 (8.35, 20.67) |
| Spain | 13.61 (11.49, 16.06) |
| Gambia | 13.54 (9.87, 17.89) |
| Finland | 13.53 (10.97, 16.57) |
| Malaysia | 13.37 (9.67, 20.4) |
| Iceland | 13.31 (10.35, 16.67) |
| Kenya | 13.29 (10.27, 18.25) |
| Guinea-Bissau | 13.29 (9.32, 17.8) |
| Switzerland | 13.27 (11.15, 16.16) |
| Luxembourg | 13.1 (10.66, 16.22) |
| Saint Lucia | 12.69 (10.11, 15.8) |
| Mali | 12.63 (9.54, 16.89) |
| Burundi | 12.61 (8.64, 17.7) |
| Madagascar | 12.41 (8.88, 17.03) |
| India | 12.39 (10.13, 15.04) |
| Bahamas | 12.15 (9.87, 15) |
| Syrian Arab Republic | 11.97 (7.7, 18.03) |
| Cuba | 11.92 (9.24, 15.51) |
| Democratic Republic of the Congo | 11.81 (7.01, 18.1) |
| Kazakhstan | 11.77 (9.17, 15.14) |
| South Sudan | 11.6 (7.48, 17.31) |
| Senegal | 11.44 (8.41, 15.3) |
| Saint Kitts and Nevis | 11.23 (8.81, 14.53) |
| Burkina Faso | 11.2 (8.27, 15.09) |
| Benin | 11.12 (8.1, 15.1) |
| Mauritania | 11.05 (7.82, 14.98) |
| Serbia | 11.01 (7.88, 14.52) |
| Togo | 10.94 (7.91, 14.99) |
| Algeria | 10.89 (7.96, 14.07) |
| Sao Tome and Principe | 10.85 (7.92, 14.4) |
| Côte d'Ivoire | 10.66 (7.8, 14.24) |
| Uganda | 10.65 (7.87, 13.67) |
| Liberia | 10.58 (7.22, 15.21) |
| Sierra Leone | 10.58 (7.61, 14.84) |
| Uzbekistan | 10.22 (7.24, 13.28) |
| Chad | 9.98 (7.23, 13.53) |
| Lebanon | 9.97 (6.17, 15.06) |
| Niger | 9.84 (6.93, 13.36) |
| Barbados | 9.59 (7.57, 11.75) |
| Malta | 9.4 (7.37, 12.22) |
| United Arab Emirates | 9.37 (4.86, 17.51) |
| Grenada | 9.29 (7.72, 11.18) |
| Paraguay | 9.2 (6.51, 12.55) |
| Honduras | 9.15 (6.13, 12.18) |
| Bangladesh | 9.03 (6.52, 12.39) |
| Bermuda | 8.8 (6.89, 11.5) |
| Dominica | 8.74 (6.51, 11.52) |
| Ghana | 8.34 (6.34, 10.71) |
| Costa Rica | 8.02 (5.77, 10.97) |
| China | 7.85 (6.51, 9.5) |
| Guyana | 7.71 (5.65, 10.13) |
| Argentina | 7.5 (6.38, 9.3) |
| Georgia | 7.47 (4.55, 9.98) |
| Afghanistan | 7.32 (3.08, 12.21) |
| Nicaragua | 7.29 (5.66, 9.4) |
| Jamaica | 7.19 (5.01, 9.5) |
| Bolivia (Plurinational State of) | 7.12 (4.96, 9.62) |
| Brazil | 7.05 (6.29, 8.28) |
| Turkmenistan | 6.79 (5, 9.17) |
| Iran (Islamic Republic of) | 6.74 (4.05, 7.74) |
| Viet Nam | 6.73 (4.43, 10.28) |
| Chile | 6.62 (5.62, 8.13) |
| Kuwait | 6.48 (4.57, 9.18) |
| Haiti | 6.39 (3.96, 10.65) |
| Venezuela (Bolivarian Republic of) | 6.38 (4.68, 8.54) |
| Libya | 6.15 (3.51, 8.96) |
| Tajikistan | 6.08 (4.35, 8.69) |
| Puerto Rico | 5.99 (4.35, 7.97) |
| Panama | 5.98 (4.35, 8.48) |
| Netherlands | 5.97 (4.6, 13.24) |
| Republic of Moldova | 5.96 (4.9, 7.26) |
| Palestine | 5.74 (4.05, 7.7) |
| Belize | 5.74 (4.65, 7.01) |
| Azerbaijan | 5.68 (4.26, 7.77) |
| Suriname | 5.65 (4.41, 7.16) |
| Tunisia | 5.57 (3.7, 8.03) |
| Oman | 5.5 (3.39, 8.03) |
| Democratic People's Republic of Korea | 5.49 (3.4, 8.38) |
| Colombia | 5.49 (3.98, 7.36) |
| Sudan | 5.48 (2.4, 8.69) |
| Yemen | 5.42 (2.27, 8.68) |
| Ecuador | 5.19 (3.85, 6.81) |
| Mexico | 5.03 (4.1, 6.38) |
| Cambodia | 4.96 (3.51, 6.56) |
| Peru | 4.92 (3.3, 6.92) |
| Trinidad and Tobago | 4.91 (3.46, 6.68) |
| Dominican Republic | 4.83 (3.34, 6.86) |
| Egypt | 4.76 (2.85, 8) |
| Mongolia | 4.71 (3.32, 6.72) |
| Indonesia | 4.54 (3.37, 6.28) |
| Thailand | 4.31 (2.72, 6.06) |
| Kyrgyzstan | 4.29 (3.46, 5.79) |
| Iraq | 4.25 (2.87, 6.18) |
| Taiwan (Province of China) | 3.93 (2.77, 6.37) |
| Myanmar | 3.93 (2.76, 5.49) |
| Maldives | 3.83 (2.56, 5.33) |
| Lao People's Democratic Republic | 3.81 (2.73, 5.22) |
| Timor-Leste | 3.55 (2.34, 5.37) |
| Morocco | 3.52 (2.05, 5.14) |
| El Salvador | 3.14 (2.17, 4.23) |
| Nauru | 2.88 (1.8, 3.96) |
| Singapore | 2.79 (2.06, 3.36) |
| Philippines | 2.78 (2.26, 3.58) |
| Fiji | 2.71 (1.85, 4.09) |
| Guatemala | 2.67 (2.01, 3.46) |
| Solomon Islands | 2.5 (1.48, 3.92) |
| Kiribati | 2.36 (1.37, 3.4) |
| Micronesia (Federated States of) | 2.31 (1.46, 3.41) |
| Samoa | 2.2 (1.48, 3.2) |
| Antigua and Barbuda | 2.13 (1.69, 2.7) |
| Palau | 2.08 (1.53, 2.81) |
| Niue | 2.06 (1.44, 2.9) |
| Marshall Islands | 2.04 (1.39, 2.9) |
| Tuvalu | 1.96 (1.37, 2.76) |
| Tokelau | 1.94 (1.29, 2.92) |
| Republic of Korea | 1.82 (1.26, 2.23) |
| San Marino | 1.78 (1.1, 2.73) |
| American Samoa | 1.76 (1.29, 2.36) |
| Japan | 1.76 (1.53, 2.29) |
| Mauritius | 1.64 (1.26, 2.07) |
| Vanuatu | 1.58 (1.02, 2.3) |
| Northern Mariana Islands | 1.39 (1.03, 1.86) |
| Guam | 1.28 (0.99, 1.6) |
| Sri Lanka | 1.2 (0.85, 1.66) |
| Cook Islands | 1.16 (0.82, 1.63) |
| Tonga | 1.11 (0.76, 1.63) |
| Jordan | 1.03 (0.76, 1.33) |
| Papua New Guinea | 0.99 (0.61, 1.51) |

DALY: disability-adjusted life year, UI: uncertainty interval.

**Supplementary table S10. The EAPCs of ASIRs of 204 countries and territories from1990 to 2019 (from high to low).**

| countries and territories | EAPCs of ASIRs  (95% CI) |
| --- | --- |
| Poland | 7.48 (6.25, 8.74) |
| Jamaica | 7.48 (6.46, 8.51) |
| Estonia | 7.09 (5.13, 9.09) |
| China | 6 (5.55, 6.46) |
| Bosnia and Herzegovina | 5.61 (4.98, 6.24) |
| Equatorial Guinea | 5.43 (4.88, 5.99) |
| Albania | 5.3 (4.88, 5.73) |
| El Salvador | 5.09 (4.71, 5.47) |
| North Macedonia | 4.99 (4.35, 5.64) |
| Republic of Korea | 4.48 (3.83, 5.14) |
| Dominican Republic | 4.46 (4.16, 4.76) |
| Serbia | 4.46 (4.04, 4.87) |
| Georgia | 4.2 (2.72, 5.69) |
| Montenegro | 4.15 (3.88, 4.43) |
| Ecuador | 4.07 (3.66, 4.48) |
| Paraguay | 3.95 (3.67, 4.23) |
| Lesotho | 3.79 (3.37, 4.21) |
| Cyprus | 3.75 (3.3, 4.21) |
| Honduras | 3.63 (3.37, 3.89) |
| Angola | 3.62 (3.29, 3.96) |
| Lebanon | 3.57 (3.26, 3.89) |
| Romania | 3.5 (3.21, 3.79) |
| Guatemala | 3.14 (2.73, 3.56) |
| Oman | 3.09 (2.5, 3.69) |
| Guyana | 3.07 (2.21, 3.93) |
| Qatar | 3.06 (2.67, 3.44) |
| Saudi Arabia | 2.9 (2.51, 3.29) |
| Kuwait | 2.89 (2.46, 3.32) |
| Cabo Verde | 2.86 (2.53, 3.19) |
| Democratic Republic of the Congo | 2.85 (2.56, 3.15) |
| Peru | 2.82 (2.58, 3.06) |
| Bulgaria | 2.81 (2.52, 3.09) |
| Portugal | 2.79 (2.36, 3.22) |
| Egypt | 2.7 (2.51, 2.89) |
| Gabon | 2.69 (2.25, 3.14) |
| Central African Republic | 2.66 (2.21, 3.12) |
| Indonesia | 2.64 (2.57, 2.71) |
| Russian Federation | 2.62 (2.33, 2.92) |
| Eritrea | 2.61 (2.34, 2.87) |
| Congo | 2.6 (2.31, 2.89) |
| Viet Nam | 2.59 (2.42, 2.76) |
| Czechia | 2.57 (1.72, 3.42) |
| Bolivia (Plurinational State of) | 2.57 (2.48, 2.66) |
| Suriname | 2.53 (2.21, 2.86) |
| Timor-Leste | 2.48 (2.21, 2.76) |
| Palestine | 2.47 (1.94, 3) |
| Thailand | 2.45 (2.28, 2.61) |
| Mozambique | 2.42 (2.26, 2.58) |
| Belize | 2.42 (2.1, 2.75) |
| Turkey | 2.36 (2.12, 2.6) |
| Libya | 2.35 (2.07, 2.63) |
| Lithuania | 2.29 (1.41, 3.18) |
| Tunisia | 2.28 (2.16, 2.4) |
| Djibouti | 2.24 (2.09, 2.39) |
| Sudan | 2.18 (1.94, 2.42) |
| Yemen | 2.17 (2.03, 2.31) |
| Malaysia | 2.16 (1.76, 2.56) |
| Botswana | 2.11 (1.87, 2.35) |
| United Republic of Tanzania | 2.11 (1.96, 2.27) |
| Somalia | 2.04 (1.86, 2.23) |
| Jordan | 2.04 (1.83, 2.25) |
| Zambia | 2 (1.93, 2.07) |
| Comoros | 2 (1.92, 2.07) |
| Iraq | 1.97 (1.58, 2.37) |
| Azerbaijan | 1.92 (1.68, 2.15) |
| San Marino | 1.92 (1.73, 2.11) |
| Madagascar | 1.89 (1.69, 2.09) |
| Namibia | 1.85 (1.69, 2) |
| Nepal | 1.83 (1.6, 2.06) |
| Bhutan | 1.79 (1.67, 1.91) |
| Uganda | 1.79 (1.57, 2.01) |
| Brunei Darussalam | 1.78 (1.67, 1.9) |
| Barbados | 1.77 (1.67, 1.87) |
| Mongolia | 1.77 (1.41, 2.14) |
| Kazakhstan | 1.75 (1.51, 1.98) |
| Antigua and Barbuda | 1.74 (1.47, 2.01) |
| Belarus | 1.72 (1.59, 1.84) |
| Singapore | 1.71 (1.34, 2.08) |
| Mexico | 1.7 (1.52, 1.89) |
| Chile | 1.7 (1.43, 1.97) |
| Monaco | 1.68 (1.28, 2.09) |
| Morocco | 1.66 (1.33, 1.98) |
| Slovakia | 1.66 (1.53, 1.78) |
| Saint Vincent and the Grenadines | 1.62 (1.39, 1.86) |
| Cambodia | 1.61 (1.46, 1.76) |
| Maldives | 1.57 (1.32, 1.83) |
| Eswatini | 1.56 (1.04, 2.07) |
| United States Virgin Islands | 1.52 (1.23, 1.81) |
| Spain | 1.51 (1.11, 1.91) |
| Turkmenistan | 1.49 (1.28, 1.71) |
| Sao Tome and Principe | 1.42 (1.28, 1.55) |
| Ireland | 1.38 (1.06, 1.7) |
| Nigeria | 1.35 (1.22, 1.48) |
| Sri Lanka | 1.34 (1.08, 1.59) |
| Brazil | 1.3 (1.19, 1.41) |
| Sierra Leone | 1.28 (1.14, 1.41) |
| Rwanda | 1.27 (1.12, 1.42) |
| Uzbekistan | 1.26 (1.11, 1.4) |
| Germany | 1.26 (0.89, 1.62) |
| Syrian Arab Republic | 1.26 (0.92, 1.6) |
| South Sudan | 1.22 (1.08, 1.36) |
| Taiwan (Province of China) | 1.2 (0.44, 1.97) |
| Dominica | 1.19 (1.04, 1.35) |
| Costa Rica | 1.18 (0.9, 1.47) |
| Pakistan | 1.18 (1, 1.37) |
| Croatia | 1.16 (0.65, 1.67) |
| Algeria | 1.14 (1.07, 1.21) |
| Gambia | 1.14 (0.96, 1.32) |
| Kenya | 1.13 (1.04, 1.23) |
| Bermuda | 1.12 (1, 1.24) |
| Senegal | 1.1 (0.91, 1.3) |
| Iran (Islamic Republic of) | 1.1 (0.68, 1.52) |
| United Arab Emirates | 1.06 (0.38, 1.73) |
| Hungary | 1.05 (0.64, 1.46) |
| Latvia | 1.04 (0.76, 1.32) |
| Tajikistan | 1.02 (0.7, 1.34) |
| Malawi | 1.02 (0.87, 1.18) |
| Chad | 1.01 (0.9, 1.12) |
| Saint Kitts and Nevis | 1.01 (0.81, 1.2) |
| Uruguay | 1 (0.76, 1.24) |
| Myanmar | 1 (0.94, 1.05) |
| Andorra | 0.99 (0.73, 1.26) |
| Greece | 0.97 (0.56, 1.39) |
| Burkina Faso | 0.96 (0.85, 1.08) |
| New Zealand | 0.95 (0.66, 1.24) |
| Mauritius | 0.95 (0.52, 1.38) |
| Democratic People's Republic of Korea | 0.94 (0.67, 1.22) |
| Liberia | 0.92 (0.68, 1.16) |
| Bahamas | 0.88 (0.69, 1.08) |
| Zimbabwe | 0.87 (0.61, 1.14) |
| Niger | 0.87 (0.8, 0.93) |
| Palau | 0.86 (0.78, 0.94) |
| Sweden | 0.85 (0.44, 1.26) |
| Seychelles | 0.84 (0.74, 0.95) |
| Trinidad and Tobago | 0.84 (0.47, 1.22) |
| Afghanistan | 0.83 (0.72, 0.95) |
| Benin | 0.82 (0.72, 0.92) |
| Cuba | 0.82 (0.73, 0.92) |
| Niue | 0.78 (0.66, 0.9) |
| Cameroon | 0.78 (0.72, 0.85) |
| India | 0.77 (0.56, 0.99) |
| Japan | 0.74 (0.54, 0.94) |
| Burundi | 0.73 (0.61, 0.84) |
| Panama | 0.73 (0.27, 1.19) |
| Togo | 0.71 (0.61, 0.81) |
| Italy | 0.71 (0.32, 1.11) |
| Austria | 0.68 (0.08, 1.29) |
| South Africa | 0.68 (0.42, 0.94) |
| Norway | 0.66 (0.2, 1.13) |
| Denmark | 0.65 (0.39, 0.92) |
| Belgium | 0.62 (0.29, 0.95) |
| Guinea-Bissau | 0.61 (0.55, 0.68) |
| Guinea | 0.6 (0.53, 0.66) |
| Haiti | 0.59 (0.48, 0.71) |
| Nicaragua | 0.58 (0.15, 1.01) |
| Ethiopia | 0.57 (0.38, 0.75) |
| France | 0.56 (0.22, 0.9) |
| Armenia | 0.55 (0.34, 0.77) |
| Israel | 0.55 (0.19, 0.9) |
| Tokelau | 0.54 (0.42, 0.66) |
| Slovenia | 0.53 (0.37, 0.7) |
| Côte d'Ivoire | 0.52 (0.45, 0.59) |
| Canada | 0.47 (0.2, 0.73) |
| Lao People's Democratic Republic | 0.45 (0.31, 0.6) |
| Venezuela (Bolivarian Republic of) | 0.43 (0.1, 0.76) |
| Colombia | 0.42 (0.25, 0.59) |
| Bangladesh | 0.4 (0.25, 0.55) |
| Luxembourg | 0.39 (-0.18, 0.96) |
| Saint Lucia | 0.37 (0.15, 0.59) |
| Solomon Islands | 0.36 (0.15, 0.56) |
| Micronesia (Federated States of) | 0.35 (0.24, 0.46) |
| Ukraine | 0.33 (0.15, 0.51) |
| Mali | 0.32 (0.27, 0.37) |
| Mauritania | 0.29 (0.17, 0.41) |
| Finland | 0.27 (-0.17, 0.72) |
| Ghana | 0.27 (0.2, 0.35) |
| Malta | 0.21 (-0.09, 0.52) |
| United Kingdom | 0.19 (-0.1, 0.48) |
| Republic of Moldova | 0.07 (-0.34, 0.49) |
| Australia | 0.05 (-0.22, 0.32) |
| Nauru | 0.02 (-0.28, 0.32) |
| Papua New Guinea | 0.01 (-0.09, 0.1) |
| Kiribati | -0.01 (-0.1, 0.08) |
| Tonga | -0.03 (-0.3, 0.23) |
| Fiji | -0.04 (-0.17, 0.09) |
| Samoa | -0.1 (-0.22, 0.02) |
| Marshall Islands | -0.19 (-0.32, -0.06) |
| Vanuatu | -0.2 (-0.45, 0.06) |
| Cook Islands | -0.22 (-0.43, -0.01) |
| Tuvalu | -0.28 (-0.42, -0.15) |
| Argentina | -0.29 (-0.71, 0.13) |
| Puerto Rico | -0.32 (-0.52, -0.11) |
| Bahrain | -0.33 (-0.7, 0.05) |
| Grenada | -0.4 (-1.1, 0.31) |
| American Samoa | -0.42 (-0.88, 0.05) |
| United States of America | -0.8 (-1.08, -0.52) |
| Philippines | -0.99 (-1.36, -0.62) |
| Greenland | -1.16 (-1.47, -0.85) |
| Iceland | -1.23 (-1.81, -0.65) |
| Switzerland | -1.48 (-1.72, -1.25) |
| Northern Mariana Islands | -1.59 (-2.12, -1.05) |
| Kyrgyzstan | -2.42 (-2.69, -2.14) |
| Guam | -3.17 (-4.01, -2.32) |
| Netherlands | -3.35 (-4.69, -2) |

ASIR: age-standardized incidence rate, EAPCs: estimated annual percentage changes, CI: confidence interval.

**Supplementary table S11. The EAPCs of ASDRs of 204 countries and territories from1990 to 2019 (from high to low).**

| countries and territories | EAPCs of ASDRs  (95% CI) |
| --- | --- |
| Jamaica | 6.68 (5.76, 7.6) |
| Poland | 5.28 (4.15, 6.43) |
| Estonia | 4.82 (2.79, 6.89) |
| Equatorial Guinea | 4.77 (4.23, 5.32) |
| Georgia | 4.39 (2.86, 5.93) |
| Dominican Republic | 3.82 (3.5, 4.14) |
| El Salvador | 3.77 (3.33, 4.21) |
| Bosnia and Herzegovina | 3.76 (3.27, 4.26) |
| Lesotho | 3.63 (3.2, 4.07) |
| Angola | 3.38 (3.03, 3.74) |
| North Macedonia | 3.22 (2.63, 3.81) |
| Paraguay | 3.2 (2.88, 3.52) |
| Honduras | 3.19 (2.9, 3.47) |
| Ecuador | 3.16 (2.7, 3.63) |
| Albania | 3.1 (2.68, 3.52) |
| Montenegro | 2.79 (2.47, 3.11) |
| Democratic Republic of the Congo | 2.74 (2.44, 3.04) |
| Central African Republic | 2.64 (2.2, 3.09) |
| Guyana | 2.6 (1.8, 3.42) |
| Serbia | 2.6 (2.21, 3) |
| Eritrea | 2.39 (2.11, 2.66) |
| Gabon | 2.38 (1.92, 2.84) |
| China | 2.32 (2.08, 2.56) |
| Congo | 2.32 (2.03, 2.62) |
| Indonesia | 2.27 (2.17, 2.36) |
| Guatemala | 2.25 (1.89, 2.61) |
| Mozambique | 2.19 (2.04, 2.34) |
| Timor-Leste | 2.1 (1.87, 2.33) |
| Bolivia (Plurinational State of) | 2.06 (2, 2.11) |
| Somalia | 2.03 (1.85, 2.22) |
| Suriname | 2 (1.66, 2.33) |
| Cabo Verde | 1.97 (1.6, 2.34) |
| Djibouti | 1.91 (1.77, 2.05) |
| United Republic of Tanzania | 1.85 (1.71, 1.98) |
| Belize | 1.83 (1.5, 2.16) |
| Romania | 1.81 (1.55, 2.06) |
| Madagascar | 1.73 (1.55, 1.91) |
| Bulgaria | 1.7 (1.48, 1.92) |
| Comoros | 1.66 (1.59, 1.74) |
| Zambia | 1.65 (1.57, 1.72) |
| Egypt | 1.65 (1.48, 1.82) |
| Palestine | 1.57 (1.15, 2) |
| Yemen | 1.57 (1.46, 1.67) |
| Sudan | 1.56 (1.35, 1.78) |
| Uganda | 1.53 (1.32, 1.75) |
| Oman | 1.46 (1.12, 1.81) |
| Eswatini | 1.41 (0.84, 1.98) |
| Lithuania | 1.39 (0.56, 2.22) |
| Nepal | 1.38 (1.17, 1.59) |
| Viet Nam | 1.37 (1.27, 1.47) |
| Kenya | 1.37 (1.26, 1.48) |
| Botswana | 1.36 (1.05, 1.67) |
| Azerbaijan | 1.25 (1.08, 1.42) |
| Namibia | 1.25 (1.07, 1.44) |
| Peru | 1.25 (1.01, 1.5) |
| Saint Vincent and the Grenadines | 1.24 (1.04, 1.44) |
| Qatar | 1.24 (0.91, 1.58) |
| Zimbabwe | 1.21 (0.87, 1.55) |
| Kuwait | 1.13 (0.67, 1.59) |
| Tajikistan | 1.09 (0.84, 1.34) |
| South Sudan | 1.09 (0.96, 1.21) |
| Cambodia | 1.06 (0.95, 1.16) |
| Czechia | 1.05 (0.42, 1.68) |
| Sierra Leone | 1.04 (0.91, 1.18) |
| Antigua and Barbuda | 1.04 (0.75, 1.33) |
| Dominica | 1.02 (0.87, 1.18) |
| Thailand | 1 (0.83, 1.17) |
| Malaysia | 0.99 (0.53, 1.46) |
| Libya | 0.98 (0.82, 1.14) |
| Rwanda | 0.95 (0.82, 1.08) |
| Bhutan | 0.95 (0.88, 1.01) |
| Russian Federation | 0.95 (0.69, 1.22) |
| Chad | 0.93 (0.8, 1.05) |
| Gambia | 0.93 (0.76, 1.1) |
| Nigeria | 0.93 (0.85, 1.01) |
| Sao Tome and Principe | 0.93 (0.79, 1.07) |
| Cyprus | 0.89 (0.58, 1.2) |
| Barbados | 0.89 (0.82, 0.96) |
| Senegal | 0.87 (0.7, 1.05) |
| Uzbekistan | 0.87 (0.73, 1.01) |
| Malawi | 0.84 (0.69, 0.99) |
| Morocco | 0.83 (0.48, 1.18) |
| Pakistan | 0.8 (0.58, 1.02) |
| Mongolia | 0.78 (0.36, 1.2) |
| Iraq | 0.75 (0.44, 1.06) |
| Mexico | 0.73 (0.62, 0.84) |
| Burkina Faso | 0.73 (0.61, 0.85) |
| Niger | 0.66 (0.6, 0.72) |
| Brunei Darussalam | 0.65 (0.51, 0.79) |
| Benin | 0.64 (0.54, 0.74) |
| United States Virgin Islands | 0.63 (0.35, 0.91) |
| Liberia | 0.62 (0.42, 0.83) |
| Portugal | 0.61 (0.32, 0.91) |
| Monaco | 0.6 (0.28, 0.92) |
| Democratic People's Republic of Korea | 0.59 (0.47, 0.7) |
| Afghanistan | 0.57 (0.47, 0.66) |
| Turkmenistan | 0.57 (0.39, 0.75) |
| Burundi | 0.56 (0.45, 0.67) |
| Republic of Korea | 0.55 (0.12, 0.99) |
| Lebanon | 0.55 (0.35, 0.76) |
| Myanmar | 0.54 (0.48, 0.6) |
| Togo | 0.53 (0.44, 0.62) |
| Cameroon | 0.51 (0.43, 0.59) |
| Kazakhstan | 0.47 (0.24, 0.7) |
| Brazil | 0.47 (0.4, 0.55) |
| Guinea | 0.46 (0.39, 0.53) |
| Tunisia | 0.45 (0.32, 0.57) |
| San Marino | 0.45 (0.19, 0.72) |
| Guinea-Bissau | 0.45 (0.39, 0.51) |
| Haiti | 0.42 (0.31, 0.52) |
| Côte d'Ivoire | 0.36 (0.28, 0.44) |
| Turkey | 0.33 (0.16, 0.51) |
| Saudi Arabia | 0.32 (-0.06, 0.71) |
| Jordan | 0.3 (0.03, 0.58) |
| Ethiopia | 0.29 (0.13, 0.44) |
| South Africa | 0.29 (-0.04, 0.62) |
| United Arab Emirates | 0.23 (-0.42, 0.88) |
| Bahamas | 0.23 (0.1, 0.36) |
| Uruguay | 0.17 (-0.04, 0.38) |
| Costa Rica | 0.15 (-0.12, 0.42) |
| Sweden | 0.13 (-0.1, 0.36) |
| Lao People's Democratic Republic | 0.12 (0, 0.24) |
| India | 0.12 (-0.07, 0.31) |
| Chile | 0.11 (-0.12, 0.34) |
| Mali | 0.1 (0.06, 0.14) |
| Germany | 0.07 (-0.03, 0.17) |
| Palau | 0.06 (0.02, 0.1) |
| Saint Kitts and Nevis | 0.05 (-0.12, 0.21) |
| Slovakia | 0.03 (-0.09, 0.14) |
| Ghana | 0.01 (-0.06, 0.08) |
| Algeria | -0.06 (-0.1, -0.02) |
| Trinidad and Tobago | -0.07 (-0.46, 0.32) |
| Croatia | -0.08 (-0.62, 0.46) |
| Spain | -0.08 (-0.3, 0.13) |
| Seychelles | -0.09 (-0.17, -0.02) |
| Solomon Islands | -0.09 (-0.25, 0.07) |
| Mauritius | -0.09 (-0.58, 0.4) |
| Panama | -0.12 (-0.54, 0.31) |
| Mauritania | -0.13 (-0.23, -0.03) |
| Kiribati | -0.13 (-0.23, -0.04) |
| Belarus | -0.14 (-0.31, 0.03) |
| New Zealand | -0.14 (-0.33, 0.06) |
| Papua New Guinea | -0.2 (-0.29, -0.11) |
| Sri Lanka | -0.24 (-0.51, 0.03) |
| Syrian Arab Republic | -0.26 (-0.58, 0.06) |
| Greece | -0.27 (-0.6, 0.06) |
| Bangladesh | -0.29 (-0.48, -0.1) |
| Micronesia (Federated States of) | -0.29 (-0.4, -0.19) |
| Fiji | -0.3 (-0.45, -0.15) |
| Austria | -0.36 (-0.84, 0.12) |
| Cuba | -0.39 (-0.53, -0.25) |
| Singapore | -0.41 (-0.74, -0.08) |
| Iran (Islamic Republic of) | -0.41 (-0.82, 0) |
| Vanuatu | -0.46 (-0.68, -0.23) |
| Maldives | -0.48 (-0.65, -0.32) |
| Andorra | -0.49 (-0.59, -0.4) |
| Nauru | -0.51 (-0.66, -0.37) |
| Niue | -0.52 (-0.62, -0.42) |
| Latvia | -0.52 (-0.75, -0.28) |
| Saint Lucia | -0.52 (-0.78, -0.25) |
| Tokelau | -0.53 (-0.62, -0.44) |
| Tonga | -0.55 (-0.83, -0.27) |
| Nicaragua | -0.56 (-1.09, -0.02) |
| Hungary | -0.57 (-0.85, -0.29) |
| Marshall Islands | -0.58 (-0.68, -0.49) |
| Italy | -0.58 (-0.76, -0.39) |
| Taiwan (Province of China) | -0.65 (-1.29, -0.01) |
| Armenia | -0.69 (-0.87, -0.51) |
| Samoa | -0.71 (-0.83, -0.6) |
| Denmark | -0.71 (-0.89, -0.53) |
| United Kingdom | -0.72 (-0.92, -0.51) |
| Venezuela (Bolivarian Republic of) | -0.72 (-0.98, -0.45) |
| Canada | -0.74 (-0.91, -0.57) |
| Norway | -0.77 (-0.97, -0.57) |
| Ireland | -0.77 (-0.94, -0.59) |
| Bermuda | -0.86 (-0.97, -0.75) |
| Tuvalu | -0.91 (-1.05, -0.77) |
| Republic of Moldova | -0.93 (-1.31, -0.55) |
| France | -0.95 (-1.14, -0.77) |
| Japan | -0.96 (-1.03, -0.89) |
| Colombia | -0.97 (-1.16, -0.79) |
| Belgium | -0.97 (-1.26, -0.68) |
| Ukraine | -0.98 (-1.22, -0.74) |
| Israel | -1.06 (-1.34, -0.78) |
| Australia | -1.09 (-1.27, -0.91) |
| Argentina | -1.1 (-1.47, -0.73) |
| Grenada | -1.12 (-1.85, -0.38) |
| United States of America | -1.14 (-1.3, -0.97) |
| American Samoa | -1.15 (-1.67, -0.63) |
| Luxembourg | -1.32 (-1.62, -1.03) |
| Malta | -1.32 (-1.52, -1.13) |
| Finland | -1.37 (-1.73, -1.01) |
| Slovenia | -1.4 (-1.62, -1.18) |
| Cook Islands | -1.43 (-1.62, -1.23) |
| Philippines | -1.5 (-1.85, -1.15) |
| Bahrain | -1.86 (-2.26, -1.46) |
| Greenland | -2.05 (-2.34, -1.75) |
| Puerto Rico | -2.05 (-2.31, -1.79) |
| Iceland | -2.19 (-2.62, -1.75) |
| Switzerland | -2.35 (-2.62, -2.08) |
| Northern Mariana Islands | -2.7 (-3.2, -2.19) |
| Kyrgyzstan | -3.02 (-3.32, -2.73) |
| Guam | -4.41 (-5.45, -3.36) |
| Netherlands | -4.85 (-6.11, -3.57) |

ASDR: age-standardized death rate, EAPCs: estimated annual percentage changes, CI: confidence interval.

**Supplementary table S12. The EAPCs of age-standardized DALY Rates of 204 countries and territories from1990 to 2019 (from high to low).**

| countries and territories | EAPCs of Age-Standardized DALY Rates  (95% CI) |
| --- | --- |
| Jamaica | 6.72 (5.81, 7.63) |
| Poland | 5.14 (4, 6.28) |
| Equatorial Guinea | 4.29 (3.79, 4.79) |
|  |  |
| Estonia | 4.22 (2.4, 6.06) |
| Lesotho | 3.88 (3.4, 4.37) |
| Georgia | 3.75 (2.21, 5.3) |
| Dominican Republic | 3.66 (3.36, 3.95) |
| El Salvador | 3.57 (3.14, 4) |
| Bosnia and Herzegovina | 3.52 (3.07, 3.96) |
| Albania | 3.16 (2.72, 3.59) |
| Angola | 3.14 (2.79, 3.49) |
| North Macedonia | 3.12 (2.53, 3.71) |
| Paraguay | 3.05 (2.73, 3.37) |
| Guyana | 2.81 (1.95, 3.68) |
| Montenegro | 2.77 (2.4, 3.13) |
| Honduras | 2.73 (2.5, 2.96) |
| Ecuador | 2.72 (2.32, 3.13) |
| Democratic Republic of the Congo | 2.6 (2.3, 2.9) |
| Central African Republic | 2.51 (2.06, 2.96) |
| Eritrea | 2.34 (2.07, 2.6) |
| Guatemala | 2.31 (1.96, 2.66) |
| Mozambique | 2.28 (2.09, 2.46) |
| China | 2.25 (2, 2.51) |
| Gabon | 2.2 (1.7, 2.69) |
| Congo | 2.15 (1.85, 2.46) |
| Serbia | 2.09 (1.77, 2.41) |
| Djibouti | 1.97 (1.82, 2.11) |
| Suriname | 1.96 (1.65, 2.28) |
| Somalia | 1.92 (1.74, 2.1) |
| Indonesia | 1.91 (1.81, 2.01) |
| United Republic of Tanzania | 1.89 (1.74, 2.04) |
| Belize | 1.82 (1.52, 2.12) |
| Timor-Leste | 1.76 (1.5, 2.01) |
| Madagascar | 1.73 (1.54, 1.93) |
| Comoros | 1.68 (1.6, 1.77) |
| Cabo Verde | 1.66 (1.32, 2) |
| Bolivia (Plurinational State of) | 1.63 (1.57, 1.68) |
| Bulgaria | 1.61 (1.37, 1.84) |
| Zambia | 1.59 (1.51, 1.66) |
| Egypt | 1.53 (1.4, 1.65) |
| Yemen | 1.46 (1.36, 1.57) |
| Zimbabwe | 1.44 (1.04, 1.84) |
| Uganda | 1.41 (1.17, 1.65) |
| Botswana | 1.38 (1.08, 1.68) |
| Eswatini | 1.37 (0.74, 2) |
| Romania | 1.36 (1.13, 1.58) |
| Sudan | 1.34 (1.16, 1.52) |
| Palestine | 1.31 (0.89, 1.74) |
| Kenya | 1.27 (1.13, 1.4) |
| South Sudan | 1.19 (1.05, 1.32) |
| Viet Nam | 1.13 (1, 1.25) |
| Saint Vincent and the Grenadines | 1.11 (0.88, 1.34) |
| Nepal | 1.11 (0.87, 1.35) |
| Namibia | 1.08 (0.87, 1.29) |
| Kuwait | 1.05 (0.61, 1.49) |
| Nigeria | 1.03 (0.94, 1.13) |
| Sierra Leone | 1.02 (0.89, 1.15) |
| Libya | 1.01 (0.84, 1.19) |
| Oman | 1 (0.52, 1.48) |
| Cyprus | 1 (0.73, 1.28) |
| Peru | 1 (0.76, 1.24) |
| Lithuania | 0.97 (0.15, 1.81) |
| Gambia | 0.94 (0.75, 1.14) |
| Republic of Korea | 0.91 (0.5, 1.32) |
| Thailand | 0.86 (0.7, 1.01) |
| Sao Tome and Principe | 0.84 (0.67, 1.01) |
| Chad | 0.83 (0.72, 0.95) |
| Dominica | 0.83 (0.66, 1.01) |
| Cambodia | 0.81 (0.72, 0.91) |
| Senegal | 0.79 (0.6, 0.99) |
| Malaysia | 0.79 (0.45, 1.13) |
| Bhutan | 0.76 (0.69, 0.83) |
| Czechia | 0.73 (0.07, 1.39) |
| Barbados | 0.72 (0.66, 0.79) |
| Pakistan | 0.72 (0.49, 0.96) |
| Morocco | 0.7 (0.49, 0.91) |
| San Marino | 0.7 (0.48, 0.93) |
| Malawi | 0.69 (0.54, 0.84) |
| Antigua and Barbuda | 0.68 (0.45, 0.9) |
| Burkina Faso | 0.68 (0.54, 0.82) |
| Lebanon | 0.66 (0.4, 0.92) |
| Brunei Darussalam | 0.65 (0.47, 0.82) |
| Russian Federation | 0.65 (0.36, 0.94) |
| Saudi Arabia | 0.64 (0.31, 0.98) |
| Democratic People's Republic of Korea | 0.63 (0.51, 0.75) |
| Mexico | 0.62 (0.5, 0.73) |
| Rwanda | 0.58 (0.42, 0.75) |
| Afghanistan | 0.57 (0.51, 0.62) |
| Iraq | 0.55 (0.31, 0.79) |
| Niger | 0.55 (0.49, 0.61) |
| Liberia | 0.51 (0.3, 0.72) |
| United States Virgin Islands | 0.51 (0.26, 0.76) |
| Monaco | 0.51 (0.2, 0.81) |
| Qatar | 0.5 (0.21, 0.79) |
| Benin | 0.48 (0.4, 0.56) |
| Portugal | 0.48 (0.19, 0.77) |
| Tunisia | 0.44 (0.33, 0.56) |
| Guinea | 0.43 (0.37, 0.48) |
| Burundi | 0.42 (0.31, 0.53) |
| Azerbaijan | 0.42 (0.22, 0.62) |
| Togo | 0.4 (0.31, 0.49) |
| Cameroon | 0.39 (0.31, 0.46) |
| Mongolia | 0.38 (-0.02, 0.79) |
| Guinea-Bissau | 0.33 (0.27, 0.39) |
| Haiti | 0.3 (0.17, 0.43) |
| United Arab Emirates | 0.27 (-0.29, 0.84) |
| Côte d'Ivoire | 0.25 (0.18, 0.33) |
| Mauritius | 0.24 (-0.16, 0.64) |
| Myanmar | 0.22 (0.13, 0.31) |
| India | 0.22 (0.02, 0.43) |
| Turkmenistan | 0.18 (-0.04, 0.4) |
| Brazil | 0.18 (0.11, 0.25) |
| Bahamas | 0.15 (0.03, 0.27) |
| Solomon Islands | 0.11 (-0.04, 0.25) |
| Ethiopia | 0.11 (-0.03, 0.25) |
| Palau | 0.07 (0.03, 0.12) |
| Trinidad and Tobago | 0.05 (-0.31, 0.4) |
| South Africa | 0.05 (-0.26, 0.35) |
| Costa Rica | 0.03 (-0.24, 0.3) |
| Uruguay | 0 (-0.22, 0.22) |
| Mali | -0.01 (-0.05, 0.04) |
| Kazakhstan | -0.03 (-0.27, 0.2) |
| Saint Kitts and Nevis | -0.04 (-0.23, 0.15) |
| Uzbekistan | -0.06 (-0.27, 0.15) |
| Panama | -0.06 (-0.49, 0.38) |
| Jordan | -0.08 (-0.38, 0.22) |
| Ghana | -0.08 (-0.14, -0.01) |
| Papua New Guinea | -0.09 (-0.17, -0.02) |
| Tajikistan | -0.11 (-0.38, 0.17) |
| Micronesia (Federated States of) | -0.12 (-0.19, -0.04) |
| Slovakia | -0.14 (-0.28, -0.01) |
| Lao People's Democratic Republic | -0.16 (-0.27, -0.04) |
| Vanuatu | -0.17 (-0.4, 0.07) |
| Chile | -0.18 (-0.4, 0.03) |
| Greece | -0.21 (-0.48, 0.06) |
| Algeria | -0.24 (-0.28, -0.19) |
| Kiribati | -0.24 (-0.34, -0.15) |
| Seychelles | -0.25 (-0.32, -0.18) |
| Sweden | -0.26 (-0.54, 0.02) |
| Mauritania | -0.29 (-0.4, -0.17) |
| Germany | -0.3 (-0.5, -0.1) |
| Turkey | -0.3 (-0.44, -0.17) |
| Tonga | -0.31 (-0.54, -0.09) |
| New Zealand | -0.32 (-0.52, -0.13) |
| Marshall Islands | -0.32 (-0.42, -0.21) |
| Nauru | -0.32 (-0.45, -0.2) |
| Bangladesh | -0.34 (-0.46, -0.21) |
| Fiji | -0.35 (-0.46, -0.24) |
| Belarus | -0.36 (-0.51, -0.22) |
| Andorra | -0.38 (-0.49, -0.27) |
| Tokelau | -0.4 (-0.49, -0.31) |
| Spain | -0.4 (-0.67, -0.14) |
| Sri Lanka | -0.43 (-0.73, -0.13) |
| Syrian Arab Republic | -0.45 (-0.76, -0.13) |
| Samoa | -0.46 (-0.54, -0.37) |
| Saint Lucia | -0.47 (-0.72, -0.22) |
| Iran (Islamic Republic of) | -0.48 (-0.83, -0.13) |
| Niue | -0.51 (-0.61, -0.41) |
| Cuba | -0.53 (-0.66, -0.41) |
| Singapore | -0.61 (-0.9, -0.31) |
| Croatia | -0.63 (-1.13, -0.14) |
| Tuvalu | -0.66 (-0.76, -0.56) |
| Hungary | -0.67 (-0.95, -0.39) |
| Latvia | -0.72 (-0.99, -0.46) |
| Austria | -0.77 (-1.24, -0.29) |
| American Samoa | -0.77 (-1.2, -0.34) |
| Venezuela (Bolivarian Republic of) | -0.82 (-1.06, -0.57) |
| Ireland | -0.85 (-1.04, -0.65) |
| Italy | -0.85 (-1.05, -0.64) |
| Maldives | -0.9 (-1.11, -0.69) |
| Canada | -0.9 (-1.08, -0.72) |
| Taiwan (Province of China) | -0.91 (-1.57, -0.25) |
| Belgium | -0.94 (-1.21, -0.66) |
| Bermuda | -0.94 (-1.06, -0.83) |
| Japan | -0.96 (-1.05, -0.87) |
| Norway | -0.98 (-1.23, -0.73) |
| United Kingdom | -1.04 (-1.29, -0.79) |
| Grenada | -1.09 (-1.72, -0.45) |
| Colombia | -1.1 (-1.29, -0.91) |
| Philippines | -1.14 (-1.46, -0.81) |
| Nicaragua | -1.14 (-1.55, -0.74) |
| Denmark | -1.19 (-1.35, -1.04) |
| Armenia | -1.21 (-1.41, -1.01) |
| Republic of Moldova | -1.21 (-1.59, -0.83) |
| Ukraine | -1.22 (-1.52, -0.92) |
| Australia | -1.22 (-1.43, -1.01) |
| Israel | -1.23 (-1.52, -0.93) |
| Cook Islands | -1.28 (-1.49, -1.07) |
| Argentina | -1.28 (-1.65, -0.91) |
| France | -1.29 (-1.53, -1.04) |
| Malta | -1.41 (-1.62, -1.21) |
| Luxembourg | -1.43 (-1.75, -1.11) |
| Finland | -1.52 (-1.85, -1.18) |
| Slovenia | -1.53 (-1.76, -1.31) |
| United States of America | -1.54 (-1.73, -1.35) |
| Northern Mariana Islands | -1.88 (-2.32, -1.43) |
| Greenland | -1.91 (-2.16, -1.66) |
| Puerto Rico | -1.93 (-2.17, -1.7) |
| Iceland | -2.32 (-2.73, -1.9) |
| Bahrain | -2.41 (-2.8, -2.01) |
| Switzerland | -2.59 (-2.86, -2.33) |
| Guam | -2.74 (-3.65, -1.81) |
| Kyrgyzstan | -3.79 (-4.1, -3.47) |
| Netherlands | -4.82 (-6.06, -3.55) |

DALY: disability-adjusted life year, EAPCs: estimated annual percentage changes, CI: confidence interval.

**Supplementary Table S13. The age and gender distribution of incident cases and ASIRs of CLL in 2019.**

| age | ASIRs per 100,000 (95% UI) | | | Incident cases (95% UI) | | |
| --- | --- | --- | --- | --- | --- | --- |
|  | male | female | male-to-female ratio | male | female | male-to-female ratio |
| 20 to 24 | 0.18 (0.13, 0.23) | 0.13 (0.1, 0.17) | 1.38 | 555.1 (396.98, 698.13) | 391.05 (300.58, 499.24) | 1.42 |
| 25 to 29 | 0.16 (0.12, 0.2) | 0.15 (0.12, 0.19) | 1.07 | 496.66 (354.09, 604.88) | 456.41 (349, 583.34) | 1.09 |
| 30 to 34 | 0.19 (0.14, 0.24) | 0.18 (0.13, 0.24) | 1.06 | 590.12 (422.71, 723.38) | 532.21 (388.6, 708.79) | 1.11 |
| 35 to 39 | 0.23 (0.17, 0.28) | 0.23 (0.17, 0.29) | 1.00 | 633.55 (476.33, 770.23) | 607.83 (462.09, 774.41) | 1.04 |
| 40 to 44 | 0.36 (0.3, 0.44) | 0.35 (0.28, 0.43) | 1.03 | 895.92 (737.33, 1095.94) | 855.53 (690.75, 1064.32) | 1.05 |
| 45 to 49 | 0.66 (0.56, 0.82) | 0.62 (0.51, 0.77) | 1.06 | 1583.15 (1341.35, 1965.81) | 1462.55 (1208.39, 1803.24) | 1.08 |
| 50 to 54 | 1.33 (1.14, 1.64) | 1.28 (1.09, 1.53) | 1.04 | 2903.99 (2479.1, 3568.66) | 2803.87 (2395.82, 3351.11) | 1.04 |
| 55 to 59 | 2.68 (2.33, 3.27) | 2.27 (1.98, 2.7) | 1.18 | 4890.63 (4263.91, 5973.38) | 4272.6 (3732.5, 5088.89) | 1.14 |
| 60 to 64 | 4.54 (3.94, 5.63) | 3.54 (3.12, 4.13) | 1.28 | 6907.52 (6002.06, 8572.18) | 5681.16 (5011.6, 6627.33) | 1.22 |
| 65 to 69 | 6.73 (5.85, 8.45) | 4.87 (4.37, 5.69) | 1.38 | 8314.25 (7227.21, 10444.34) | 6578.62 (5895.83, 7685.05) | 1.26 |
| 70 to 74 | 10.16 (8.84, 12.57) | 7.02 (6.22, 8.25) | 1.45 | 8950.03 (7789.28, 11074.18) | 6948.77 (6161.51, 8163.51) | 1.29 |
| 75 to 79 | 14.32 (12.35, 18.01) | 9.84 (8.61, 11.61) | 1.46 | 8190.12 (7062.39, 10301.95) | 6871.64 (6012.99, 8110.36) | 1.19 |
| 80 to 84 | 13.95 (11.84, 17.24) | 9.14 (7.65, 10.62) | 1.53 | 4914.82 (4173.58, 6076.38) | 4494.21 (3762.08, 5222.71) | 1.09 |
| 85 to 89 | 21.34 (17.54, 26.29) | 12.92 (10.24, 15.45) | 1.65 | 3474.1 (2856.14, 4280.08) | 3512.85 (2785.81, 4202.27) | 0.99 |
| 90 to 94 | 28.71 (22.54, 35.16) | 16.83 (12.76, 20.04) | 1.71 | 1523.67 (1196.16, 1866.11) | 1944.24 (1474.14, 2314.16) | 0.78 |
| 95 plus | 35.99 (26.38, 45.49) | 22.02 (15.78, 26.5) | 1.63 | 459.39 (336.76, 580.56) | 770.06 (551.95, 926.62) | 0.60 |

ASIR: age-standardized incidence rate, UI: uncertainty interval.

**Supplementary Table S14. The age and gender distribution of death cases and ASIRs of CLL in 2019.**

| age | ASDRs per 100,000 (95% UI) | | | death cases (95% UI) | | |
| --- | --- | --- | --- | --- | --- | --- |
|  | male | female | male-to-female ratio | male | Female | male-to-female ratio |
| 20 to 24 | 0.04 (0.03, 0.05) | 0.03 (0.02, 0.03) | 1.33 | 128.68 (93.88, 161.51) | 79.85 (63.73, 98.53) | 1.61 |
| 25 to 29 | 0.04 (0.03, 0.05) | 0.03 (0.02, 0.04) | 1.33 | 120.83 (89.89, 147.53) | 93.61 (72.49, 117.25) | 1.29 |
| 30 to 34 | 0.05 (0.04, 0.06) | 0.04 (0.03, 0.05) | 1.25 | 150.71 (107.71, 185.7) | 112.43 (84.83, 142.99) | 1.34 |
| 35 to 39 | 0.06 (0.05, 0.08) | 0.06 (0.04, 0.07) | 1.00 | 170.07 (132.78, 209.02) | 153.57 (118.45, 190.7) | 1.11 |
| 40 to 44 | 0.11 (0.09, 0.13) | 0.11 (0.09, 0.14) | 1.00 | 267.51 (221.46, 328.66) | 274.73 (220.84, 338.77) | 0.97 |
| 45 to 49 | 0.19 (0.16, 0.24) | 0.2 (0.16, 0.25) | 0.95 | 450.69 (376.32, 564.07) | 466.32 (385.77, 580.11) | 0.97 |
| 50 to 54 | 0.37 (0.31, 0.44) | 0.41 (0.34, 0.5) | 0.90 | 796.35 (680.72, 967.14) | 908.07 (755.68, 1102.32) | 0.88 |
| 55 to 59 | 0.71 (0.63, 0.86) | 0.72 (0.62, 0.86) | 0.99 | 1304.21 (1154.81, 1567.41) | 1361.65 (1170.03, 1616.81) | 0.96 |
| 60 to 64 | 1.34 (1.19, 1.61) | 1.3 (1.12, 1.51) | 1.03 | 2043.14 (1813.49, 2449.46) | 2090.26 (1798.97, 2420.78) | 0.98 |
| 65 to 69 | 2.23 (1.99, 2.69) | 1.82 (1.62, 2.14) | 1.23 | 2752.58 (2457.92, 3320.69) | 2458.88 (2183.37, 2890.27) | 1.12 |
| 70 to 74 | 3.73 (3.37, 4.42) | 2.88 (2.56, 3.36) | 1.30 | 3282.45 (2964.94, 3893.29) | 2851.1 (2532.04, 3330.68) | 1.15 |
| 75 to 79 | 5.59 (5, 6.93) | 4.62 (4.09, 5.29) | 1.21 | 3200.49 (2860.95, 3962.14) | 3224.71 (2858.5, 3697.26) | 0.99 |
| 80 to 84 | 9.45 (8.17, 11.41) | 6.62 (5.69, 7.49) | 1.43 | 3330.61 (2878.17, 4020.41) | 3254.97 (2800.25, 3686.11) | 1.02 |
| 85 to 89 | 15.75 (13.11, 19.07) | 9.66 (7.68, 11.15) | 1.63 | 2565.35 (2134.58, 3104.67) | 2626.04 (2089.35, 3032.91) | 0.98 |
| 90 to 94 | 24.3 (19.16, 29.37) | 14.1 (10.83, 16.59) | 1.72 | 1289.79 (1016.83, 1558.96) | 1628.1 (1251.34, 1915.79) | 0.79 |
| 95 plus | 35.48 (26.43, 44.43) | 20.65 (15, 24.59) | 1.72 | 452.82 (337.36, 567.1) | 721.95 (524.53, 859.97) | 0.63 |

ASDR: age-standardized death rate, UI: uncertainty interval.

**Supplementary Table S15. The age and gender distribution of DALYs and age-standardized DALY Rates of CLL in 2019.**

| age | Age-Standardized DALY Rates per 100,000  (95% UI) | | | DALYs (95% UI) | | |
| --- | --- | --- | --- | --- | --- | --- |
|  | male | female | male-female ratio | male | female | male-female ratio |
| 20 to 24 | 2.93 (2.14, 3.68) | 1.87 (1.5, 2.3) | 1.57 | 8925.14 (6525.87, 11203.04) | 5526.83 (4446.04, 6790.73) | 1.61 |
| 25 to 29 | 2.55 (1.9, 3.09) | 2 (1.55, 2.49) | 1.28 | 7768.76 (5788.24, 9418.37) | 6013.76 (4667.17, 7497.76) | 1.29 |
| 30 to 34 | 2.94 (2.1, 3.6) | 2.23 (1.68, 2.84) | 1.32 | 8916.67 (6378.22, 10911.6) | 6651.43 (5019.5, 8465.15) | 1.34 |
| 35 to 39 | 3.42 (2.65, 4.19) | 3.07 (2.37, 3.84) | 1.11 | 9320.19 (7234, 11426.23) | 8252.7 (6359.37, 10296.27) | 1.13 |
| 40 to 44 | 5.29 (4.37, 6.49) | 5.47 (4.41, 6.73) | 0.97 | 13166.95 (10879.43, 16136.22) | 13397.22 (10781.4, 16469.02) | 0.98 |
| 45 to 49 | 8.4 (7.05, 10.49) | 8.67 (7.2, 10.78) | 0.97 | 20033.01 (16810.29, 25016.87) | 20411.14 (16941.22, 25386.34) | 0.98 |
| 50 to 54 | 14.63 (12.58, 17.77) | 16.24 (13.52, 19.65) | 0.90 | 31829.52 (27361.45, 38660.24) | 35592.18 (29629.3, 43077.71) | 0.89 |
| 55 to 59 | 25.34 (22.46, 30.46) | 24.97 (21.49, 29.3) | 1.01 | 46310.07 (41052.12, 55670.77) | 46998.57 (40461.17, 55161.66) | 0.99 |
| 60 to 64 | 41.14 (36.37, 49.1) | 38.73 (33.28, 44.7) | 1.06 | 62596.54 (55336.66, 74714.01) | 62115.65 (53381.53, 71683.89) | 1.01 |
| 65 to 69 | 57.77 (51.41, 69.68) | 46.09 (41, 53.94) | 1.25 | 71417.37 (63552.64, 86140.05) | 62199.68 (55335.83, 72800.48) | 1.15 |
| 70 to 74 | 80.24 (71.91, 95.52) | 60.12 (53.32, 70.38) | 1.33 | 70695.42 (63354.31, 84158.61) | 59513.78 (52779.05, 69666.8) | 1.19 |
| 75 to 79 | 97.31 (85.95, 122.3) | 76.93 (68.21, 88.63) | 1.26 | 55667.15 (49170.49, 69959.96) | 53733.1 (47641.67, 61908.7) | 1.04 |
| 80 to 84 | 119.3 (102.24, 144.06) | 82.36 (71.17, 93.91) | 1.45 | 42037.32 (36025.61, 50762.26) | 40507.65 (35005.67, 46188.41) | 1.04 |
| 85 to 89 | 154.19 (126.77, 187.72) | 94 (75.9, 109.13) | 1.64 | 25107.95 (20642.9, 30566.57) | 25565.83 (20643.09, 29681.04) | 0.98 |
| 90 to 94 | 182.14 (144.16, 220.11) | 105.7 (80.6, 124.06) | 1.72 | 9667.65 (7651.84, 11683.04) | 12207.87 (9309.63, 14328.94) | 0.79 |
| 95 plus | 191.11 (141.49, 239.41) | 110.88 (81.08, 132.56) | 1.72 | 2439.24 (1805.91, 3055.64) | 3877.25 (2835.38, 4635.52) | 0.63 |

DALY: disability-adjusted life year, UI: uncertainty interval.

**Supplementary Table S16. The age and region distribution of incident cases (95% UI) of CLL in 2019.**

|  | 20 to 24 | 25 to 29 | 30 to 34 | 35 to 39 | 40 to 44 | 45 to 49 | 50 to 54 | 55 to 59 | 60 to 64 | 65 to 69 | 70 to 74 | 75 to 79 | 80 to 84 | 85 to 89 | 90 to 94 | 95 plus |
| --- | --- | --- | --- | --- | --- | --- | --- | --- | --- | --- | --- | --- | --- | --- | --- | --- |
| High SDI | 84.64 (70.15, 98.18) | 91.44 (75.48, 107.72) | 95.79 (79.94, 115.31) | 144.96 (121.35, 171.26) | 244 (204.11, 294.88) | 594.8 (503.46, 737.54) | 1362.29 (1153.16, 1720.78) | 2877.47 (2429.76, 3790.03) | 4491.71 (3823.74, 5910.82) | 6117.73 (5213.68, 7991.58) | 7667.39 (6582.11, 9767.93) | 8088 (6830.54, 10341.76) | 5045.38 (4133.29, 6299.11) | 4202.89 (3369.95, 5086.65) | 2350.22 (1805.76, 2808.81) | 928.67 (687.49, 1124.59) |
| High-middle SDI | 281.46 (217.29, 345.28) | 316.06 (258.33, 384.04) | 414.83 (330.05, 502.35) | 436.29 (364.35, 517.34) | 582.25 (500.99, 682.24) | 956.74 (830.87, 1135.44) | 1780 (1557.53, 2067.91) | 2987.44 (2651.58, 3428.72) | 4064.59 (3650.01, 4732.19) | 4796.12 (4297.26, 5543.21) | 4547.99 (4044.86, 5159.82) | 3998.53 (3510.24, 4662.39) | 2574.19 (2162.04, 2936.57) | 1846.37 (1487.48, 2171.86) | 825.55 (644.34, 970.63) | 247.71 (184.4, 296.9) |
| Middle SDI | 390.97 (311.47, 476.23) | 398.1 (317, 486.02) | 470.11 (372.74, 586.6) | 486.29 (389.84, 601.54) | 583.94 (481.67, 708.07) | 881.37 (726.16, 1079.77) | 1464.08 (1232.23, 1759.88) | 1807.01 (1544.83, 2166.73) | 2074.4 (1770.02, 2476.69) | 2134.5 (1831.12, 2502.48) | 1769.46 (1527.82, 2100.66) | 1438.8 (1251.55, 1721.81) | 868.7 (727.9, 1014.39) | 491.67 (408.52, 585.59) | 166.8 (128.4, 205.65) | 32.74 (23.09, 43.69) |
| Low-middle SDI | 101.29 (82.35, 126.52) | 79.77 (67.04, 96.65) | 74.61 (62.35, 89.2) | 100.78 (82.43, 124.03) | 204.68 (166.45, 254.92) | 377.96 (315.14, 469.34) | 727.48 (582.89, 886.33) | 977.58 (800.75, 1183.86) | 1223.75 (1022.7, 1441.77) | 1184.91 (1001.11, 1405.41) | 1179 (1000.48, 1405.54) | 974.05 (824.83, 1161.78) | 637.59 (526.57, 758.52) | 313.34 (252.15, 380.38) | 88.18 (69.34, 108.29) | 13.38 (9.75, 16.84) |
| Low SDI | 31.21 (25.39, 38.35) | 23.55 (19.28, 28.79) | 17.49 (14.02, 21.86) | 29.74 (21.28, 41.88) | 71.25 (53.28, 97.02) | 140.78 (108.8, 185.18) | 239.21 (191.3, 300.95) | 370.94 (301.65, 458.17) | 555.14 (455.23, 672.24) | 468.57 (373.95, 574.47) | 524.11 (418.09, 636.53) | 436.26 (343.99, 528.67) | 231.49 (175.93, 290.17) | 107.73 (79.76, 143.47) | 32.56 (23.75, 43.4) | 6.08 (4.29, 8.32) |
| Global | 946.15 (792.37, 1102.37) | 953.06 (802.25, 1120.19) | 1122.32 (932.05, 1327.64) | 1241.38 (1072.17, 1434.19) | 1751.45 (1533.16, 2023.94) | 3045.7 (2699.22, 3571.06) | 5707.86 (5102.63, 6661.76) | 9163.23 (8274.06, 10604.75) | 12588.68 (11412.09, 14793.11) | 14892.87 (13423.23, 17598.23) | 15898.8 (14245.05, 18651.83) | 15061.76 (13341.5, 17821.63) | 9409.03 (8008.32, 10936.47) | 6986.95 (5683.55, 8134.18) | 3467.9 (2708.27, 4050.01) | 1229.45 (915.36, 1469.78) |

ASIR: age-standardized incidence rate, UI: uncertainty interval.

**Supplementary Table S17. The age and region distribution of death cases (95% UI) of CLL in 2019.**

|  | 20 to 24 | 25 to 29 | 30 to 34 | 35 to 39 | 40 to 44 | 45 to 49 | 50 to 54 | 55 to 59 | 60 to 64 | 65 to 69 | 70 to 74 | 75 to 79 | 80 to 84 | 85 to 89 | 90 to 94 | 95 plus |
| --- | --- | --- | --- | --- | --- | --- | --- | --- | --- | --- | --- | --- | --- | --- | --- | --- |
| High SDI | 9.3 (7.8, 10.6) | 11.14 (9.36, 12.99) | 13.02 (11.02, 15.39) | 21.31 (18.23, 24.84) | 39.3 (34.26, 46.97) | 91.9 (81.18, 114.82) | 207.19 (185.58, 258.34) | 437.88 (391.61, 557.58) | 800.18 (719.07, 1033.47) | 1269.79 (1139.78, 1636.51) | 1876.24 (1680.1, 2356.76) | 2354.84 (2069.85, 2940.56) | 2838.39 (2408.29, 3496.15) | 2694.01 (2202.37, 3239.22) | 1805.04 (1406.41, 2149.23) | 842.86 (637.72, 1018.68) |
| High-middle SDI | 44.47 (34.74, 54.66) | 53.76 (42.86, 64.42) | 75.72 (60.95, 89.74) | 86.85 (73.4, 100.81) | 129.93 (111.86, 151.87) | 210.52 (183.53, 245) | 395.99 (351.68, 453.49) | 694.18 (622.68, 785.92) | 1121.39 (1017.4, 1281.07) | 1547.22 (1405.15, 1769.82) | 1655.42 (1516.34, 1850.2) | 1657.55 (1488.12, 1896.37) | 1822.82 (1568.2, 2047.46) | 1407.12 (1173.17, 1609.45) | 725.41 (573.21, 839.62) | 247.65 (184.74, 290.36) |
| Middle SDI | 95.32 (74.86, 115.55) | 99.82 (81.21, 119.85) | 127.59 (102.06, 152.43) | 142.23 (117.63, 171.62) | 197.17 (167.41, 239.12) | 290.33 (247.01, 351.41) | 495.16 (423.18, 589.54) | 660.05 (573.99, 786.78) | 915.27 (797.88, 1068.83) | 1079.77 (940.15, 1260.94) | 1066.77 (927.8, 1230.83) | 1025.02 (896.19, 1205.57) | 897.65 (756.14, 1048.48) | 556.2 (462.91, 656.46) | 212.15 (163.36, 260.08) | 49.9 (36.02, 65.87) |
| Low-middle SDI | 40.84 (34.33, 50.66) | 34.67 (29.08, 41.91) | 34.68 (29.46, 42.7) | 52.32 (42.23, 66.68) | 122.51 (98.18, 156.07) | 222.14 (184.66, 280.67) | 432.2 (341.16, 538.02) | 599.63 (489.98, 737.59) | 846 (702.79, 1019.54) | 902.56 (753.21, 1093.39) | 1020.97 (855.91, 1228.2) | 921.58 (767.57, 1104.74) | 737.9 (600.6, 888.75) | 389.91 (311.21, 480.64) | 125.42 (99, 156.22) | 22.96 (17.1, 28.92) |
| Low SDI | 18.52 (15.22, 22.68) | 14.97 (12.38, 18.36) | 12.06 (9.68, 14.86) | 20.83 (15.31, 28.01) | 53.17 (39.35, 69.72) | 101.87 (80.02, 129.33) | 173.27 (137.9, 214.16) | 273.02 (221.88, 332.34) | 449 (365.72, 547.71) | 409.94 (329.46, 502.6) | 511.55 (409.04, 619.74) | 463.24 (360.69, 565.07) | 285.44 (218.25, 358.8) | 141.27 (104.3, 188.92) | 48.17 (34.87, 63.91) | 10.74 (7.51, 14.67) |
| Global | 208.53 (173.74, 243.72) | 214.43 (182.42, 249.11) | 263.14 (219.39, 301.08) | 323.64 (280.55, 367.47) | 542.24 (476.22, 618.14) | 917.01 (817.98, 1071.14) | 1704.42 (1524.89, 1951.43) | 2665.86 (2438.89, 3030.99) | 4133.4 (3795.62, 4669.09) | 5211.46 (4815.35, 5942.7) | 6133.56 (5658.93, 6972.7) | 6425.2 (5830.34, 7341.17) | 6585.58 (5717.19, 7510.85) | 5191.39 (4347.71, 6005.17) | 2917.89 (2297.18, 3374.06) | 1174.77 (881.52, 1404.87) |

ASDR: age-standardized death rate, UI: uncertainty interval.

**Supplementary Table S18. The age and region distribution of DALYs (95% UI) of CLL in 2019.**

|  | 20 to 24 | 25 to 29 | 30 to 34 | 35 to 39 | 40 to 44 | 45 to 49 | 50 to 54 | 55 to 59 | 60 to 64 | 65 to 69 | 70 to 74 | 75 to 79 | 80 to 84 | 85 to 89 | 90 to 94 | 95 plus |
| --- | --- | --- | --- | --- | --- | --- | --- | --- | --- | --- | --- | --- | --- | --- | --- | --- |
| High SDI | 671.57 (560.48, 770.25) | 743.78 (621.89, 869.58) | 797.07 (670.81, 940.83) | 1209.37 (1028.58, 1409.63) | 2020.51 (1755.21, 2412.36) | 4284.54 (3780.72, 5343.81) | 8765.84 (7799.63, 10869.88) | 16544.6 (14678.65, 20999.24) | 26001.74 (22937.76, 33223.1) | 34921.89 (30982.05, 44981.33) | 42654.77 (37701.43, 53713.72) | 42808.59 (37181.49, 53627.41) | 36612.08 (30983, 45044.58) | 26966 (21859.56, 32223.12) | 13727.54 (10538.6, 16362.01) | 4533.02 (3425.27, 5478.92) |
| High-middle SDI | 3136.31 (2436.54, 3836.06) | 3507.95 (2826.78, 4174.59) | 4541.88 (3686.16, 5351.96) | 4803.72 (4069.05, 5558.87) | 6502.88 (5608.63, 7597.19) | 9501.66 (8246.66, 11017.57) | 16074.89 (14257.87, 18325.75) | 24853.26 (22267.22, 28236.73) | 34501.19 (31077.85, 39457) | 40087.6 (36180.11, 45743.87) | 35441.5 (32244.09, 39962.1) | 28371.81 (25251.54, 32886.53) | 22793.59 (19593.74, 25656.01) | 13658.12 (11274.01, 15684.23) | 5398.85 (4253.75, 6222.16) | 1337.75 (1003.24, 1568.63) |
| Middle SDI | 6617.06 (5223.01, 8006.75) | 6414.67 (5239.07, 7678.31) | 7531.86 (6023.27, 9000.09) | 7706.36 (6456.06, 9264.15) | 9668.99 (8211.59, 11676.17) | 12812.08 (10920.81, 15408.58) | 19526.34 (16803.71, 23317.1) | 22795.39 (19825.12, 27103.61) | 27124.2 (23668.71, 31582.52) | 26972.34 (23592.43, 31398.84) | 21855.39 (18974.43, 25319.16) | 16579.66 (14393.22, 19512.96) | 10859.73 (9180.07, 12694.75) | 5156.28 (4304.29, 6119.49) | 1512.01 (1159.45, 1848.09) | 263.23 (188.99, 346.86) |
| Low-middle SDI | 2774.01 (2331.37, 3428.75) | 2178.5 (1828.31, 2630.38) | 2002.81 (1702.64, 2467.99) | 2758.52 (2233.18, 3505.94) | 5842.61 (4690.52, 7444.79) | 9500.51 (7917.78, 11972.23) | 16469.52 (13029.79, 20379.19) | 20026.92 (16372.8, 24510.44) | 24251.89 (20126.29, 29126.53) | 21763.73 (18227.69, 26251.7) | 20171.94 (16969.06, 24229.77) | 14400.45 (12041.85, 17290.05) | 8830.23 (7202.41, 10619.26) | 3574.27 (2847.9, 4411.48) | 885.04 (697.2, 1099.67) | 121.64 (90.65, 153.11) |
| Low SDI | 1248.17 (1024.61, 1527.22) | 933.46 (772.12, 1146.66) | 690.67 (555.32, 853.93) | 1089.73 (802.51, 1464.3) | 2520.85 (1868.99, 3303.41) | 4334.69 (3403.95, 5498) | 6561.09 (5212.95, 8149.3) | 9050.41 (7359.36, 11065.32) | 12785.69 (10396.09, 15547.49) | 9815.53 (7875.68, 12097.59) | 10030.07 (7998.8, 12115) | 7189.11 (5609.46, 8736.36) | 3407.01 (2600.17, 4307.53) | 1291.14 (952.46, 1726.24) | 339.48 (245.74, 453.05) | 57.27 (40.13, 78.23) |
| Global | 14451.97 (12091.08, 16898.35) | 13782.52 (11756.08, 15968.96) | 15568.1 (12986.86, 17800.18) | 17572.88 (15214.24, 19973.85) | 26564.17 (23313.86, 30099.13) | 40444.16 (35999.5, 47064.57) | 67421.7 (60370.8, 76745.15) | 93308.64 (85161.09, 106687.1) | 124712.2 (114275, 141864.41) | 133617.05 (123002.2, 153034.77) | 130209.2 (119074.89, 148980.12) | 109400.24 (98759.45, 125769.57) | 82544.97 (71293.87, 94056.68) | 50673.78 (42229.3, 58556.07) | 21875.53 (17212.48, 25456.82) | 6316.49 (4747.45, 7571.2) |

DALY: disability-adjusted life year, UI: uncertainty interval.
